# Global Impact of Modifiable Risk Factors on Cardiovascular Disease and Mortality

**DOI:** 10.1056/NEJMoa2206916

**Published:** 2023-08-26

**Authors:** Christina Magnussen, Francisco M. Ojeda, Darryl P. Leong, Jesus Alegre-Diaz, Philippe Amouyel, Larissa Aviles-Santa, Dirk De Bacquer, Christie M. Ballantyne, Antonio Bernabe-Ortiz, Martin Bobak, Hermann Brenner, Rodrigo M. Carrillo-Larco, James de Lemos, Annette Dobson, Marcus Dörr, Chiara Donfrancesco, Wojciech Drygas, Robin P. Dullaart, Gunnar Engström, Marco M. Ferrario, Jean Ferrieres, Giovanni de Gaetano, Uri Goldbourt, Clicerio Gonzalez, Guido Grassi, Allison M. Hodge, Kristian Hveem, Licia Iacoviello, M. Kamran Ikram, Vilma Irazola, Modou Jobe, Pekka Jousilahti, Pontiano Kaleebu, Maryam Kavousi, Frank Kee, Davood Khalili, Wolfgang Koenig, Anna Kontsevaya, Kari Kuulasmaa, Karl J. Lackner, David M. Leistner, Lars Lind, Allan Linneberg, Thiess Lorenz, Magnus Nakrem Lyngbakken, Reza Malekzadeh, Sofia Malyutina, Ellisiv B. Mathiesen, Olle Melander, Andres Metspalu, J. Jaime Miranda, Marie Moitry, Joseph Mugisha, Mahdi Nalini, Vijay Nambi, Toshiharu Ninomiya, Karen Oppermann, Eleonora d’Orsi, Andrzej Pajak, Luigi Palmieri, Demosthenes Panagiotakos, Arokiasamy Perianayagam, Annette Peters, Hossein Poustchi, Andrew M. Prentice, Eva Prescott, Ulf Risérus, Veikko Salomaa, Susana Sans, Satoko Sakata, Ben Schöttker, Aletta E. Schutte, Sadaf G. Sepanlou, Sanjib Kumar Sharma, Jonathan E. Shaw, Leon A. Simons, Stefan Söderberg, Abdonas Tamosiunas, Barbara Thorand, Hugh Tunstall-Pedoe, Raphael Twerenbold, Diego Vanuzzo, Giovanni Veronesi, Julia Waibel, S. Goya Wannamethee, Masafumi Watanabe, Philipp Wild, Yao Yao, Yi Zeng, Andreas Ziegler, Stefan Blankenberg

**Affiliations:** 1 University Heart and Vascular Center Hamburg, University Medical Center Hamburg-Eppendorf, Hamburg, Germany; 2 German Centre for Cardiovascular Disease (DZHK), Partner site Hamburg/Kiel/Luebeck, Hamburg, Germany; 3 Center for Population Health Innovation (POINT), University Heart and Vascular Center Hamburg, University Medical Center Hamburg-Eppendorf, Hamburg, Germany; 4 Department of Medicine (Cardiology), McMaster University, Hamilton, Canada; 5 Experimental Medicine Research Unit from the School of Medicine, National Autonomous University of Mexico (UNAM), Mexico City; 6 Univ. Lille, Inserm, Centre Hosp. Univ Lille, Institut Pasteur de Lille, UMR1167 - RID-AGE LabEx DISTALZ - Risk factors and molecular determinants of aging-related diseases, F-59000 Lille, France; 7 Division of Clinical and Health Services Research, National Institute on Minority Health and Health Disparities at the National Institutes of Health, Bethesda, MD, USA; 8 Department of Public Health and Primary Care, Ghent University, Ghent, Belgium; 9 Department of Medicine, Baylor College of Medicine, USA; 10 CRONICAS Center of Excellence in Chronic Diseases, Universidad Peruana Cayetano Heredia, Lima, Peru; 11 Department of Epidemiology and Public Health, University College London, London, UK; 12 Division of Clinical Epidemiology and Aging Research, German Cancer Research Center (DKFZ), Heidelberg, Germany; 13 Network Aging Research (NAR), Heidelberg University, Heidelberg, Germany; 14 Emory Global Diabetes Research Center and Hubert Department of Global Health Rollins School of Public Health, Emory University, Atlanta, USA; 15 Division of Cardiology, Department of Internal Medicine, University of Texas Southwestern Medical Center, Dallas, USA; 16 School of Public Health, University of Queensland, Brisbane, QLD, Australia; 17 Department of Internal Medicine B, University Medicine Greifswald, Greifswald, Germany; 18 German Centre for Cardiovascular Disease (DZHK), Partner Site Greifswald, Greifswald, Germany Cardiovascular Disease (DZD), Site Greifswald, Greifswald, Germany; 19 Department of Cardiovascular, Endocrine-metabolic Diseases and Aging, Istituto Superiore di Sanità-ISS, Rome, Italy; 20 Department of Epidemiology, Cardiovascular Disease Prevention and Health Promotion, National Institute of Cardiology, Warsaw, Poland; 21 Lazarski University, Warsaw, Poland; 22 Department of Endocrinology, University Medical Center Groningen, University of Groningen, Groningen, The Netherlands; 23 Lund University, Department of Clinical Sciences Malmö, Malmö, Sweden; 24 Research Center in Epidemiology and Preventive Medicine, Department of Medicine and Surgery, University of Insubria, Varese, Italy; 25 Department of Cardiology, INSERM UMR 1295, Toulouse Rangueil University Hospital, 31059 Toulouse, France; 26 Department of Epidemiology and Prevention, IRCCS Neuromed, Pozzilli (IS), Italy; 27 Tel Aviv University School of Public Health department of Epidemiology Tel Aviv University School of Public Health department of Epidemiology; 28 Centro de Estudios en Diabetes AC. Centro de Investigacion en Salud Poblacional. Instituto Nacional de Salud Publica; 29 Clinica Medica, University of Milano-Bicocca, Milan, Italy; 30 Cancer Epidemiology Division, Cancer Council Victoria, 615 St Kilda Road, Melbourne, Victoria 3004, Australia; 31 Centre for Epidemiology and Biostatistics, Melbourne School of Population and Global Health, The University of Melbourne, Victoria 3010, Australia; 32 HUNT Research Center, Department of Public Health and Nursing, Norwegian University of Science and Technology (NTNU), Levanger, Norway; 33 K.G. Jebsen Center for Genetic Epidemiology, Department of Public Health and Nursing, Norwegian University of Science and Technology (NTNU), Trondheim, Norway; 34 Departments of Neurology & Epidemiology, Erasmus MC, University Medical Center Rotterdam, Rotterdam, The Netherlands; 35 Department of Chronic Diseases, Institute for Clinical Effectiveness and Health Policy, Buenos Aires, Argentina; 36 MRC Unit The Gambia @ London School of Hygiene & Tropical Medicine, Banjul, The Gambia; 37 Department of Public Health and Welfare, Finnish Institute for Health and Welfare (THL), Helsinki, Finland; 38 MRC/UVRI and LSHTM Uganda Research Unit; 39 Department of Epidemiology, Erasmus MC University Medical Center Rotterdam, Rotterdam, The Netherlands; 40 Centre for Public Health, Queens University Belfast; 41 Prevention of Metabolic Disorders Research Center, Research Institute for Endocrine Sciences, Shahid Beheshti University of Medical Sciences, Tehran, Iran; 42 German Centre for Cardiovascular Research (DZHK), partner site Munich Heart Alliance, Munich, Germany; 43 German Heart Centre, Technical University of Munich, Munich, Germany; 44 Institute of Epidemiology and Medical Biometry, University of Ulm, Ulm, Germany; 45 National research center for therapy and preventive medicine of the Ministry of Healthcare of the Russian Federation, Moscow, Russia; 46 Institute of Clinical Chemistry and Laboratory Medicine, University Medical Center of the Johannes Gutenberg-University Mainz, Mainz, Germany; 47 DZHK (German Center for Cardiovascular Research), partner site RhineMain, Mainz, Germany; 48 University Heart & Vascular Center Frankfurt, Frankfurt/Main, Germany and German Centre for Cardiovascular Disease (DZHK), Partner site Rhein/Main, Frankfurt, Germany; 49 Department of Medical Sciences, Uppsala, Sweden; 50 Department of Clinical Medicine, Faculty of Health and Medical Sciences, University of Copenhagen, Copenhagen, Denmark; 51 Center for Clinical Research and Prevention, Bispebjerg/Frederiksberg Hospital, Copenhagen, Denmark; 52 Department of Cardiology, Division of Medicine, Akershus University Hospital, Lørenskog, Norway; 53 K.G. Jebsen Center for Cardiac Biomarkers, Institute of Clinical Medicine, Faculty of Medicine, University of Oslo, Oslo, Norway; 54 Liver and Pancreaticobiliary Disease Research Center, Digestive Disease Research Institute, Shariati Hospital, Tehran University of Medical Sciences, Tehran, Iran; 55 Digestive Oncology Research Center, Digestive Disease Research Institute, Shariati Hospital, Tehran University of Medical Sciences, Tehran, Iran; 56 Digestive Disease Research Center, Digestive Disease Research Institute, Shariati Hospital, Tehran University of Medical Sciences, Tehran, Iran; 57 Research Institute of Internal and Preventive Medicine, Branch of ‘Federal Research Center Institute of Cytology and Genetics’ (IC&G), Siberian Branch of RAS, Novosibirsk, Russia; 58 Department of Clinical Medicine, UiT The Arctic University of Norway, Tromsø, Norway; 59 Estonian Genome Center, Institute of Genomics, University of Tartu, Tartu, Estonia; 60 Sydney School of Public Health, Faculty of Medicine and Health, University of Sydney, Sydney, Australia; 61 Department of Public health, Strasbourg University Hospital, University of Strasbourg, Strasbourg, France; 62 Metabolic Epidemiology Branch, Division of Cancer Epidemiology and Genetics, National Cancer Institute, NIH, Bethesda, Maryland, USA; 63 Michael E DeBakey Veterans Affairs hospital and Baylor College of Medicine, Houston, USA; 64 Department of Epidemiology and Public Health, Graduate School of Medical Sciences, Kyushu University, Fukuoka, Japan; 65 Medicine School, University of Passo Fundo, Passo Fundo, Rio Grande do Sul, Brazil; 66 Department of Public Health, Postgraduate Program in Public Health, Federal University of Santa Catarina, Florianopolis, Brazil; 67 Department of Epidemiology and Population Studies, Institute of Public Health, Faculty of Health Sciences, Jagiellonian University Medical College, Poland; 68 School of Health Sciences and Education, Harokopio University, Athens, Greece; 69 National Council of Applied Economic Research (NCAER), Delhi, India; 70 International Institute for Population Sciences, Mumbai, India; 71 Institute of Epidemiology, Helmholtz Zentrum München - German Research Center for Environmental Health, Neuherberg, Germany; 72 Chair of Epidemiology, Institute for Medical Information Processing, Biometry and Epidemiology, Medical Faculty, Ludwig-Maximilians-Universität München, Munich, Germany; 73 Department of Cardiology, Bispebjerg Hospital, University of Copenhagen, Denmark; 74 Department of Public Health and Caring Sciences, Clinical Nutrition and Metabolism, Uppsala University, Uppsala, Sweden; 75 Catalan Department of Health, Barcelona, Spain; 76 The School of Population Health, University of New South Wales; The George Institute for Global Health, Sydney, Australia; 77 Hypertension in Africa Research Team (HART), SAMRC Unit for Hypertension and Cardiovascular Disease, North-West University, Potchefstroom, South Africa; 78 Department of Internal Medicine, BP Koirala Institute of Health Sciences, Dharan, Nepal; 79 Baker Heart and Diabetes Institute, Melbourne, Australia; 80 University of New South Wales, Australia; 81 Department of Public Health and Clinical Medicine, University of Umea, Umea, Sweden; 82 Laboratory of Population Studies, Institute of Cardiology, Kaunas, Lithuania; Department of Preventive Medicine, Faculty of Public Health, Lithuanian University of Health Sciences, Kaunas, Lithuania; 83 German Center for Diabetes Research (DZD), Partner Munich-Neuherberg, Neuherberg, Germany; 84 Cardiovascular Epidemiology Unit, Institute of Cardiovascular Research, University of Dundee, Dundee, Scotland, UK; 85 MONICA-FRIULI Study Group, Udine, Italy; 86 Research Department of Primary Care and Population Health, University College London, London, UK; 87 Global Center of Excellence Program Study Group, Yamagata University School of Medicine, Yamagata, Japan; 88 University Medical Center of the Johannes Gutenberg-University Mainz, Mainz, Germany; 89 China Center for Health Development Studies, Peking University, Beijing, China; 90 Key Laboratory of Epidemiology of Major Diseases (Peking University), Ministry of Education, Beijing, China; 91 Center for the Study of Aging and Human Development and Geriatrics Division, Medical School of Duke University, Durham, NC, US; 92 Cardio-CARE, Davos, Switzerland; 93 School of Mathematics, Statistics and Computer Science, University of KwaZulu-Natal, Pietermaritzburg, South Africa; 94 Swiss Institute of Bioinformatics, Lausanne, Switzerland

## Abstract

**Background:**

Five modifiable risk factors are associated with cardiovascular disease (CVD) and all-cause mortality. The regional and sex-specific prevalence of these modifiable risk factors and their impact on CVD and all-cause mortality have not been evaluated using individual-level data.

**Methods:**

The Global Cardiovascular Risk Consortium harmonized individual-level data from 112 cohort studies conducted in 34 countries and 8 geographic regions. Associations between body-mass index, systolic blood pressure, non-high-density lipoprotein cholesterol, smoking, and diabetes with incident CVD and all-cause mortality were examined using Cox regression analyses and stratified by geographic region, age and sex. Population-attributable fractions were estimated for 10-year incident CVD and all-cause mortality.

**Results:**

Among 1,518,028 individuals (54.1% women, median age 54.4 years), there were regional variations in the prevalence of the five modifiable risk factors. Incident CVD occurred in 80,596 individuals (median and maximum follow-up, 7.3 and 47.3 years, respectively) and 177,369 individuals died (median and maximum follow-up, 8.7 and 47.6 years, respectively). Aggregate global CVD population-attributable fractions were 57.2% (95% confidence interval [CI], 52.4 to 62.1) in women and 52.6% (95% CI, 49.0 to 56.1) in men for all risk factors combined. Aggregate global all-cause mortality population-attributable fractions were 22.2% in women and 19.1% in men.

**Conclusions:**

Harmonized individual-level data from a global cohort found that 57.2% of incident CVD in women and 52.6% in men, and 22.2% of deaths in women and 19.1% in men may be attributable to five modifiable risk factors. The prevalence and impact of these risk factors on incident CVD and all-cause mortality varies by sex and across geographic regions.

ClinicalTrials.gov number NCT05466825

## INTRODUCTION

Cardiovascular diseases (CVD) are the most common non-communicable diseases worldwide and account for approximately one third of all deaths globally.^1^ Modifiable risk factors like elevated body-mass index, blood pressure, low-density lipoprotein cholesterol, smoking tobacco and diabetes account for a proportion of prevalent and incident CVD; however, the proportion varies according to the populations studied and the methods used to study these populations.^2,3^ Contemporary risk scores^4,5,6^ use these risk factors, but with different weightings, to estimate 10-year CVD risk. These cardiovascular risk factors are also differentially related to cardiovascular and non-cardiovascular outcomes. While tobacco consumption is strongly associated with premature mortality, elevated blood pressure and cholesterol are more specifically related to CVD.^7^

A tailored reduction in the burden of CVD and all-cause mortality for persons and populations can be achieved with better understanding of the region- and sex-specific associations of these cardiovascular risk factors with CVD development. The Global Cardiovascular Risk Consortium analyzed a global harmonized individual-level dataset of population-based cohorts to overcome the limitations of summary data and methodological heterogeneity.

## Methods

### Study Design and Oversight

The study was designed by the Global Cardiovascular Risk Consortium Management Group whose members are outlined in the Supplementary Appendix (available online with the full text of this article at NEJM.org). Data were gathered by the Hamburg Data Center. Analyses were performed by FOE and reviewed within the Global Cardiovascular Risk Consortium Statistical Working Group (see the Supplementary Appendix). The first draft of the manuscript was prepared by CM, FOE and SB and reviewed and edited by all authors. All authors jointly agreed to submit the manuscript for publication and vouch for the accuracy and completeness of the data. The study had no sponsor.

### Study population

We pooled and harmonized individual-level data from 1,518,028 individuals in 112 cohort studies conducted in eight geographic regions (North America, Latin America, Western Europe, Eastern Europe and Russia, North Africa and Middle East, sub-Saharan Africa, Asia, and Australia) participating in the Global Cardiovascular Risk Consortium. Data were harmonized applying the variable definitions used by the MONICA/MORGAM project.^8^ The studies that were not part of the MORGAM project received a list of variables for the study with definitions and were asked to provide those data. A description of each cohort, including local ethics committee information is provided in the Supplementary Appendix. The cohorts for inclusion in the Global Cardiovascular Risk Consortium were selected based on literature review, existing collaborations among investigators, and the availability of the variables of interest (Table S1). The study flow is described in the Supplementary Appendix and is shown in Fig. S1.

### Cardiovascular risk factors and outcome definitions

Five risk factors, body-mass index, systolic blood pressure, non-high-density lipoprotein cholesterol (non-HDL cholesterol), current smoking, and diabetes, were assessed in the study because of the heterogeneity of their effects on CVD and all-cause mortality, widespread availability in the population, and they can be modified with interventions. Information on these five modifiable risk factors was collected at baseline according to the protocols of the respective studies included in the Global Cardiovascular Risk Consortium. The standardized definitions used to classify CVD events are presented in Table S2 and the representativeness of the study population is shown in Table S3.

### Statistical analysis

Missing data were imputed by multiple imputation using chained equations (Table S4).^9^ Both crude and age- and sex-standardized baseline characteristics were calculated by region. Direct standardization was used, using age and sex distribution of the Global Cardiovascular Risk Consortium as the standard. Age-standardized event rates stratified by region were also estimated and reported per 1000 person-years. Cumulative incidence curves were generated for CVD and all-cause mortality. Associations between risk factors and outcome events were evaluated using a two-stage random effects multivariate individual-participant data meta-analysis.^10^ Sex-specific Cox models, with age as the time scale,^11^ were computed for each study, then coefficients pooled across studies, by region as well as globally. Covariates (body-mass index, systolic blood pressure, non-HDL cholesterol, current smoking, diabetes, and use of antihypertensive medications) were included simultaneously in the models. Both linear and restricted cubic spline models for continuous covariates and models allowing for time-varying effects were performed. Models including use of lipid-lowering medications, using those studies where this information was available, were also computed (these data were missing for approximately 20% of participants).

For the five risk factors, region- and sex-specific population-attributable fractions for the 10-year incidence of CVD and all-cause mortality was estimated (see the Supplementary Appendix). The approach of Laaksonen and colleagues,^12^ which takes into account the time-to-event nature of the data, was applied to calculate population-attributable fractions. Weibull models were used in the estimation and their distributional assumptions were assessed graphically. Reference categories for the risk factors and descriptions of the cohort studies are provided in the Supplementary Appendix. Population-attributable fractions were estimated for the hypothetical scenario in which a single risk factor was set to the reference category and also for the scenario in which all risk factors were set simultaneously to their respective reference categories.

All models used in the associations and population-attributable fractions analyses were computed after excluding the first year of follow-up (one-year landmark analysis). Two-year landmark analyses were performed as sensitivity analyses. Confidence interval widths have not been adjusted for multiple comparisons and should not be used in place of hypothesis testing. All analyses were performed using R, version 4.1.3.^13^ A detailed description of the statistical methods is provided in the Supplementary Appendix.

## Results

### Participant characteristics and risk factor prevalence

The baseline examination for all cohorts included in the Global Cardiovascular Risk Consortium took place between 1963 and 2020. In the age- and sex-standardized analysis of 1,518,028 individuals (54.1% women, median age 54.4 years), the median body-mass index was 26.4 kg per square meter (interquartile range, 23.7 to 29.7), systolic blood pressure was 130 mm Hg (interquartile range, 118 to 144), non-HDL cholesterol was 156.9 mg/dL (interquartile range, 128.8 to 187.9), 21.6% were current smokers and 8.3% had diabetes. The prevalence of the five risk factors and the use of anti-hypertensive and lipid-lowering medications across geographic regions standardized by age and sex are shown in Table 1 and Table S5. Baseline characteristics without age and sex standardization (Table S6) and risk factor distributions according to sex are shown in Tables S7 and S8. The prevalence of modifiable risk factors among contemporary national health examination surveys, which were used for the population-attributable fraction analyses, is shown in Tables S9 and S10 and S11.

### Cardiovascular disease and all-cause mortality rates

The median follow-up among participants for incident CVD was 7.3 years (interquartile range, 5.9 to 11.8) and 8.7 years (interquartile range, 7.0 to 15.9) years for all-cause mortality. The follow-up times for each of the individual cohorts are provided in Table S12. A total of 80,596 CVD events (30,033 in women and 50,563 in men) and 177,369 deaths from any cause (78,608 in women and 98,761 in men) were observed during the follow-up period (Table 2). The age and sex standardized 10-year CVD event rates were 10.3% in North America, 7.8% in North Africa and the Middle East, 7.7% in Eastern Europe and Russia, 5.4% in Western Europe and 3.4% in Asia. Global 10-year event rates in women were 4.0% and 7.8% in men (Table 2). Women appeared to develop CVD at older ages than men (Fig. S2). The age-standardized 10-year all-cause mortality rate was 30.2% in sub-Saharan Africa, 13.8% in Eastern Europe and Russia, 13.7% in Asia and 5.8% in Australia (Table 2).

### Modifiable risk factors and cardiovascular disease and all-cause mortality

**Figure 1.**
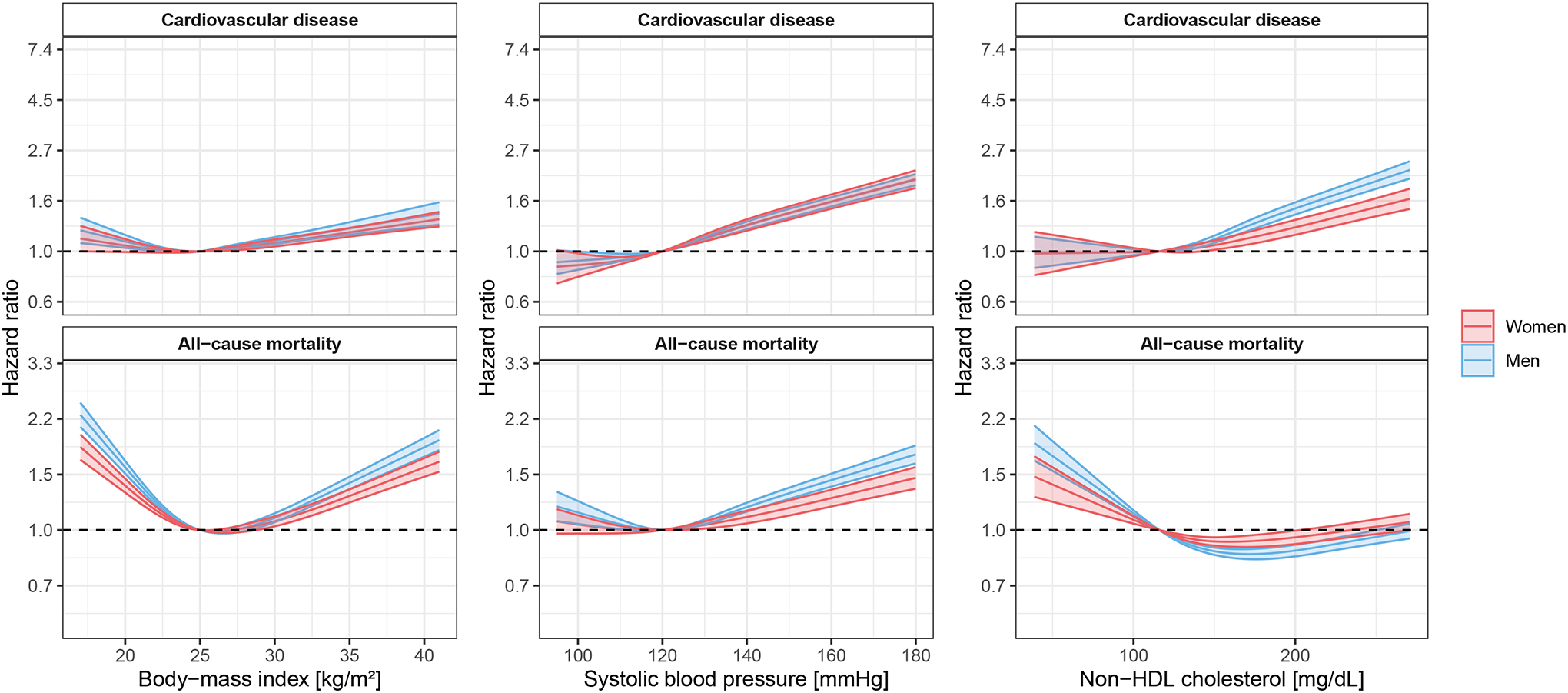
Associations of continuous risk factors with cardiovascular disease and all-cause mortality allowing for non-linear effects. Global analyses. Individuals with cardiovascular disease at baseline were excluded. Age was used as the time scale. All five risk factors considered were included in the models together with use of antihypertensive medications. A one-year landmark analysis was performed. Confidence interval widths have not been adjusted for multiplicity and should not be used in place of hypothesis testing. To convert the values for non-HDL cholesterol from milligrams per deciliter (mg/dL) to millimoles per liter (mmol/L), multiply by 0.02586.

**Figure 2.**
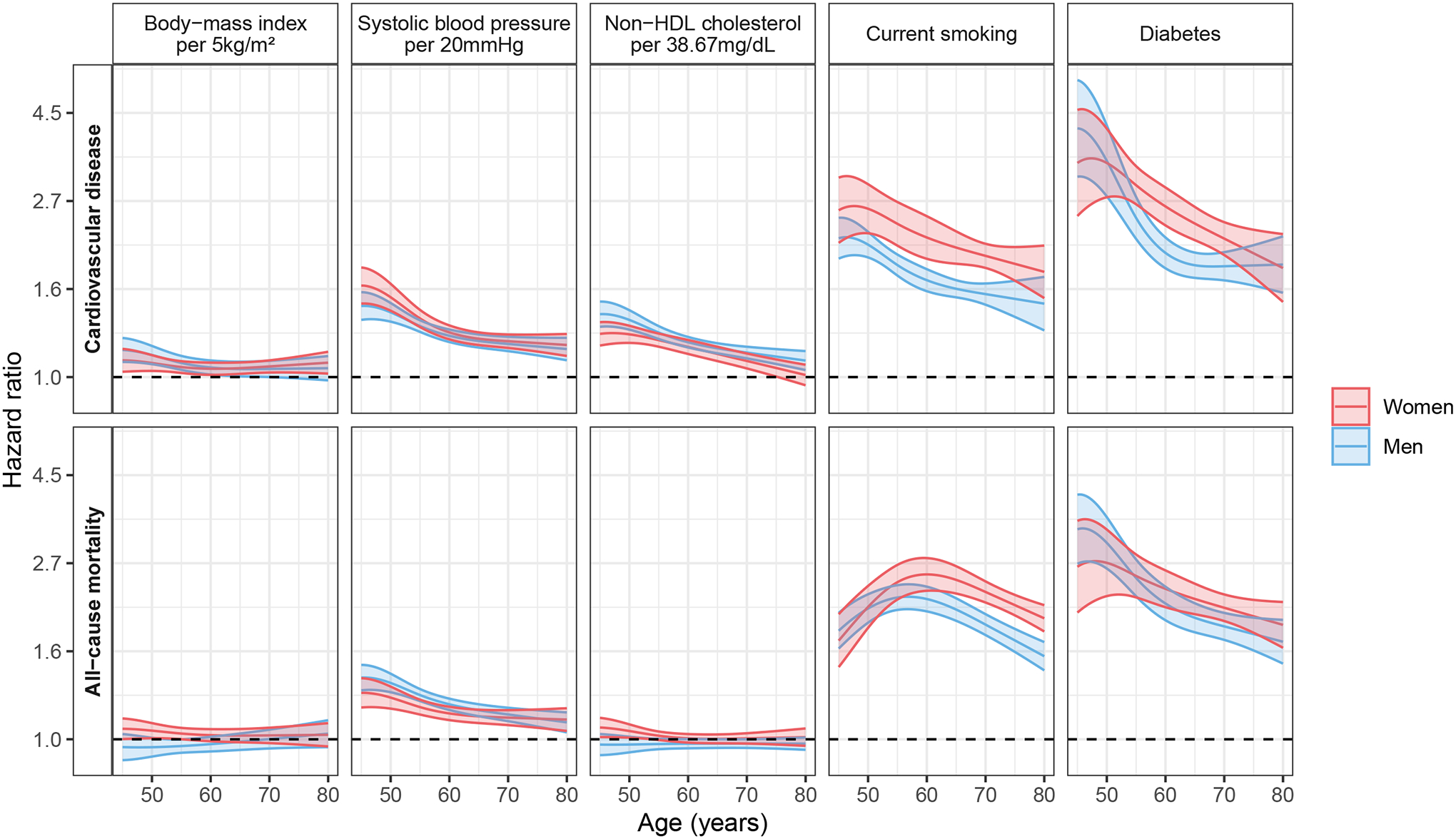
Associations of risk factors with cardiovascular disease and all-cause mortality allowing for effects to change with age. Global analyses. Individuals with cardiovascular disease at baseline were excluded. Age was used as the time scale. All five risk factors considered were included in the models together with use of antihypertensive medications. A one-year landmark analysis was performed. Global estimates are presented. Confidence interval widths have not been adjusted for multiplicity and should not be used in place of hypothesis testing. To convert the values for non-HDL cholesterol from milligrams per deciliter (mg/dL) to millimoles per liter (mmol/L), multiply by 0.02586.

Risk factor hazard ratios for CVD and all-cause mortality by geographic region and sex are shown in Table S13 and Fig. S3–S7). Subdistribution hazard ratios were similar to hazard ratios (Table S14). Modeling continuous risk factors and allowing for non-linear effects, the association of risk factors with CVD and with all-cause mortality is shown in Figure 1 and Fig. S4. The observed associations appeared similar in a two-year landmark analysis (Fig. S5). Broad sensitivity analyses of the Cox models assuming linear effects when performing two-year landmark analyses or when analyzing only cohorts where the study was conducted starting in the year 2000 or later, when restricted to individuals with information on lipid-lowering medication use, when using an alternative definition of CVD (composite of fatal and non-fatal myocardial infarction, ischemic or hemorrhagic stroke, and cardiovascular death) also appeared similar (Tables S15 through S18 and Fig. S6). Unadjusted risk factor hazard ratios for CVD and all-cause mortality are shown in Table S19. For both CVD and all-cause mortality, the association with body-mass index appeared consistent across all ages, while the associations with systolic blood pressure, current smoking (after a steady increase up to the second half of life for all-cause mortality) and diabetes decreased over the age range (Figure 2 and Fig. S7). The relationship between non-HDL cholesterol and CVD seemed to decline with age but appeared stable for all-cause mortality (Figure 2 and Fig. S7).

### Proportion of preventable cardiovascular disease and all-cause mortality

**Figure 3.**
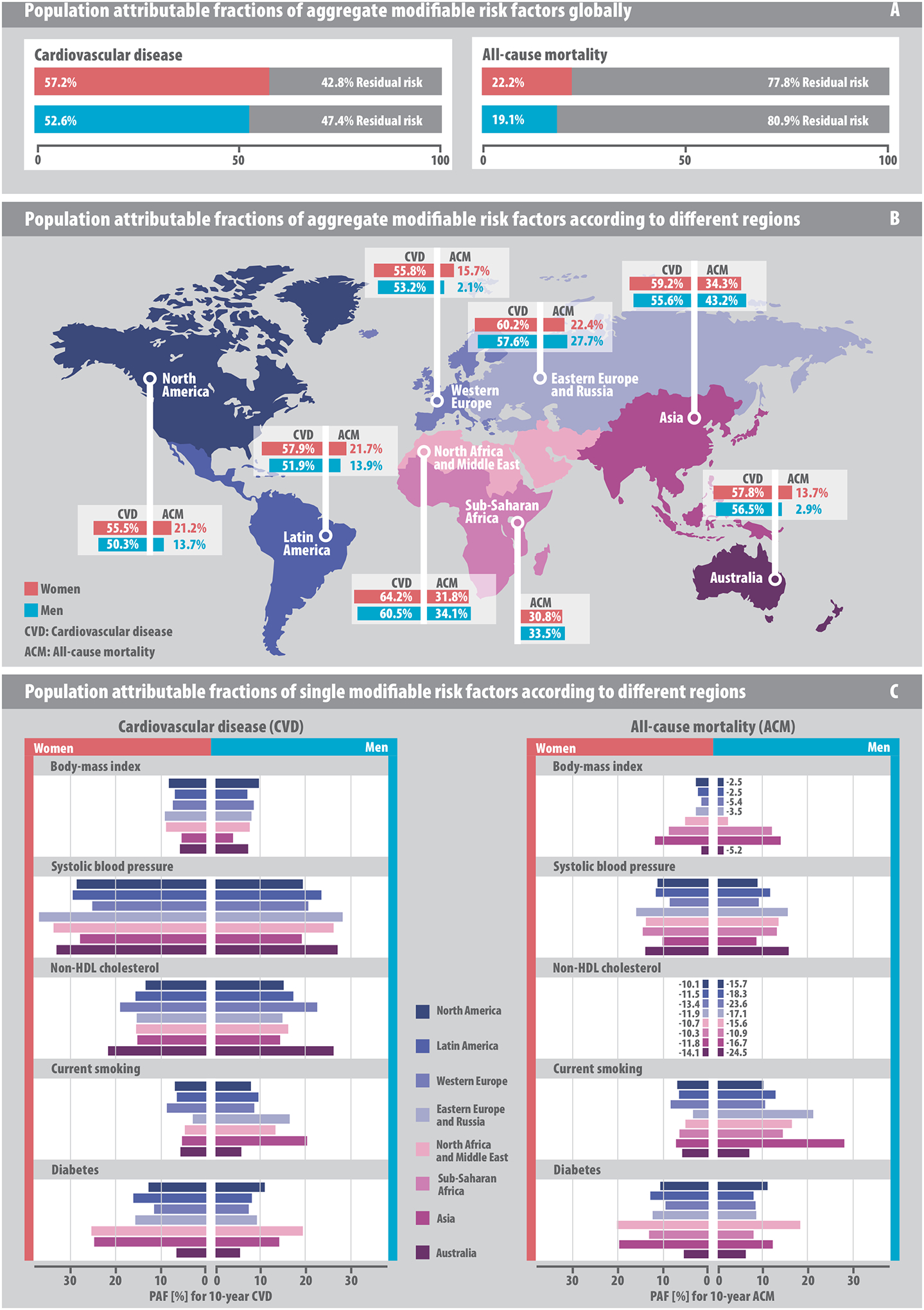
Population-attributable fractions of risk factors for 10-year cardiovascular disease and all-cause mortality. Models were computed using a one-year landmark analysis. (A) Aggregate global population-attributable fractions. (B) Population-attributable fractions for aggregate risk factors by geographic region. (C) Population-attributable fractions for single risk factors in direct regional comparison.

The distributional assumptions of the models used in the population-attributable fraction estimation were examined graphically (Figure S8). The five modifiable risk factors accounted for an aggregate global CVD population-attributable fraction of 57.2% (95% confidence interval [CI], 52.4 to 62.1) in women and 52.6% (95% CI, 49.0 to 56.1) in men. In comparison, the aggregate global all-cause mortality population-attributable fraction for women was 22.2% (95% CI, 16.8 to 27.5) and for men was 19.1% (95% CI, 14.6, 23.6) (Figure 3).

The CVD population-attributable fractions of aggregate modifiable risk factors for women and men were 64.2% (95% CI, 59.8 to 68.6) and 60.5% (95% CI, 57.2 to 63.9), respectively, in North Africa and the Middle East and 55.5% (95% CI, 50.7 to 60.3) and 50.3% (95% CI, 46.8 to 53.8), respectively, in North America. Aggregate all-cause mortality population-attributable fractions in Asia were 34.3% (95% CI, 29.7 to 38.9) for women and 43.2% (95% CI, 39.8 to 46.6) for men; in Australia were 13.7% (95% CI, 7.1 to 20.3) for women and 2.9% (95% CI, −3.7 to 9.5) for men; and in Western Europe were 15.7% (95% CI, 9.3 to 22.0) for women and 2.1% (95% CI, −4.3 to 8.6) for men (Figure 3).

Population-attributable fractions of the individual 5 modifiable risk factors are shown in Figure 3. The CVD population-attributable fraction for systolic blood pressure was 29.3% (95% CI, 25.4 to 33.2) in women compared to 21.6% (95% CI, 18.7 to 24.5) for men; for non-HDL cholesterol was 15.4% (95% CI, 10.9 to 19.8) for women and 16.6% (95% CI, 12.6 to 20.6) for men; for diabetes was 15.2% (95% CI, 13.3 to 17.1) for women compared to 10.2% (95% CI, 9.2 to 11.2) for men. The CVD population-attributable fraction for smoking and body-mass index in women were 6.7% (95% CI, 5.8 to 7.6) and 7.6% (95% CI, 5.1 to 10.1), respectively, and for men were 10.7% (95% CI, 9.6 to 11.7) and 7.6% (95% CI, 5.6 to 9.7), respectively. The all-cause mortality population-attributable fraction for diabetes was 12.2% (95% CI, 11.1 to 13.3) for women while that for current smoking in men was 14.4% (95% CI 13.3 to 15.4). Population-attributable fractions for CVD and all-cause mortality by modifiable risk factor categories are shown in Tables S20 and S21).

## Discussion

The Global Cardiovascular Risk Consortium harmonized individual-level data from 1,518,028 individuals who participated in 112 cohort studies conducted in 34 countries in North America, Latin America, Western Europe, Eastern Europe and Russia, North Africa and Middle East, sub-Saharan Africa, Asia, and Australia to assess the effect of five modifiable risk factors on incident CVD and all-cause mortality. The study found that the prevalence of 5 modifiable risk factors and the incidence of CVD and all-cause mortality varied across geographic regions worldwide and women had consistently lower event rates than men. The association between individual modifiable risk factors and both incident CVD and all-cause mortality also varied across regions. The five modifiable risk factors accounted in aggregate for a CVD population-attributable fraction of 57.2% in women and 52.6% in men and for an all-cause mortality population-attributable fraction of 22.2% in women and 19.1% in men. Population-attributable fractions varied by geographic region for CVD and all-cause mortality. Elevated systolic blood pressure appeared to be the largest contributor to the population-attributable fraction for CVD in all regions.

The Global Cardiovascular Risk Consortium and other studies^14–16^ confirmed apparent differences in cardiovascular risk factor profile and event rates in women compared to men, irrespective of geographic region. Differences in risk factor load translate into lifetime risk for CVD^17^, but do not necessarily affect other fatal outcomes. Cardiovascular risk factors are known to increase CVD risk differently across various geographic regions.^18,19^ Among them, high blood pressure is associated with up to 13.5% of all deaths annually worldwide, and is considered as the leading risk factor for CVD.^20^ Strict blood pressure control to a systolic blood pressure of less than 120 mmHg has been associated with lower rates of cardiovascular events and all-cause mortality.^21^ Our data corroborate this observation, as, out of the five risk factors studied, systolic blood pressure may offer the greatest potential for CVD prevention. While there is a strong continuous association between non-HDL cholesterol and incident CVD,^22^ we and others^3,23,24^ observed a U-shaped association of non-HDL cholesterol with all-cause mortality. Although very low non-HDL cholesterol concentrations are related to reduced CVD events,^25,26^ some observations point towards higher all-cause mortality rates in individuals with very low levels at least in longer-term follow-up.^27^ In contrast to what was previously reported,^3^ body-mass index and smoking (at least in some parts of the world) were associated with comparatively modest CVD population-attributable fractions in the Global Cardiovascular Risk Consortium populations. This may be related to underlying differences in population characteristics, risk factor definition and prevalence, or methods used to estimate population-attributable fractions.

Case-control studies like INTERHEART may have overestimated the population-attributable fraction of CVD subtypes by attributing 90% of the risk for myocardial infarction to 9 targetable risk factors.^2^ Evidence from 155,722 prospectively studied participants of the PURE study suggested that 71% of CVD cases are attributable to 14 potentially modifiable metabolic and behavioral risk factors, which is more consistent with our findings.^3^ Our study focused on five modifiable risk factors whose strict control could potentially prevent 57.2% of all CVD cases in women and 52.6% in men globally. The varying impact of individual risk factors across different regions could enable ranking and prioritization of risk factor control for public health action within those regions. However, there is substantial scope to characterize CVD risk more completely. Environmental and exposomal factors such as physical activity^18^, alcohol consumption^28^, air pollution^29^, climate and noise^30^, educational level^3^ or psychosocial risk factors including depression^31^ impact CVD risk. Biomarkers^32,33^ and genetic variants most likely would add to CVD risk prediction.

The Global Cardiovascular Risk Consortium analysis differs from other global initiatives that combine different data sources such as registries, population surveys and health system administrative data to produce meta-analytic summaries^34,35^. The Global Cardiovascular Risk Consortium constitutes a large and comprehensive database of harmonized observational individual-level prospectively collected data. This allows for multiple prespecified statistical analyses on large-scale individual-level data. This study relates major modifiable cardiovascular risk factors to incident CVD and all-cause mortality. Inclusion of cohorts with a larger spectrum of follow-up times enables robust sex-specific analyses and the evaluation of differences among geographic regions.

Our study has several limitations. The Global Cardiovascular Risk Consortium database includes cohorts with varying representativeness, data quality and quantity, varying dates for baseline assessment, follow-up time, endpoint definitions and use of clinical interventions. Variation in the adjudication of causes of death or surrogates of non-fatal myocardial infarction is plausible across regions, but analysis using a secondary CVD definition excluding unclassifiable death, unstable angina and coronary revascularization did not change results. Structured harmonization was utilized to reduce variation and sensitivity analyses provided similar results compared to the overall study population. Standardized event rates should rather be interpreted as descriptive measures and not as actual incidences in a population. To overcome bias resulting from deaths from non-cardiovascular diseases present at the time of the baseline examination, analyses were performed excluding the first year of follow-up. Modifiable risk factor information for patients was available from the baseline examination and the impact of changes in exposure over time are not known and the analyses are not corrected for regression dilution bias. Residual confounding cannot be completely excluded. The effects of overweight and obesity may be mediated by hyperlipidemia, hypertension and diabetes.^36^ Models that included body-mass index, systolic blood pressure and diabetes attribute this share of the body-mass index effect to systolic blood pressure and diabetes even if overweight and obesity is the real underlying cause. The definition of current smoking may not capture the entire spectrum and dose of tobacco exposure, and smoking cessation during follow-up might have led to an underestimation of tobacco smoking as a risk factor. It was also assumed that risk factor effects and prevalence within a region are homogeneous; however, intraregional differences might exist. Information on ethnicity is not provided, as definitions differed by cohorts, or collection of the variable was incomplete or not available to a comparable standard. The WHO and United Nations stratification of geographic regions was adapted to accommodate cohort size and representativeness of a geographic region so different categorization of regions may produce different results.

In conclusion, harmonized individual-level data from the Global Cardiovascular Risk Consortium found that 57.2% of incident CVD in women and 52.6% in men, and 22.2% of deaths in women and 19.1% in men may be attributable to five modifiable risk factors. The prevalence and impact of these risk factors on incident CVD and all-cause mortality varies by sex and across geographic regions.

## Supplementary Material

Supp App

## Figures and Tables

**Table 1.** Cohort studies’ sex- and age-standardized baseline characteristics according to geographic region.

	Geographic regions								
	Global	North America	Latin America	Western Europe	Eastern Europe and Russia	North Africa and Middle East	Sub-Saharan Africa	Asia	Australia
**Participants — no.**	1,518,028	65,182	191,244	907,760	51,133	185,608	10,390	59,802	46,909
**Study cohorts — no.**	112	11	10	58	16	5	2	4	6
**Survey year — range** *	1963–2020	1971–2011	1990–2013	1970–2015	1983–2014	1963–2020	2011–2017	1988–2015	1983–2007
**Age at time of baseline examination (IQR) — yr.** **	54.4 (45.2, 63.0)	54.0 (45.0, 63.0)	54.0 (45.0, 63.0)	54.6 (45.5, 63.0)	54.1 (45.5, 63.0)	54.0 (45.0, 62.6)	54.0 (45.0, 63.0)	54.0 (45.0, 63.0)	54.6 (45.5, 63.0)
**Male sex** — %**	45.9	45.9	45.9	45.9	45.9	45.9	45.9	45.9	45.9
**Body-mass index** — kg/m^2^ **(IQR)**	26.4 (23.7, 29.7)	27.2 (24.1, 31.0)	28.2 (25.4, 31.5)	26.1 (23.6, 29.2)	27.2 (24.3, 30.6)	27.0 (24.0, 30.3)	21.0 (19.0, 23.4)	22.8 (20.5, 25.2)	26.4 (23.7, 29.5)
**Systolic blood pressure** — mm Hg	130.0 (118.0, 144.0)	122.0 (111.0, 136.0)	126.7 (118.0, 138.7)	134.0 (122.0, 148.0)	132.0 (120.0, 148.0)	115.0 (105.0, 130.0)	125.0 (113.0, 140.0)	123.5 (112.0, 136.0)	127.0 (116.5, 139.0)
**Diastolic blood pressure** — mm Hg	80.0 (72.0, 87.5)	74.0 (67.0, 81.0)	82.7 (76.7, 90.0)	81.0 (74.0, 89.0)	82.0 (75.0, 91.0)	75.0 (67.5, 80.0)	75.0 (69.0, 83.0)	76.0 (68.0, 84.0)	72.5 (64.5, 80.5)
**Non-HDL cholesterol** — mg/dL	156.9 (128.8, 187.9)	150.0 (123.0, 179.4)	156.2 (131.1, 185.2)	162.8 (134.8, 193.8)	162.4 (135.0, 191.8)	140.1 (115.3, 167.8)	116.0 (77.3, 154.7)	140.0 (117.6, 167.0)	151.2 (124.5, 181.0)
**Current smoking** — %	21.6	22.5	30.8	20.9	29.2	14.2	18.6	23.5	14.3
**Diabetes** — %	8.3	13.0	15.3	4.8	9.0	18.3	2.0	5.1	4.8
**Antihypertensive medications** — %	19.4	27.5	19.3	17.9	28.8	24.7	18.5	11.6	13.7
**Lipid-lowering medications** — %	9.6	8.0	2.3	11.5	8.8	11.6	-	4.4	4.1
**History of CVD** — %	5.6	7.2	3.6	5.6	11.2	5.6	0	6.3	7.2

Percentages are used for binary variables. Percentages and quartiles are based on available cases per variable. Quartiles and percentages per geographic region were computed using direct standardization according to the age and sex distribution of the Global Cardiovascular Risk Consortium dataset. For the standardization the following age groups were considered: age ≤40 years; 40< to ≤45; 45< to ≤50; 55< to ≤60; 65< to ≤70; and >70 years.

* Approximate number of observations according to categorized examination year: 1963–1989: 198,517 (13.1%), 1990–1999: 227,002 (15.0%), 2000–2009: 746,074 (49.1%), 2010–2020: 342,887 (22.8%).

** Similar characteristics for age and sex across geographic regions result from the age and sex standardization.

Non-HDL cholesterol denotes non-high-density lipoprotein cholesterol.

To convert the values for non-HDL cholesterol from milligrams per deciliter (mg/dL) to millimoles per liter (mmol/L), multiply by 0.02586.

**Table 2.** Age-standardized outcomes per 1000 person-years according to geographic region and sex.

	Cardiovascular disease events						All-cause deaths					
	Women			Men			Women			Men		
Region	No. of events	10-year % (95% CI)	Per 1000 person-years (95% CI)	No. of events	10-year % (95% CI)	Per 1000 person-years (95% CI)	No. of events	10-year % (95% CI)	Per 1000 person-years (95% CI)	No. of events	10-year % (95% CI)	Per 1000 person-years (95% CI)
**Global**	30,033	4.0 (4.0, 4.1)	5.2 (5.2, 5.3)	50,563	7.8 (7.7, 7.9)	9.9 (9.8, 10.0)	78,608	6.1 (6.0, 6.2)	9.0 (9.0, 9.1)	98,761	9.3 (9.2, 9.4)	13.4 (13.3, 13.4)
**North America**	4702	7.4 (7.1, 7.8)	10.1 (9.8, 10.4)	5321	13.7 (13.1, 14.2)	16.6 (16.2, 17.1)	8674	7.6 (7.3, 8.0)	16.7 (16.4, 17.0)	8128	11.3 (10.8, 11.8)	21.4 (20.9, 21.8)
**Latin America** *	71	-	2.4 (1.9, 3.1)	89	-	4.1 (3.3, 5.0)	12,488	6.8 (6.6, 7.0)	7.5 (7.4, 7.7)	9733	9.7 (9.5, 10.0)	10.7 (10.4, 10.9)
**Western Europe**	22,212	3.7 (3.6, 3.8)	4.9 (4.8, 4.9)	40,942	7.3 (7.2, 7.5)	9.6 (9.5, 9.7)	42,676	5.6 (5.5, 5.7)	8.9 (8.8, 9.0)	59,447	8.4 (8.3, 8.5)	12.7 (12.6, 12.8)
**Eastern Europe and Russia**	1078	5.7 (5.0, 6.4)	8.7 (7.9, 9.5)	1508	9.9 (8.9, 10.9)	13.5 (12.4, 14.6)	3255	10.1 (9.5, 10.7)	12.7 (12.2, 13.3)	4827	17.9 (17.1, 18.6)	22.1 (21.4, 22.8)
**North Africa and Middle East**	1146	6.4 (5.6, 7.2)	4.0 (3.7, 4.3)	1805	9.4 (8.5, 10.2)	6.8 (6.5, 7.2)	1650	8.1 (7.2, 9.0)	6.1 (5.7, 6.5)	8615	11.9 (11.3, 12.5)	16.2 (15.8, 16.6)
**Sub-Saharan Africa** **	3	-	0.1 (0.0, 0.2)	1	-	0.0 (0.0, 0.2)	431	27.2 (16.0, 36.9)	14.1 (12.7, 15.6)	456	34.6 (25.1, 42.9)	26.7 (24.1, 29.6)
**Asia**	311	2.5 (1.8, 3.2)	2.5 (2.2, 2.9)	353	4.2 (3.0, 5.4)	5.1 (4.5, 5.9)	6399	11.0 (9.9, 12.0)	7.6 (7.1, 8.1)	4751	16.7 (15.1, 18.4)	12.0 (11.2, 13.0)
**Australia**	510	4.9 (4.3, 5.5)	6.0 (5.4, 6.6)	544	9.2 (8.3, 10.1)	10.3 (9.4, 11.4)	3035	4.7 (4.5, 5.0)	5.6 (5.4, 5.9)	2804	7.2 (6.8, 7.6)	8.9 (8.5, 9.3)

Computations were performed using 1,088,670 individuals for cardiovascular disease and 1,419,699 individuals for all-cause mortality. Ten-year percentage of events were estimated using the Kaplan-Meier estimator. Events per person year were estimated using the complete follow-up and a Poisson regression with log-transformed follow-up time as an offset. Direct standardization according to the age distribution of the Global Cardiovascular Risk Consortium dataset was used when computing ten-year percentage of events and events per person years per geographic region. For the standardization the following age groups were considered: age ≤40 years; 40< to ≤45; 45< to ≤50; 55< to ≤60; 65< to ≤70; and >70 years. Confidence interval widths have not been adjusted for multiplicity and should not be used in place of hypothesis testing.

* Since the CVD follow-up in Latin America is shorter than 10 years, it is not possible to obtain an estimate of the 10-year probability of event using the Kaplan-Meier estimator.

** Due to low number of CVD events recorded, the 10-year percentage of events and events per person year were not estimated. CI: Confidence Interval.
