# Supplementary material for: Global Impact of Modifiable Risk Factors on Cardiovascular Disease and Mortality
Source: N Engl J Med. Author manuscript; Available in PMC 2023 Oct 21. (PMC10589462; doi:10.1056/NEJMoa2206916)
Supplement: Supp App [file NIHMS1931665-supplement-Supp_App.pdf]

Supplementary Appendix

Supplementary material to: Magnussen C, Ojeda FM, Leong DP et al. Global Impact of Modifiable Risk Factors on Cardiovascular Disease and Mortality. N Engl J Med. DOI:

This appendix has been provided by the authors to give readers additional information about the work.

Table of Contents

Collaborators ... 1

Acknowledgements ... 2

Sources of funding ... 2

Supplementary Methods ... 3

Study population ... 3

Cardiovascular risk factors and outcome definitions ... 3

Description of longitudinal population-based cohorts ... 3

Description of health examination survey data ... 29

Data harmonization ... 36

Statistical methods ... 37

Supplementary Results ... 40

Supplementary Figures ... 40

Figure S1. Flow chart. ... 40

Figure S2. Sex-specific cumulative incidences for individuals 40 years of age for a) cardiovascular disease and b) all-cause mortality using age as time scale. ... 41

Figure S3. Categorized body-mass index hazard ratios for a) cardiovascular disease and b) all-cause mortality. ... 43

Figure S4. Associations of continuous risk factors with a) cardiovascular disease and b) all-cause mortality allowing for non-linear effects by geographic region ... 45

Figure S5. Associations of continuous risk factors with a) cardiovascular disease and b) all-cause mortality allowing for non-linear effects by geographic region using a two-year landmark analysis. ... 47

Figure S6. Associations of continuous risk factors with a) cardiovascular disease and b) all-cause mortality allowing for non-linear effects by geographic region and adjusting for lipid-lowering medications. ... 49

Figure S7. Associations of risk factors with a) cardiovascular disease and b) all-cause mortality allowing for effects to change with age by geographic region. ... 51

Figure S8. Graphical verification of distributional assumptions of Weibull models used for population-attributable fraction computations. ... 53

Supplementary Tables ... 57

Table S1. Variables of interest. ... 57

Table S2. End point definition. ... 57

Table S3. Background information and representativeness of the broader population affected by cardiovascular disease. ... 58

Table S4. Cohort studies' data availability according to geographic region. ... 59

Table S5. Cohort studies' sex- and age-standardized selected baseline characteristics according to geographic region. 60

Table S6. Cohort studies' baseline characteristics according to geographic region. 61

Table S7. Cohort studies' baseline characteristics according to geographic region and sex. 62

Table S8. Cohort studies' risk factor prevalence according to geographic region and sex. 63

Table S9. Health examination surveys' baseline characteristics by survey. 65

Table S10. Health examination surveys' baseline characteristics according to geographic region. 67

Table S11. Health examination surveys' risk factor prevalence according to geographic region and sex. 68

Table S12. Cohort studies' follow-up information for cardiovascular disease. 70

Table S13a. Risk factor hazard ratios for cardiovascular disease. 73

Table S13b. Risk factor hazard ratios for all-cause mortality. 74

Table S14. Hazard ratios and subdistribution hazard ratios for five risk factors and cardiovascular disease by sex. 75

Table S15a. Risk factor hazard ratios for cardiovascular disease using two-years landmark. 76

Table S15b. Risk factor hazard ratios for all-cause mortality using two-years landmark. 77

Table S16a. Risk factor hazard ratios for cardiovascular disease using only studies with examination year greater or equal than 2000. 78

Table S16b. Risk factor hazard ratios for all-cause mortality using only studies with examination year greater or equal than 2000. 79

Table S17a. Risk factor hazard ratios for cardiovascular disease using only individuals with information on lipid-lowering medications use. 80

Table S17b. Risk factor hazard ratios for all-cause mortality using only individuals with information on lipid-lowering medications use. 81

Table S18. Risk factor hazard ratios for cardiovascular disease. Comparison with a secondary cardiovascular disease end point. 82

Table S19a. Unadjusted risk factor hazard ratios for cardiovascular disease. 84

Table S19b. Unadjusted risk factor hazard ratios for all-cause mortality. 85

Table S20a. Population-attributable fractions for 10-year cardiovascular disease by risk factor categories, geographic region and sex. 86

Table S20b. Population-attributable fractions for 10-year all-cause mortality by risk factor categories, geographic region and sex. 87

Table S21a. Unadjusted population-attributable fractions for 10-year cardiovascular disease by risk factor categories, geographic region and sex. 89

Table S21b. Unadjusted population-attributable fractions for 10-year all-cause mortality by risk factor categories, geographic region and sex. 91

References 93

Collaborators (in alphabetical order)

Fereidoun Azizi (Endocrine Research Center, Research Institute for Endocrine Sciences, Shahid Beheshti University of Medical Sciences, Tehran, Iran), **Alireza Ansari-Moghaddam** (Health Promotion Research Center, Zahedan University of Medical Sciences, Zahedan, Iran), **Stephan Bakker** (Department of Nephrology, University of Groningen and University Medical Center Groningen, The Netherlands), **Fabian J. Brunner** (University Heart & Vascular Center Hamburg, Hamburg, Germany; University Medical Center Hamburg-Eppendorf, Hamburg, Germany; German Centre for Cardiovascular Disease (DZHK), Partner site Hamburg/Kiel/Luebeck, Hamburg, Germany), **Günay Can** (Department of Public Health, Cerrahpaşa Faculty of Medicine, İstanbul University-Cerrahpaşa, İstanbul, Turkey), **Giancarlo Cesana** (Research Centre on Public Health, Department of Medicine and Surgery, University of Milano-Bicocca, Monza, Italy), **William Checkley** (Division of pulmonary and critical care, Johns Hopkins University, Baltimore, MD, USA), **Jean Dallongeville** (Institut Pasteur de Lille, Lille, France), **Abdoulie Faal** (MRC Unit The Gambia @ London School of Hygiene & Tropical Medicine, Banjul, The Gambia), **Mojtaba Farjam** (Noncommunicable Diseases Research Center, Fasa University of Medical Sciences, Fasa, Iran), **Simona Giampaoli** (Department of Cardiovascular, Dysmetabolic and Aging-associated Diseases, Instituto Superiore di Sanità, Rome, Italy), **Graham Giles** (Melbourne, VIC, and Precision Medicine, School of Clinical Sciences at Monash Health, Monash University, Clayton, Australia), **Alina Goßling** (University Heart & Vascular Center Hamburg, Hamburg, Germany; *Population Health Research Department*, University Heart & Vascular Center Hamburg, University Medical Center Hamburg-Eppendorf, Hamburg, Germany), **Tuija Jääskeläinen** (THL-Finnish Institute for Health and Welfare, Helsinki, Finland), **Torben Jørgensen** (Center for Clinical Research and Prevention, Bispebjerg/Frederiksberg Hospital, Copenhagen, Denmark; Department of Public Health, Faculty of Health and Medical Sciences, Copenhagen University, Denmark. Faculty of Medicine, Aalborg University, Denmark), **Bijoy Joseph** (THL-Finnish Institute for Health and Welfare, Helsinki, Finland), **Line Lund Kårhus** (Center for Clinical Research and Prevention, Bispebjerg and Frederiksberg Hospital, Copenhagen, Denmark), **Jens Klotsche** (German Rheumatism Research Centre · Epidemiologic Unit, Berlin, Germany), **Fariborz Mansour-Ghanaei** (Gastrointestinal and Liver Diseases Research Center, Guilan University of Medical Sciences, Rasht, Iran), **Mahmood Moosazadeh** (Gastrointestinal Cancer Research Center, Non-communicable Diseases Institute, Mazandaran University of Medical Sciences, Sari, Iran), **Pablo Kuri-Morales** (Experimental Medicine Research Unit from the School of Medicine, National Autonomous University of Mexico (UNAM), Mexico City; Instituto Tecnológico de Estudios Superiores de Monterrey), **David A. Leon** (Department of Non-Communicable Diseases Epidemiology, London School of Hygiene & Tropical Medicine, London, United Kingdom), **Sarah Lewington** (Clinical Trial Service Unit and Epidemiological Studies Unit, University of Oxford, Oxford, United Kingdom), **Farid Najafi** (Research Center for Environmental Determinants of Health (RCEDH), Health Institute, Kermanshah University of Medical Sciences, Kermanshah, Iran), **Johannes T. Neumann** (University Heart & Vascular Center Hamburg, Hamburg, Germany; University Medical Center Hamburg-Eppendorf, Hamburg, Germany; German Centre for Cardiovascular Disease (DZHK), Partner site Hamburg/Kiel/Luebeck, Hamburg, Germany; Department of Epidemiology and Preventive Medicine, School of Public Health and Preventive Medicine, Monash University, Melbourne, Australia), **Peter M. Nilsson** (Lund University, Department of Clinical Sciences Malmö, Malmö, Sweden, **Torbjørn Omland** (Department of Cardiology, Division of Medicine, Akershus University Hospital, Lørenskog, Norway. K.G. Jebsen Center for Cardiac Biomarkers, Institute of Clinical Medicine, University of Oslo, Oslo, Norway), **Abbas Rezaianzadeh** (Colorectal Research Center, Shiraz University of Medical Sciences, Shiraz, Iran), **Nader Saki** (Hearing Research Center, Clinical Sciences

Research Institute, Ahvaz Jundishapur University of Medical Sciences, Ahvaz, Iran), **Isaac Sekitoleko** (Medical Research Council / Uganda Virus Research Institute, Entebbe, Uganda), **Mohammad Hossein Somi** (Liver and Gastrointestinal Diseases Reaserch Center, Tabriz University of Medical Sciences, Tabriz, Iran), **Poli Mara Spritzer** (Division of Endocrinology, Hospital de Clinicas de Porto Alegre; Department of Physiology, Universidade Federal do Rio Grande do Sul, Brazil), **Roberto Tapia-Conyer** (Experimental Medicine Research Unit from the School of Medicine, National Autonomous University of Mexico (UNAM), Mexico City), **Tetsu Watanabe** (Global Center of Excellence Program Study Group, Yamagata University School of Medicine, Yamagata, Japan), **Jessica Weimann** (University Heart & Vascular Center Hamburg, Hamburg, Germany; *Population Health Research Department*, University Heart & Vascular Center Hamburg, University Medical Center Hamburg-Eppendorf, Hamburg, Germany), **Peter Whincup** (Population Health Research Institute, St George's, University of London, London SW17 0RE), **Tanja Zeller** (University Heart & Vascular Center Hamburg, Hamburg, Germany; *Population Health Research Department*, University Heart & Vascular Center Hamburg, University Medical Center Hamburg-Eppendorf, Hamburg, Germany; German Centre for Cardiovascular Disease (DZHK), Partner site Hamburg/Kiel/Luebeck, Hamburg, Germany)

Global Cardiovascular Risk Consortium Management Group (in alphabetical order)
Jesus Alegre, Larissa Aviles-Santa, Christie M. Ballantyne, Stefan Blankenberg, Modou Jobe, Frank Kee, Kari Kuulasmaa, James de Lemos, Darryl P. Leong, Thiess Lorenz, J. Jaime Miranda, Christina Magnussen, Toshiharu Ninomiya, Francisco M. Ojeda, Arokiasamy Perianayagam, Aletta Schutte, Veikko Salomaa, Sadaf G. Sepanlou, Jonathan E. Shaw, Hugh Tunstall-Pedoe, Raphael Twerenbold, Julia Waibel, Yao Yao, Andreas Ziegler

Global Cardiovascular Risk Consortium Statistical Working Group (in alphabetical order)
Dirk De Bacquer, Alina Goßling, Frank Kee, Kari Kuulasmaa, Thiess Lorenz, Francisco M. Ojeda, Veikko Salomaa, Isaac Sekitoleko, Sadaf G. Sepanlou, Jonathan E. Shaw, Giovanni Veronesi, Jessica Weimann

Acknowledgements

We thank the participants and staff of the cohorts and health examination surveys for their continuing dedication and efforts. The results of this paper are solely the responsibility of the authors. None of the ministries or providing institutions described in the Supplementary Methods are responsible for any content of this research.

Sources of funding

The Global Cardiovascular Risk Consortium and the Center for Population Health Innovation received funding from the German Center for Cardiovascular Research (DZHK). The MORGAM/BiomarCaRE/Global Cardiovascular Risk Consortium Data Center have been sustained by funding from EU projects MORGAM (Biomed, BMH4-CT98-3183), GenomEUtwin (FP5, QLG2-CT-2002-01254), ENGAGE (FP7, HEALTH-F4-2007-201413), CHANCES (FP7, HEALTH-F3-2010-242244), BiomarCaRE (FP7, HEALTH-F2-2011-278913), euCanSHare (Horizon 2020, No. 825903) and AFFECT-EU (Horizon 2020, No. 847770); and Medical Research Council, London (G0601463, No. 80983: Biomarkers in the MORGAM Populations).

Supplementary Methods

Study population

The cohorts for inclusion in the study were selected based on a systematic review of the literature. We evaluated all publications up to June 2020 using PubMed, Web of Science and Google free search as search engines. The search terms included: Population-based, longitudinal, cardi* disease, cerebr* disease, cerebr* infarction, stroke, coronary, heart disease, myocardial infarction, biosamples, biomarkers, lipids, cholesterol, mortality, incidence. In addition, existing collaborations among the study investigators, and the availability of the variables of interest (provided in **Table S1**) for this analysis were used to further identify suitable cohorts.

All datasets used were transferred to the Hamburg Data Center. Grouping of regions was adapted from the World Health Organization (WHO)[1] and United Nations[2] definitions. If the combined cohorts exceeded 5000 individuals from more than one country and event numbers were above 150, geographic regions where further divided following the consensus of the management group and regional representatives. For time-to-event analyses, 83,382 individuals with prevalent CVD (history of myocardial infarction, unstable angina, coronary revascularization, or ischemic or hemorrhagic stroke) at baseline were excluded. Individuals with missing information about prevalent CVD at the baseline examination (n=40,924, 2.5%) were retained and treated as having no CVD at baseline. After further exclusion of individuals with missing follow-up information, 1,088,670 individuals were available for analysis for fatal and non-fatal CVD events and 1,419,699 individuals for all-cause mortality analysis.

Cardiovascular risk factors and outcome definitions

Body-mass index and systolic blood pressure were measured at baseline examination according to the respective cohort's study protocol. Current smoking included regularly (at least 1 cigarette daily) or occasionally (less than 1 cigarette per day) smoking cigarettes, cigars, cigarillos or pipe. Diabetes was defined by medical history, self-report, or newly diagnosed at baseline examination using measures of hyperglycemia, depending on the standard operating procedures of the respective study cohorts. Incident CVD was defined as the first fatal or non-fatal myocardial infarction, unstable angina, coronary revascularization, ischemic or hemorrhagic stroke, and cardiovascular or unclassifiable death. A secondary CVD end point defined as only fatal and non-fatal myocardial infarction, ischemic or hemorrhagic stroke, and cardiovascular death was used for sensitivity analyses.

Description of longitudinal population-based cohorts

North America

Country	Study	Study/cohort full name and short description
USA	ARIC	ARIC is a large-scale, long-term prospective study that investigates associations of established and suspected coronary heart disease risk factors with both atherosclerosis and new CHD events in men and women from four geographically diverse communities. The project has two components: community surveillance of morbidity and mortality; and repeated examinations of a representative cohort of men and women in each community. The community surveillance involves abstracting hospital records and death certificates and investigating out-of-hospital deaths. The representative cohorts include approximately 4,000 persons from each community.<br><br>Informed consent was obtained from all participants. In this analysis 15,028 participants were used with a follow up through 2016.

https://biolincc.nhlbi.nih.gov/studies/aric/

https://aric.cscc.unc.edu/aric9/

USA	CARDIA	The CARDIA study, Coronary Artery Risk Development in Young Adults (CARDIA), is a prospective cohort study that was initiated in 1984 to investigate the evolution of coronary heart disease risk factors during young adulthood. 5,116 black and white women and men, age 18-30 years, were recruited and examined in four urban areas: Birmingham, Alabama; Chicago, Illinois; Minneapolis, Minnesota, and Oakland, California. The initial examination included carefully standardized measurements of major risk factors as well as assessments of psychosocial, dietary, and exercise-related characteristics that might influence them, or that might be independent risk factors. Compared to recent national samples, smoking is less prevalent in CARDIA participants, and weight tends to be greater. Cholesterol levels are representative and somewhat lower blood pressures in CARDIA are probably, at least in part, due to differences in measurement methods. Especially noteworthy among several differences in risk factor levels by demographic subgroup, were a higher body-mass index among black than white women and much higher prevalence of cigarette smoking among persons with no more than a high school education than among those with more education.<br><br>CARDIA has employed a range of strategies to enhance retention, including two contacts per year, multiple tracking methods to locate participants lost-to-follow-up, use of birthday and holiday cards, participant newsletters, examination scheduling accommodations and monetary reimbursements, and a standing committee whose primary purpose has been to continually review retention rates and strategies and identify problems and successes.<br><br>The CARDIA Study received IRB approval and obtained written informed consent of all its participants.<br><br>https://pubmed.ncbi.nlm.nih.gov/3204420/<br>https://www.ncbi.nlm.nih.gov/pmc/articles/PMC5898519/<br>https://biolincc.nhlbi.nih.gov/studies/cardia/
USA	CHS	The Cardiovascular Health Study (CHS; NCT00005133) is a study of risk factors for development and progression of CHD and stroke in people age 65 years and older. The objectives of the Cardiovascular Health Study are to: 1) quantify associations of conventional and hypothesized risk factors with CHD and stroke; 2) assess the associations of non-invasive measures of subclinical disease with the incidence of CHD and stroke; 3) quantify the associations of risk factors with subclinical disease; 4) characterize the natural history of CHD and stroke, and identify factors associated with clinical course; and 5) describe the prevalence and distributions of risk factors, non-invasive measures of subclinical disease, and clinical CHD and stroke. The 5,888 study participants were recruited from four U.S. communities and have undergone extensive clinic examinations for evaluation of markers of subclinical cardiovascular disease. The original cohort totalled 5,201 participants. A new cohort was recruited in 1992. The 687 participants in the new cohort are predominately African-American and were recruited at three of the four field centres. The 2,962 women and 2,239 men were examined yearly from 1989 through 1999. The added minority cohort of 256 men and 431 women was examined from 1992 to 1999. Examination components have included medical history questionnaires, measurement of ankle-brachial index, abdominal and carotid ultrasound studies, echocardiograms, ambulatory electrocardiograms, cerebral magnetic resonance imaging, spirometry, and retinal photographs over the past decade. The most extensive evaluations were at study entry (baseline) and again in 1992-1993 to assess change in subclinical disease measures. CHS has undertaken extensive follow-up for ascertainment of cardiovascular events including incident claudication, myocardial infarction, congestive heart failure, stroke and death. This manuscript was prepared using data obtained from the National Heart, Lung, and Blood Institute (NHLBI) Biologic Specimen and Data Repository Information Coordinating Center (BioLINCC) for the CHS and does not necessarily reflect the opinions or views of the CHS or NHLBI.

		CHS only included participants who were able to give informed consent and gave that consent prior to the start of the study.<br><br>https://biolincc.nhlbi.nih.gov/studies/chs/<br><br>https://chs-nhlbi.org/
USA	DHS	The Dallas Heart Study (DHS) is a multi-ethnic population-based cohort study that was initiated in 2000 with primary goals of improving the diagnosis, prevention, and treatment of heart disease, and reducing racial disparities in CVD. The first phase of the DHS was conducted from 2000 to 2002, with 6101 residents of Dallas County, aged 18 to 65 years completing a detailed medical survey. The cohort was over-sampled for African Americans who represent 50% of the study cohort. Participants over the age of 30 (N=3,557) were invited to provide blood specimens and to undergo comprehensive state-of-the-art imaging studies including cardiac, aortic and abdominal MRI and coronary calcium scanning.<br><br>The second phase of the DHS (DHS 2) occurred in 2007, with a return visit for imaging, biomarker and genetic phenotyping that included the same studies as in phase 1, with the addition of DXA, brain MRI, cognitive testing and other studies. The cohort is currently undergoing a third visit (DHS3, also called the Dallas Hearts and Minds Study). The study was initially supported by the Donald W. Reynolds Foundation and currently is supported by the National Center for Advancing Translational Sciences (NCATS), individual NIH grants, and philanthropic donations. The DHS participants continue to be followed for the development of cardiovascular and metabolic disease through annual surveys and collaboration with hospitals in the Dallas Fort Worth metroplex. All participants gave informed consent before entering into the study, and the study is approved by the UT Southwestern Institutional Review Board.<br><br>https://www.utsouthwestern.edu/research/translational-medicine/doing-research/dallas-heart/
USA	FHS	The objectives of the Framingham Study (FHS; NCT00005121) are to study the incidence and prevalence of cardiovascular disease (CVD) and its risk factors, trends in CVD incidence and its risk factors over time, and familial patterns of CVD and risk factors. Other important objectives include the estimation of incidence rates of disease and description of the natural history of cardiovascular disease, including the sequence of clinical signs and systems that precede the clinically recognizable syndrome and the consequences and course of clinically manifest disease.<br><br>The Framingham Study began in 1948 (FHS-Offspring in 1971, FHS-GEN3/OMNI2/NOS between 2002-2003) under the U.S. Public Health Service and was transferred under the direct operations of the new National Heart Institute, NIH, in 1949. Participants were sampled from Framingham, Massachusetts, including both men and women. This was the first prospective study of cardiovascular disease and identified the concept of risk factors and their joint effects. The study has continued to examine participants every two years and is currently supported by a contract to Boston University from the NHLBI, and from many grants for specialized studies.<br><br>The Framingham Study is a longitudinal investigation of constitutional and environmental factors influencing the development of CVD in men and women. Examination of participants has taken place every two years and the cohort has been followed for morbidity and mortality over that time period. The cardiovascular disease conditions under investigation include coronary heart disease (angina pectoris, myocardial infarction, coronary insufficiency and sudden and non-sudden death), stroke, hypertension, peripheral arterial disease and congestive heart failure.<br><br>All study participants provided written informed consent. For this analysis we included: 12343 participants (from the original cohort, FHS OMNI 1, FHS Gen3/OMNI 2/NOS, and FHS Offspring).https://biolincc.nhlbi.nih.gov/studies/framcohort/<br><br>https://www.framinghamheartstudy.org/

USA	HCHS/SOL	The Hispanic Community Health Study / Study of Latinos (HCHS/SOL) is the most comprehensive study of U.S. Hispanic/Latino health to date. It is an observational and longitudinal epidemiologic study designed to assess the prevalence of specific chronic diseases, their risk and protective factors, and their effect on all-cause and cardiovascular mortality and other health-related events in a cohort of U.S. Hispanics/Latinos. The projected population of interest would be expected to consist of 16,000 adults who self-identified with any of the Hispanic/Latino origin heritage groups represented in the local communities, specifically Central American, Cuban, Puerto Rican, Dominican, Mexican, and South American. Study participants were recruited using a community-based probabilistic sampling in four Field Centers in Miami, Florida, San Diego, California, Chicago, Illinois, and the Bronx area borough of New York. During 2008-2011, 16,415 study participants aged 18-74 years underwent an extensive examination and assessments to assess their baseline health profile. Annual follow-up phone interviews are being conducted since 2009 to determine changes in health and health outcomes of interest. During 2014-2017, a second examination took place, which included an echocardiogram and expanded assessments on women's health, and access to and utilization of health care services. A third examination was initiated in late 2019 and expected to be completed in 2022.<br><br>Study participants have provided signed informed consent at every examination. The study informed consent has been drafted in Spanish and English. A bilingual consent form video and bilingual study staff's assistance have also been made available upon obtaining consent to participate in the study. Every participating academic institution's Institutional Review Board has approved the study. In addition, there is an Observational Studies Monitoring Board that provides oversight of the study activities and compliance with policies related to the performance of research with human participants.<br><br>https://sites.cscc.unc.edu/cscc/projects/HCHS-SOL
USA	JHS	The objectives of the Jackson Heart Study (NCT00005485) are to: 1) investigate the associations of biological, psychosocial, and behavioral factors with the incidence atherosclerotic events and health outcomes in an African American cohort; and 2) increase access to and the participation of African American populations and scientists in biomedical research and professions.<br><br>Participants were enrolled in the study from 2000-2004 from urban and rural areas of the three counties (Hinds, Madison and Rankin) that make up the Jackson MS, metropolitan statistical area (MSA). Participants were enrolled from each of 4 recruitment pools: a random sample component (17%), volunteer component (30%), currently enrolled in the Atherosclerosis Risk in Communities (ARIC) Study (31%), and secondary family members (22%). Recruitment was limited to non-institutionalized adult African Americans 35-84 years old, except in the family cohort where those 21 to 34 years of age were eligible. The final cohort of 5,301 participants includes 6·59% of all African American Jackson MSA residents aged 35-84 (N-76,426, US Census 2000). Data collection at the baseline exam included a medical history, physical examination, blood/urine analyses and interview questions on areas such as: physical activity; stress, coping and spirituality; racism and discrimination; socioeconomic status; and health care access.<br><br>The current release of the Jackson Heart Study includes data collected at the baseline and visit 2 examinations. Jackson is an ongoing study and Eighty-two percent of the surviving JHS participants (N = 4203) completed Exam 2, and projected retention for Exam 3 is 80% (N = 4082). Annual cohort follow-up of the cohort for incident clinical events of interest is ongoing. Written informed consent was obtained from all individuals prior to entering the study.<br><br>https://biolincc.nhlbi.nih.gov/studies/jhs/<br><br>https://www.jacksonheartstudy.org/
USA	MESA	The Multi-Ethnic Study of Atherosclerosis (MESA) (NCT00005487) is a population-based longitudinal study of 6,800 ethnically diverse men and women free of clinical cardiovascular disease at baseline in 2000. MESA is investigating the prevalence, correlates, and progression of subclinical CVD and risk factors that predict progression to clinically overt CVD, and that predict progression of subclinical disease itself, with on-going follow-up of the cohort.

Baseline measurements included coronary calcium using computed tomography; ventricular mass and function using cardiac magnetic resonance imaging; flow-mediated brachial artery endothelial vasodilation, carotid intimal-medial wall thickness, and distensibility of the carotid arteries using ultrasonography; peripheral vascular disease using ankle and brachial blood pressures; electrocardiography; and assessments of microalbuminuria, standard CVD risk factors, sociodemographic factors, life habits, and psychosocial factors. Participants are followed actively for identification and characterization of incident CVD events, including acute myocardial infarction and other coronary heart disease, stroke, peripheral vascular disease, and congestive heart failure; therapeutic interventions for CVD; and mortality. All study participants provided written informed consent.

https://biolincc.nhlbi.nih.gov/studies/mesa/

https://www.mesa-nhlbi.org/

Latin America

Country	Study	Study/cohort full name and short description

Argentina | CESCAS

CESCAS is an observational, prospective cohort study initiated in 2010 with the aim to investigate CVD prevalence and incidence, and CVD risk factors. The study included non-institutionalized mainly urban women and men aged 35 to 74 in Argentina, Chile and Uruguay. Data was collected through home visits by trained interviewers and through clinical visits in health centres including ECG and fasting blood sample analysis. The study also collects data on cardiovascular risk factors as hypertension, dyslipidaemia, smoking, and diabetes. In addition, data on cancer and pharmacological and non-pharmacological treatments was collected.

CESCAS Argentina is based on representative samples from the cities of Bariloche and Marcos Paz. A total of 3990 participants from CESCAS Argentina were included in this work.

This CESCAS study was approved by the institutional review boards in Argentina, Chile, Uruguay and the US, and all subjects provided written informed consent.

This study was supported by the National Heart, Lung, and Blood Institute (NHLBI) grant number HHSN268200900029C.

https://www.ncbi.nlm.nih.gov/pmc/articles/PMC3191438/

Brazil | Passo Fundo

The Passo Fundo cohort is a population-based study with women in pre-, peri- and post-menopause; The objectives of this study, at its core, were to verify the menopausal symptoms among pre- and perimenopausal women, in a city of Southern Brazil. The study has 3 waves so far. The interview occurred in 1995, 2001-2002, and 2010-2011.

The number of participants in the first wave was 298. In the second wave was 358 (it was 55 missing and 119 added); in the third wave, 17 participants died resulting in n= 341.

Follow-ups were conducted with each new wave. The study collected demographic, socioeconomic and lifestyle data. In addition, medical history, physical activity, and additional medical diagnostics, including analyses of blood samples, pelvic ultrasounds, and bone mineral density (only in wave 3) were performed.

Note that in the first wave the data are simpler and there are no lipid or blood glucose tests. The data variables and information are in the Portuguese language in a conventional way and translation can be provided.

Each participant provided written informed consent.

The Passo Fundo Cohort was supported by grants from Conselho Nacional de Desenvolvimento Científico e Tecnológico (CNPq INCT 573747/2008-3), Brazil.

		https://www.ncbi.nlm.nih.gov/pmc/articles/PMC6298002/
Brazil	EpiFloripa	The EpiFloripa Aging is a population-based longitudinal study with 1,705 older adults (60 years or more) living in the municipality of Florianópolis, State of Santa Catarina, Brazil, in 2009/2010 (baseline). The research was conducted with face-to-face interviews, organized into blocks of identification, socioeconomic, mental health, health and life habits, global functionality, falls, physical activity, morbidities, use of health services, use of medications, food, oral health, and violence, evaluated in the first (2009/2010) and in the second wave (2013/2014)<br><br>Before contacting the older adults participating in the first wave of the study (baseline – 2009/2010), we sought to identify deaths, date, and basic cause, based on the Declaration of Death. This search was performed in the database of the Mortality Information System (SIM) of the Ministry of Health, referring to persons aged 60 years or more from Santa Catarina, at two moments: at the beginning and at the end of the interview cycle. The SIM database was obtained by request from the State Health Department of Santa Catarina. After the first search in the SIM database, the older adults not identified as dead were contacted (telephone, e-mail, letter etc.) for confirmation of vital status and data update. The elderly who did not have a contact telephone number were searched directly at the addresses of the data collection of 2009/2010.<br><br>All participants who agreed to participate in the study signed an informed consent form.<br><br>https://www.ncbi.nlm.nih.gov/pmc/articles/PMC5697918/
Chile	CESCAS	CESCAS Chile was founded in 2010 and is based on a representative sample of men and women aged 35 to 74 from the city of Temuco. In this work, 1950 participants from CESCAS Chile were included.<br><br>For more information see CESCAS Argentina. CESCAS Chile was approved by the local institutional ethics board and all participants provided written informed consent.
Costa Rica	CRELES	The Costa Rican Longevity and Healthy Aging Study (CRELES – Costa Rica: Estudio de Longevidad y Envejecimiento Saludable) is a nationally representative longitudinal survey of health and lifecourse experiences of 2,827 Costa Ricans aged 60 and over in 2005. The study is conducted in collaboration with the University of California at Berkeley. The CRELES Retirement cohort is a population-based survey from 2010, which contains 2,798 Costa Rican residents born between 1945 and 1955. Participants recruited are aged between 55 and 60 years. Both cohorts interviewed participants after a 2 year-follow-up and the CRELES retirement cohort also conduced short survey interviews until 2013. Their main goal is to examine determinants of longevity and explore how health and socioeconomic status interact to influence the quality of life of older adults. The survey elicits information about demographics, cognition, family structure, health, healthcare use, job status and history, income, and housing. All participants provided written informed consent at the first interview.<br><br>The original pre-1945 cohort was funded by the Wellcome Trust (grant 072406), and the 1945-1955 Retirement Cohort was funded by the U.S. National Institute on Aging (grant R01AG031716).<br><br>The CRELES study data was accessed through Gateway to Global Aging Data, https://g2aging.org/
Mexico	The Mexico City Diabetes Study	The Mexico City Diabetes Study (MCDS) is a population-based, prospective epidemiological study to investigate the prevalence, incidence and natural history of diabetes and cardiovascular risk factors in a low-income Mexican population. The study was initiated in 1997 and includes adults aged 35 to 64. Pregnant women were excluded from the study. MCDS collected data on socioeconomics, demography, medical history and medical diagnostics according to accepted international standards. Participants have been follow-up regularly since 1997.

		The protocol of MCDS was approved by the Institutional Review Board of the University of Texas Health Science Center at San Antonio and the American British Cowdray Hospital in Mexico City. All the subjects gave informed consent.<br><br>MCDS was supported by research grants from the National Heart, Lung, and Blood Institute (grant R01 HL24799) and by the Consejo Nacional de Ciencia y Tecnologia CONACYT (grants 2092/ M9303, F677-M9407, and 3502-M9607). The Fundacion Mexicana para la Salud has provided administrative support.<br><br>https://pubmed.ncbi.nlm.nih.gov/10391030/<br><br>https://pubmed.ncbi.nlm.nih.gov/15983331/
Mexico	MCPS	The Mexico City Prospective Study (Estudio Mexicano de Cohorte Para Enfermedades Crónicas en una Población Metropolitana) is a blood-based cohort study involving follow-up of 150 000 adults (50 000 men and 100 000 women) who were aged at least 35 years when recruited in 1998–2004. The study resulted from discussions in the early 1990s about how best to measure the changing health effects of tobacco in Mexico. These discussions evolved into a plan to establish a prospective cohort study that could investigate not only the health effects of tobacco but also those of blood lipids and various other risk factors.<br><br>The chief aim of the Mexico City Prospective Study is to assess reliably the associations of established risk factors, and of possible new risks factors, with the common causes of death in Mexico.<br><br>Participants are being followed up indefinitely for cause-specific mortality through Mexican death registries. At 5-year intervals, a reasonably representative sample of at least a few thousand surviving participants will be invited for re-assessment, including the same questions, measurements, and blood collection procedures that were used at baseline. These repeat assessments will be used to help take account of biological variation and random errors in measurements made at baseline. All participants gave informed consent.<br><br>The Mexico City Prospective Study is funded by a core grant from UK Medical Research Council to the MRC Population Health Research Unit at the University of Oxford.<br><br>https://academic.oup.com/ije/article/35/2/243/694920
Mexico	MHAS	The Mexican Health and Aging Study (MHAS) is a national longitudinal study of adults 50 years and older in Mexico.<br><br>The baseline survey, with national and urban/rural representation of adults born in 1951 or earlier, was conducted in 2001 with follow-up interviews in 2003, 2012, 2015, and 2018. A new sample of adults born in 1952-1962 was added in 2012. Similarly, in 2018 a new cohort of adults born between 1963 and 1968 was added to refresh the sample.<br><br>The sample was distributed in all 32 states of the country in urban and rural areas. Households in the six states which account for 40% of all migrants to the U.S. were over-sampled. Follow-up interviews were conducted in 2003, 2012 and 2015.<br><br>Interviews were conducted person-to-person using CAPI (Computer-Assisted Personal Interviewing) by INEGI (interviewers of the Instituto Nacional de Estadistica y Geografia). A next-of-kin interview was completed for those who were part of the panel but died between 2012 and 2015.<br><br>For this analysis only the new subsample of 2012 was used. All participants of that sample provided informed consent.<br><br>https://www.mhasweb.org/Home/StudyDescription.aspx
Peru	CRONICAS	The CRONICAS Cohort Study is a prospective cohort study aimed to compare the prevalence and risk factors of cardiovascular, as well as to compare rate of disease progression to hypertension and diabetes from a disease-free baseline status. The study

		started in 2010 and was conducted in three different settings: Lima, a highly urbanized area, Tumbes, a semiurban region in the north of Peru, and Puno, located in the highlands, contributing with urban and rural areas. Blood samples were collected to measure fasting glucose and blood lipids. The study includes a sex-and age-stratified random sample of women and men aged 35 and older. A total of 6872 subjects were invited to participate in the study. The general response rate at enrolment was 62.9% (4325/6872), and 3601 completed questionnaires. Of them, 3135 had complete clinical measurements and blood samples. The participants were re-assessed in 2012 and 2014, but final follow-up was done between 2017 and 2018.<br><br>Before participation, an informed consent was signed by each participant. The CRONICAS Cohort Study was approved by the institutional review board of the Universidad Peruana Cayetano Heredia and the Johns Hopkins Bloomberg School of Public Health.<br><br>The establishment of the CRONICAS Cohort Study was funded in whole with Federal funds from the United States National Heart, Lung, and Blood Institute, National Institutes of Health, Department of Health and Human Services, under contract no. HHSN268200900033C. The last follow-up was funded by the National Cancer Institute, part of the National Institute of Health (P20CA217231) and the Hamilton Health Sciences Corporation, part of the Population Health Research Institute.<br><br>https://www.ncbi.nlm.nih.gov/pmc/articles/PMC3278488/
Peru	PERU MIGRANT Study	The PERU MIGRANT Study is a prospective cohort study of rural dwellers, urban dwellers and rural-to-urban migrants in Peru initiated in 2007. The aim of the study is to investigate non-communicable diseases and their major common risk factors, namely, raised blood pressure, elevated blood glucose, obesity, low physical activity, unhealthy diet habits, smoking, and alcohol consumption. Blood samples were also collected to measure blood lipids. The study includes women and men aged 30 years and older. A total of 1606 dwellers were invited to participate in the study. The general response rate at enrolment was 73.2% (1176/1606) and the overall response rate at baseline completion was 61.6% (989/1606). Participants were followed-up in 2012 and 2016, and mortality was assessed in 2018.<br><br>Before participation, an informed consent was signed by each participant. The PERU MIGRANT Study protocol was approved by the institutional review board of the Universidad Peruana Cayetano Heredia.<br><br>The establishment of the PERU MIGRANT Study was funded through a Wellcome Trust Master Research Training Fellowship and a Wellcome Trust PhD Studentship to J.J.M. (074833/Z/04/A). The first follow-up evaluation was funded by Universidad Peruana Cayetano Heredia (Fondo Concursable No. 20205071009). The second follow-up evaluation was funded by the National Heart, Lung, and Blood Institute, National Institutes of Health, through the GloCal Health Fellowship Program from the University of California Global Health Institute. Access to mortality information was funded by Wellcome Trust (103994/Z/14/Z). R.M.C-L., A.B-O., J.J.M. and theThe CRONICAS Centre of Excellence in Chronic Diseases were supported by Federal Funds from the United States National Heart, Lung, and Blood Institute, National Institutes of Health, Department of Health and Human Services, under contract No. HHSN268200900033C. L.S. is a Wellcome Trust Senior Clinical Fellow (098504/Z/12/Z), and A.B-O. is a Wellcome Trust Research Training Fellow in Public Health and Tropical Medicine (103994/Z/14/Z).<br><br>https://www.ncbi.nlm.nih.gov/pmc/articles/PMC5837622/
Uruguay	CESCAS	CESCAS Uruguay was founded in 2010 and is based on a representative sample of men and women aged 35 to 74 from the city of Pando-Barros Blancos. In this work, 1584 participants from CESCAS Uruguay were included.<br><br>For more information see CESCAS Argentina. All CESCAS studies were approved by the respective local institutional ethics board and all participants provided written informed consent.

Western Europe

Country	Study	Study/cohort full name and short description
Belgium	BIRNH	The Belgian Interuniversity Research on Nutrition and Health (BIRNH) study was a population-based survey designed to study the association between nutritional patterns, clinical and biochemical characteristics, and cause-specific mortality in men and women. In summary, a random age- and sex-stratified sample was selected from voting lists in 42 out of the 43 administrative districts in Belgium. To achieve a sufficient sample size under circumstances in which little pressure was put on invited eligible subjects, a sample of more than 30,000 persons was selected. The participation rate was 36.5%, resulting in 11,302 subjects taking part in the study (5,949 men and 5,353 women). Baseline data were gathered in the period 1981 to 1984, and included self-administered questionnaires, clinical examinations, and blood sampling. Blood pressure was determined using a random sphygmomanometer; the average of two readings was used in our analyses. A non-fasting blood sample was taken from an antecubital vein in supine position and analysed for serum total cholesterol using commercial reagents (Technicon, Tarrytown, NY, US).<br><br>All participants were followed for at least 10 years for cause-specific mortality, the follow-up being complete in 99% of the participants. Vital status was checked through local community registers and causes of death were ascertained from the family doctor and/or the doctor who completed the death certificate. Where appropriate, more information on the exact cause of death was collected from hospital or medical records. Coding of death causes was done according to the International Classification of Diseases, 9th revision (ICD-9).<br><br>All participants gave informed consent. The study design and methodology was approved by the ethical committees of University Hospital Ghent and the Free University of Brussels.<br><br>https://pubmed.ncbi.nlm.nih.gov/6333125/
Denmark	CCHS	The Copenhagen City Heart Study, is a large prospective cardiovascular population study of 19,000 women and men between 20–93 years old, that was launched in 1975. The source population consisted of about 90,000 adults (≥20 years old) living in a defined area in Copenhagen, Denmark. These were identified through the Copenhagen Population Register using a national registration number. The original purpose of the study was to focus on prevention of coronary heart disease and stroke. During the years many other aspects, such as pulmonary diseases, heart failure or arrhythmia, have been added to the study. CCHS conducted 4 stages of follow-ups, namely, from 1976 to 1978, 1981-1983, 1991-1994 and 2001-2003. End points were ascertained through record linkage with the National Patient Register, the Civil Registration System, and the Cause of Death Registration. All participants gave written informed consent.<br><br>https://clinicaltrials.gov/ct2/show/NCT02993172
Denmark	DanMONICA Study	The DanMONICA cohorts from the Center for Clinical Research and Prevention (the previous Research Center for Prevention and Health (RCPH)) are three prospective population-based cohorts from 11 municipalities from the western part of the suburbs of Copenhagen, Denmark. Random sampling was based on the national population register, stratified by sex and year of birth. Cohort 1 and 3 consists of men and women aged 30-70 years and cohort 2 consists of men and women aged 30-60. Cohort 1 was collected in 1982-1984 (N=4052). Cohort 2 (N=1504) was examined in 1986-1987 and cohort 3 (N=2026) was examined in 1991- 1992. Follow-up is achieved through linkage to the National Cause of Death Register and National Hospital Discharge Register, with end point diagnosis based on MORGAM criteria and validation described elsewhere. The follow-up for the cohorts 1, 2, and 3 were completed to 31st December 2010.<br><br>All participants gave written informed consent.

		http://www.thl.fi/publications/morgam/cohorts/full/denmark/den-gloa.htm
Finland	FINRISK	The FINRISK study is a series of population-based cardiovascular risk factor surveys carried out every five years in five (or six in 2002) districts of Finland, including North Karelia, Northern Savo (former Kuopio), Southwestern Finland, Oulu Province, Lapland province (in 2002 only), and the region of Helsinki and Vantaa. A stratified random sample was drawn for each survey from the national population register; the age-range was 25-74 years. All individuals enrolled in the study received a physical examination, a self-administered questionnaire, and a blood sample was drawn. FINRISK cohorts based on surveys carried out in 1982, 1987, 1992, 1997 and 2002 were used in this analysis. The participation rates can be found in the web address below. During follow-up, the National Hospital Discharge Register, the National Causes of Death Register, and the National Drug Reimbursement Register were used to identify end points. In these analyses, the follow-up extends until 31st December 2010. The Coordinating Ethics Committee of the Helsinki and Uusimaa Hospital District approved the study, which followed the declaration of Helsinki. All subjects gave their informed consent.<br><br>http://www.thl.fi/publications/morgam/cohorts/full/finland/fin-fina.htm
Finland	Health 2000/2011	The Health 2000 Survey was carried out in Finland in 2000-2001. The study population was a two-stage stratified cluster sample representing the adult population living in mainland Finland. The Health 2000 sample comprised 8028 persons aged 30 or older of whom 89% participated in the home health interview and 85% in the comprehensive health examination. The purpose of the survey was to provide an up-to-date account of major public health problems in Finland, their causes and treatment, as well as functional capacity and work ability in the population. For current analyses, we used data from 7,384 individuals, including fatal and non-fatal cardiovascular end points.<br><br>All participants gave written consent.<br><br>https://thl.fi/en/web/thl-biobank/for-researchers/sample-collections/health-2000-and-2011-surveys
Germany	DETECT	The "Diabetes Cardiovascular Risk Evaluation Targets and Essential Data for Commitment of Treatment" (DETECT) trial is a cross-sectional clinical epidemiological study with a prospective-longitudinal component in a nationally representative sample. The baseline study consisted of a nationwide representative sample of doctors with primary care functions (medical practitioners, general practitioners, general internists) in 3,188 primary care offices in Germany. Within this study cohort, a representative partial sample of 7,519 subjects, randomly selected in 1,000 primary care offices, underwent additional laboratory tests and were evaluated for a 5-year time period. The DETECT survey received the approval of the Ethics Committee of the Carl Gustav Carus Medical Faculty at the Technical University of Dresden (AZ EK149092003; Date 16.09.2003) and was registered at clinicaltrials.gov (NCT01076608). The following end points were reported by the physician during the 5-year follow-up: all-cause mortality, mortality of cardiovascular cause, occurrence of a myocardial infarction, and manifestation of CAD as evidenced by the necessity for coronary revascularization by either bypass graft (CABG) surgery or percutaneous coronary intervention (PCI). All information on end points was taken from a standardized assessment form by the primary care physician or the institution in which the patient was previously treated. Further information about causes of death from the death registry was taken into account and all causes of death were confirmed by the physicians who issued the death certificate. Fasting blood samples were collected and shipped by courier within 24 h to the central laboratory at the Medical University of Graz (Austria). Upon arrival, the samples were centrifuged immediately and serum was stored at -20°C until further processing. Clinical chemical parameters as well as cholesterol, triglycerides and lipoprotein (a) [Lp(a)] were determined on a Roche Modular automatic analyser. Lipoproteins (HDL, LDL and VLDL) were determined electrophoretically on the HELENA SAS-3/SAS-4 system. For all parameters, reagents and secondary standards were used as recommended by the manufacturers. All participants signed an informed consent form.

Germany	ESTHER	The ESTHER study (Epidemiologische Studie zu Chancen der Verhütung, Früherkennung und optimierten Therapie chronischer Erkrankungen in der älteren Bevölkerung [German]) is a prospective population-based cohort study in the federal state of Saarland, Germany. In total, 9,949 men and women (age 50-74 years at baseline) were recruited during a routine health check-up between 2000 and 2002. Fatal events were obtained through linkage with the general population registry and cause of death information was collected from death certificates and physicians who issued the certificate. Non-fatal data were collected via questionnaires that were validated by the participant's primary care physician. Follow-up was completed by 31st of December 2010. Approval for the study was granted by the Ethics Committees of the Medical Faculty Heidelberg at the University of Heidelberg and the Physicans' Board of Saarland. All participants provided written informed consent.<br><br>https://www.thl.fi/publications/morgam/cohorts/full/germany/ger-esra.htm
Germany	GHS	The Gutenberg Health Study (GHS) is designed as a community-based, prospective, observational, single-center cohort study in the Rhine-Main area of Western Germany. The sample was drawn randomly from the governmental local registry offices in the city of Mainz and the district of Mainz-Bingen. The sample was stratified 1:1 for sex and residence (urban and rural) and in equal strata for decades of age. Individuals between 35 and 74 years of age were enrolled. Exclusion criteria were insufficient knowledge of the German language and physical or psychological inability to participate in the examinations at the study center. Baseline examination of 15,000 study participants was performed between 2007 and 2012. A 2.5-year follow-up conducted as a telephone interview started in 2009. Since 2012, the 5-year follow-up has been achieved through record linkage, including a second visit at the study centre with extensive medical examination and re-sampling of the biomaterial which is ongoing. GHS obtained follow-up data from computer-supported telephone interviews with subjects as well as a re-examination after 5 years. Overall mortality is obtained through periodic queries with the national registration registry. Cause-specific mortality is obtained through a linkage with the mortality registry Rheinland-Palatinate. GHS validated fatal and non-fatal events through medical files and doctor's letters.<br><br>Written informed consent was obtained by all participants.<br>http://www.gutenberghealthstudy.org/ghs/overview.html?L=1
Germany	KORA	The WHO Multinational Monitoring of Trends and Determinants in Cardiovascular Diseases (MONICA)/ Cooperative Health Research in the Region of Augsburg (KORA) cohorts comprise all respondents from representative sample surveys from the city of Augsburg and the less urban Landkreis Augsburg and Landkreis AichachFriedberg regions in Bavaria, Southern Germany. A list of municipalities and population registers was used as sampling frame for the first and the second stage of two-stage sampling, respectively. The second stage of sampling was stratified by sex and ten-year age groups. The Surveys 1 (S1) (baseline: 1984/85; n=4022), S2 (baseline: 1989/90; n=4940) and S3 (baseline: 1994/1995; n=4856) were carried out as part of the WHO MONICA project and S4 (baseline: 1999-2001; n=4261) was carried out within KORA. Participants were age 25-64 (S1) and 25-74 years (S2-S4) at baseline. Response rates ranged from 79% (S1) to 66% (S4). Follow-up questionnaires were sent to all participants with written consent and current address information who were still alive in 1997-1998 (for S1-S3), in 2001-2002 and in 2008-2009 to obtain information on the occurrence of chronic diseases and risk factors. Coronary events within the study area occurring at ages below 75 years were identified through the MONICA/KORA Augsburg coronary event registry. Non-fatal coronary events which occurred outside the study area and in participants aged ≥75 years and all incident strokes were identified by questionnaires. Mortality follow-up until 2009 was conducted through national death registers and fatal coronary events and fatal strokes were identified through death certificates. Coronary events and strokes were validated by autopsy reports, death certificates or medical records. Information on incident stroke is only available for S3 and S4 in the MORGAM database, therefore, the composite variable on incident CVD is also only available for S3 and S4.

		The study was carried out in accordance with the Declaration of Helsinki as revised in 1996 and approved by the Ethics committee of the "Bayerische Landesärztekammer" Munich.<br><br>http://www.thl.fi/publications/morgam/cohorts/full/germany/ger-auga.htm<br><br>https://www.helmholtz-munich.de/en/epi/cohort/kora
Germany	SHIP	SHIP (Study of Health in Pomerania) is a population-based project conducted in Northeast Germany. The study aims to assess prevalence and incidence of common risk factors, subclinical disorders and clinical diseases and to investigate associations and interactions among them using comprehensive medical assessments. The first SHIP cohort was recruited between 1997 and 2001 and included 4,308 individuals at baseline (SHIP-0, 20-70 years, response 68.8%), 3,300 after five years (SHIP-1) and 2,333 after 11 years (SHIP-2). In parallel to SHIP-2, baseline examinations of a second, independent cohort (SHIP-TREND) were conducted in 4,420 participants (20- 79 years, response 50.3%). For the current analysis only data of SHIP-0 was used. Data were transferred from Greifswald. The harmonization was implemented in Hamburg. SHIP is one of the MORGAM/BiomarCaRE cohort studies and one of the population-based projects with very comprehensive examinations including interviews, cardio-metabolic ultrasound exams, cardiopulmonary exercise tests and whole-body magnetic resonance imaging in a general population setting. In addition to the examination follow-ups, information on fatal and non-fatal disease is collected on a regular basis. However, information of incident CVD is lacking. Mortality follow-ups are conducted semi-annually by record linkage with data bases of the regional population registry. Causes of death are defined from the official death documents provided by regional health authorities. Active follow-ups for non-fatal diseases are performed biannually and by interviews during follow-up examinations every five years. All participants provided written informed consent.<br><br>http://www.medizin.uni-greifswald.de/cm/fv/ship.html<br><br>https://academic.oup.com/ije/article/51/6/e372/6555287
Greece	ATTICA	The ATTICA study is a health and nutrition prospective cohort study of 3,042 men and women participants (aged 18+, 50% men) that was established in 2002 in the metropolitan area of Athens, Greece. The sampling was random, multistage and was based on the age and sex distribution of the province of Attica provided by the National Statistical Service, according to the census of 2001. Only one participant per household was enrolled. The main goal of ATTICA is to record, explore and evaluate the distribution and development in the general Greek population. After the baseline examination, there were two follow-up examinations. In 2006, the ATTICA study's investigators performed the intermediate 5-year follow-up and during 2011-12 they performed the 10-year follow-up.<br><br>The ATTICA study and its study protocol have been approved by the Institutional Ethics Committee and the study was funded by research grants from the Hellenic Cardiological Society. All participants provided written informed consent.<br><br>https://www.maelstrom-research.org/mica/individual-study/attica#/population-ATTICA<br><br>https://link.springer.com/article/10.1007/s00394-022-02831-0
Italy	MATISS Rome Study	The MATISS (Malattie ATerosclerotiche, Istituto Superiore di Sanità) study is an observational longitudinal epidemiologic study designed to assess prevalence of cardiovascular diseases, risk factors and their role on incidence of major cardiovascular events (acute myocardial infarction and stroke), on all-cause mortality and cardiovascular mortality. Participants were recruited and examined in the district of Latina - a rural area located 100 km southeast of Rome - between 1983 and 1987 (N=8,265) and 1993-1996 (N=1,970). Electoral rolls were used as sampling frames in the single-stage sampling, which was stratified by municipality, sex and 5-year age group. The baseline examination was partly carried out as part of the WHO MONICA Project,

		where it was identified with code ITALAT. The other examinations were carried out as part of the MATISS Project using the WHO MONICA methods. Individuals were 20 to 69 years old. From the first screening (1983) to the last screening (1996), municipalities were contacted every five years for information about vital status, emigration, and residency; from 1996 onwards, municipalities were contacted every year. The follow-up procedure covered the Lazio Region. If a person moved out of the Lazio Region, then he/she was lost to follow-up since the date of emigration. Follow-up of non-fatal events was obtained by the hospital discharge registry, events were validated by MONICA diagnostic criteria using clinical records and GPs information. Fatal events were obtained by mortality registry and validated by MONICA diagnostic criteria using death certificates, GPs information and, if available, clinical records. Follow-up was completed to 31st December 2004. Informed consent was obtained from all enrolled participants. The study is part of the CUORE Project of the Istituto Superiore di Sanità and was approved by the Ethic Committee in 2008.<br><br>https://www.thl.fi/publications/morgam/cohorts/full/italy/ita-roma.htm
Italy	Moli-Sani	The cohort of the Moli-sani study was recruited in the Molise region from city hall registries by multistage sampling. First, townships were sampled in major areas by cluster sampling; then, within each township, participants age 35 years or older were selected by simple random sampling. Exclusion criteria were pregnancy at the time of recruitment, inability to comprehend, current multiple trauma or coma, or refusal to sign the informed consent. A total of 24,325 men (47%) and women (53%) were examined at baseline from 2005 to 2010. Participation was 70%. The cohort was followed up for a median of 4.2 years (maximum 6.5 years) by December 2011 (follow-up data was used up to December 2011). Follow-up is achieved through record linkage to national mortality registries and hospital discharge registers, validation of events was achieved through hospital record linkage and doctor's medical records using updated MORGAM criteria. Informed consent was obtained from all participants.<br><br>http://www.moli-sani.org/
Italy	MONICA-Brianza Study	The MONICA-Brianza Cohort Study is a prospective observational study of three cohorts of 25-64 years old residents in Brianza, a highly-industrialized area located between Milan and the Swiss border, Northern Italy. Gender- and ten-year age-stratified samples were randomly drawn in 1986, 1990, and 1993, and cardiovascular risk factors were investigated at baseline following the procedures of the WHO MONICA Project. The overall participation rate was 69%. For all subjects, whole-blood and serum samples were stored in a biobank. The study protocol and follow-up activities received approval by the Monza Hospital Ethical Committee. Study participants were followed up for first coronary or stroke events, fatal and non-fatal, up to the end of 2008, for a median of 15 years. Informed consent was obtained from all enrolled participants.<br><br>http://epimed.uninsubria.eu<br><br>http://biomarcare.eu/the-cohorts-biomarcare/24-population-based-cohorts/42-the-monica-brianza-study
Italy	MONICA Friuli	The Friuli population cohorts were collected as part of the WHO MONICA surveys. The Friuli area covers three provinces of north-east Italy (Friuli-Venezia-Giulia) recruited using a single stage sampling frame from the official Regional Health Roll stratified by Health Unit (combination of municipalities covering 40,000 inhabitants), sex and 5-year age group. Four cohorts were included in these analyses. Follow-up through registry linkage is available until 1998 collected through the framework of the CUORE Project. Non-fatal events and deaths were also followed up through general practitioners and medical records from hospitals. Informed consent was obtained from all enrolled participants.<br><br>https://www.thl.fi/publications/morgam/cohorts/full/italy/ita-fria.htm

Italy	PAMELA Study	The PAMELA study collected a representative sample of N=2,044 men and women aged 25-74 in the region of Brianza, Italy. The city population register was used as a sampling frame for the single state sampling. This sample was stratified by sex and age in 10-year age groups and a random sample of 300 subjects was added to each stratum. The baseline survey was carried out between 1990 and 1993 and after an extension of the follow-up time, it was finalized on the 31st December 2002. The study investigated the following end-points: non-fatal acute myocardial infarction, coronary revascularization, non-fatal stroke events, and death. Informed consent was obtained from all enrolled participants.<br><br>https://www.thl.fi/publications/morgam/cohorts/full/italy/ita-pama.htm
Northern Ireland & France	PRIME	The PRIME (Prospective Epidemiological Study of Myocardial Infarction) study examined the classic and putative cardiovascular risk factors to explain the large difference in heart disease incidence between Ireland and France. The study includes four cohorts of men age 50-59; from Belfast, Northern Ireland (N=2,745) and Lille (N=2,633), Toulouse (N=2,610) and Strasbourg (N=2,612) in France. Baseline examinations took place in 1990-1993 and targeted cohorts which had broadly similar social class structures to the background population, initially sampling from industries and various employment groups, employment groups with more than 10% of their workforce of foreign origin were excluded. Follow-up for 10 years for each participant (Toulouse, Strasbourg and Lille) and for 18 years (Belfast) was achieved through annual follow-up questionnaires with verification against national death registers, medical records, hospital discharge diagnoses. End points were validated by expert medical committee. Informed consent was obtained from all enrolled participants.<br><br>http://www.thl.fi/publications/morgam/cohorts/full/uk/unk-bela.htm
Norway	HUNT	The Trøndelag Health Study (Helseundersøkelsen i Trøndelag [Norwegian]: HUNT) was initiated in the 1980s as a health survey that addressed four main topics: hypertension, diabetes, quality of life, and tuberculosis as well as other lung diseases. Today, the HUNT study includes more than 229,000 adult Norwegian participants from four surveys (HUNT 1 1984-86; HUNT 2 1995-97; HUNT 3 2006-08, and HUNT 4 2017-2019). Every citizen of Nord-Trøndelag County in Norway aged 20 years or older, has been invited to all the surveys for adults. Participants may be linked in families and followed up longitudinally between the surveys and in several national health- and other registers covering the total Norwegian population. In 2018, the two counties Sør-Trøndelag and Nord-Trøndelag were merged into one county: Trøndelag, and from the fall of 2019, HUNT was expanded to include the entire county. Cardiovascular disease end points are followed up through linkage with national registries, including the Norwegian Cardiovascular Disease Registry since 2012. The HUNT Study includes data from questionnaires, interviews, clinical measurements and biological samples (blood and urine). The questionnaires included questions on socioeconomic conditions, health related behaviors, symptoms, illnesses and diseases. For the current analyses, data from 30,557 participants from HUNT 2 was included. All participants provided informed consent.<br><br>https://www.ntnu.edu/hunt
Norway	Tromsø	The Tromsø Study is a prospective repeated population-based health survey of men and women aged 20-97 years in Northern Norway. Specific age groups in the municipality were invited to the different surveys and over time this enabled collection of repeated risk factor measurements in many subjects. The 3rd Tromsø Study survey (Tromsø) was carried out in 1986-87 and those invited were all men in the 20-61 year-old age group, all women in the 20-56 year-old age group, a randomly selected 10% sample from the 12-19 year-old age group (born 1967-1974) and a subsample who were included in a family intervention study. A total of 21,826 participated, 75% of the invited. Of these, data on all men and women age 20-59 years are included in the BiomarCaRE study (N=20,300). A total of 27,158 men and women participated in the subsequent Tromsø 4 survey (participation rate 77%). This analysis comprises two cohorts with N=4,851 (1986-1987 and N=25,705 (1994-1995). The cohorts are being followed up with

		registration of incident myocardial infarction, stroke (ischemic, hemorrhagic, subarachnoid hemorrhage and unclassifiable), diabetes, atrial fibrillation and cause-specific death. Incident events were identified by linkage to the diagnosis registry at the University Hospital of North Norway (the only hospital in the region) and to the National Causes of Death Registry. Validation of hospitalized and out-of-hospital events was performed by an independent end point committee and based on data from hospital and out-of-hospital medical records, autopsy records, and death certificates. Slightly modified World Health Organization MONICA/MORGAM criteria for myocardial infarction and stroke were used. Informed consent was obtained from all enrolled participants.<br><br>https://uit.no/research/tromsostudy
Scotland	SHHEC	The Scottish Heart Health Extended Cohort (SHHEC) consists of two overlapping studies with different age groups but similar protocols. Men and women aged 40-59 were recruited randomly across 22 Scottish districts from 1984-87 for the Scottish Heart Health Study which was not repeated. The Scottish MONICA study sampled men and women aged 25-64 in Edinburgh in 1986 and North Glasgow in 1986, 1989, 1992 (-75) and 1995, as part of the WHO MONICA Project. Of 18,107 in SHHEC, those 15,999 with the most complete risk factor data were entered into the MORGAM study and thence the MORGAM Biomarker Study and BiomarCaRE. Follow-up data extends to the end of 2009 using the Scottish National Health Service Central Register and the Scottish Record Linkage System for mortality and cardiovascular end points. Informed consent was obtained from all enrolled participants.<br><br>https://www.thl.fi/publications/morgam/cohorts/full/uk/unk-sco.htm
Spain	MONICA Catalonia	Two cohorts of the MONICA-Catalonia study, randomly sampled from the general population of nine municipalities of the central area of Catalonia and part of the metropolitan area of Barcelona, were included in this paper. The first stage sampling drew a random sample of municipalities with probability proportional to population size. In the second stage, a ten-year age group stratified random sample of men and women 25-64 years from the selected municipal population registries was drawn. Baseline examinations were carried out in 1986-1988 for cohort 1 (n=2571, response rate 74%). Cohort 2 was collected in 1990-1992 (n=2936, response rate 67%). Participants were mainly from urban areas and the Industrial and services economy, but also included rural settings.<br><br>Follow-up until 1997 for Cohort 1 and until 1999 for Cohort 2 was achieved through follow-up questionnaires and record linkage with MONICA registers, national mortality index register, and hospital discharge registers. Non-fatal strokes were mostly self-reported. Informed consent was obtained from all enrolled participants. The project was funded and approved by the Board of the Institute of Health Studies, Department of Health of Catalonia.<br><br>https://www.thl.fi/publications/morgam/cohorts/full/spain/spa-cata.htm
Sweden	Malmö MDCS	The Malmö Diet and Cancer Study (MDCS) was original initiated to clarify whether a western diet is associated with certain forms of cancer whilst taking other life-style factors into account. Individuals age 45-69 years living in Malmö city, Sweden, were eligible to participate. From 1991-1994, every other participant was invited to also take part in a substudy of the epidemiology of carotid artery disease. A total of 6,103 subjects accepted the invitation and where rescheduled for fasting blood sampling under standardized conditions. Risk factors were estimated on the basis of laboratory tests, baseline examinations, and through the questionnaire administered at the baseline visit. Information regarding smoking habits, physical activity, medical history, and use of medications was based on the self-administered questionnaire. The study was approved by the Ethics Committee at Lund University. Each participant gave his or her written informed consent.

		https://www.malmo-kohorter.lu.se/malmo-cohorts
Sweden	Malmö MPP	The Malmö Preventive Project (MPP) was started in the early 1974 as a screening survey in the middle-aged population of Malmö, the third largest city of Sweden. Subjects born in Malmö and residents of the city were invited for a clinical examination, questionnaire, and blood sampling. In all 22,444 men and 10,902 women participated during the period 1974-1992. Follow-ups have been conducted through linkage with the national registries for fatal or nonfatal coronary events. 44% of the causes of deaths were confirmed through autopsies. Survivors of the original cohort were re-examined between 2002 and 2006 (n=18,240) in the MPP Re-Examination cohort (MPP-RES, attendance rate 72%, https://snd.gu.se/en/catalogue/study/ext0013). All participants who attended the rescreening program gave informed consent.<br>The study was approved by the Regional Ethics Committee, Lund.<br>https://www.malmo-kohorter.lu.se/malmo-cohorts<br>https://www.med.lu.se/malmoe_kost_cancer_och_malmoe_foerebyggande_medicin
Sweden	Northern Sweden MONICA Cohort	The Northern Sweden MONICA cohort was recruited in the Västerbotten and Norrbotten counties. Both counties are sparsely populated areas, half rural and half urban with higher-than-average mortality and unemployment and low socioeconomic status. The cohort was formed by the respondents of representative sample surveys with an age from 24 to 75 years. National population register was used as sampling frame for the single stage sampling which was stratified by sex and 10-year age group. The baseline examinations between 1986 and 1994 were part of the WHO MONICA Project but has since 1999 continued as the Northern Sweden MONICA Study. Recruitment was performed in 1986 (N=1,625), 1990 (N=1,576), 1994 (N=1,893), 1999 (N=1,789), 2004 (N=1,863) and 2009 (N=1,704). The response rate was between 68 and 81%. All participants gave written consent.<br>Everyone in Sweden has a unique personal identification code issued by the National Tax Authority. Events were identified through linking with the local MONICA CHD, Stroke and Diabetes Registers (validated events) and with the National Inpatient Register and the National Cause of Death Register administered by the National Board of Health and Welfare using the personal identification code, and all events, dates and causes of death were obtained until the 31st December 2011.<br>https://www.thl.fi/publications/morgam/cohorts/full/sweden/swe-nswa.htm
Sweden	ULSAM	In the county of Uppsala, Sweden, all men born between 1920 and 1924 were invited to participate at the Uppsala Longitudinal Study of Adult Men (ULSAM) at age 50. The aim of this study, performed from 1970 to 1973 was to identify risk factors for cardiovascular disease. 82% of the invited men participated (N=2,322). The design and selection criteria for the cohort have been described previously. For the current analysis we included N=2,310 men. ULSAM participants were re-examined at age 60, 70, 77, and 82, 88, and 93 with a median follow-up of 30 years. Uppsala University Ethics Committee approved the study and informed written consent was obtained from study participants.<br>http://www.pubcare.uu.se/ulsam/
The Nether-lands	PREVEND	The PREVEND (Prevention of REnal and Vascular ENd stage Disease) study is a general population-based prospective cohort study that was started in 1997 in the city of Groningen, The Netherlands. For this study all the inhabitants of the city of Groningen were asked, in 1997, to complete a brief questionnaire and provide morning urine. Of the 41,000 respondents, 8,600 persons were immediately invited for further examination. The study is still ongoing, but follow-up on CV mortality has been collected and until 31st December 2016, whereas follow-up on non-fatal CV events has been collected until 31th December 2010. Information on cardiovascular morbidity was collected through linkage with the Dutch national registry of hospital discharge diagnosis (PRISMANT). Mortality data was retrieved from the municipal register and primary cause of death was provided by Dutch Central Bureau of Statistics data based

		on death certificate information. The PREVEND study was approved by the Medical Ethics Committee of the University of Groningen (approval number: MEC96/01/022). Informed written consent was obtained from all participants. The study adhered to the ethical principles set by the Declaration of Helsinki.<br><br>The PREVEND study was supported by The Dutch Kidney Foundation which supported the infrastructure of the PREVEND program from 1997 to 2003 (Grant E.033). The University Medical Center Groningen supported the infrastructure from 2003 to 2006. The Netherlands Heart Foundation provided support for studies on HDL metabolism (Grant 2001.005)<br><br>https://umcgresearch.org/w/prevend
The Nether-lands	Rotterdam Study	The Rotterdam Study is a prospective cohort study ongoing since 1990 in the well-defined Ommoord district in the city of Rotterdam in The Netherlands. The study targets cardiovascular, endocrine, hepatic, neurological, ophthalmic, psychiatric, dermatological, otolaryngological, locomotor, and respiratory diseases. The Rotterdam Study initially included 7,983 persons (78% of 10,215 invitees) 55 years of age or older (Rotterdam Study I). In 2000, 3,011 participants (out of 4,472 invitees) who had become 55 years of age or moved into the study district since the start of the study were added to the cohort (Rotterdam Study II). In 2006, a further extension of the cohort was initiated in which 3,932 subjects aged 45–54 years (out of 6,057 invited) were included (Rotterdam Study III). By the end of 2008, the Rotterdam Study therefore comprised 14,926 subjects aged 45 years or over.<br><br>The Rotterdam Study has been approved by the Medical Ethics Committee of the Erasmus MC (registration number MEC 02.1015) and by the Dutch Ministry of Health, Welfare and Sport (Population Screening Act WBO, license number 1071272-159521-PG). The Rotterdam Study Personal Registration Data collection is filed with the Erasmus MC Data Protection Officer under registration number EMC1712001. The Rotterdam Study has been entered into the Netherlands National Trial Register (NTR; www.trialregister.nl) and into the WHO International Clinical Trials Registry Platform (ICTRP; www.who.int/ictrp/network/primary/en/) under shared catalogue number NTR6831. All participants provided written informed consent to participate in the study and to have their information obtained from treating physicians.<br><br>https://link.springer.com/article/10.1007/s10654-020-00640-5
United Kingdom	BRHS	The British Regional Heart Study (BRHS) is a prospective study in middle-aged men (40-59 years) drawn from general practices in 24 British towns (recruited in 1978- 1980). The study was set up to determine the factors responsible for the considerable variation in coronary heart disease, hypertension, and stroke in Great Britain. It also seeks to determine the causes of these conditions in order to provide a rational basis for recommendations towards their prevention. The 20-year re-examination, used for current analyses, took place between 1998 and 2000 when the men were aged 60-79 years.<br><br>https://www.ucl.ac.uk/iehc/research/primary-care-and-population-health/research/brhs
United Kingdom	UK Biobank	The UK biobank is a large-scale prospective study with 502,489 (46%male) participants between 37 and 73 years old, which were recruited in 22 research assessment centers across the UK from 2006 till 2010. The response rate was 5.5%. The UK Biobank aims to create a better understanding, prevention and treatment of a range of different diseases. Besides standardized questionnaires and physical exams, the participants also give biosamples and genetic data. End points are mainly collected through linkage with national datasets, including the death and cancer registry, primary care data and hospital in- and outpatient episodes. The current dataset is followed up through 2018. All participants gave informed consent.<br><br>https://www.ukbiobank.ac.uk/

Eastern Europe and Russia

Country	Study	Study/cohort full name and short description
Eastern Europe	HAPIEE	Health, Alcohol and Psychosocial factors in Eastern Europe (HAPIEE): The HAPIEE study comprises four prospective urban population-based cohorts from Eastern Europe, including Novosibirsk (Russia), Krakow (Poland), Kaunas (Lithuania), and two cities of the Czech Republic. Each cohort recruited a random sample of men and women age 45-69 years at baseline in May 2002 (August 2006 in Lithuania), stratified by sex and 5-year age group. Participants were selected from population registers (electoral roll list in Russia). Participants completed extensive questionnaires, underwent examination in a clinic and provided a blood sample. The sample size (response rates) were 9,360 (61%) in Russia; 10,728 (61%) in Poland; 7,161 (61%) in Lithuania; and 8,857 (55%) in the Czech Republic. Deaths in the cohorts were identified by linkages with national or regional death registers. These datasets used as validation cohort study have only fatal end points. Follow-up is completed to 2011 for the Czech Republic and Lithuania, to 2010 for Russia and to 2009 for Poland. All participants gave written informed consent.<br><br>http://www.ucl.ac.uk/easteurope/hapiee.html
Estonia	EGCUT	Estonian Genome Center of the University of Tartu (EGCUT) – The Estonian Biobank: The Estonian Biobank is a population-based biobank of the Estonian Genome Center of the University of Tartu (EGCUT). The project is conducted in accordance with the Human Genes Research Act of Estonia (www.biobank.ee) and all subjects have been recruited randomly, on a voluntary basis by general practitioners and physicians in hospitals. As of June 2013, the number of individuals is 51,713, which represents about 5% of Estonia's adult population. Phenotyping of subjects (18-103 years of age) was performed by Computer Assisted Personal interview (CAPI), including personal and genealogical data, educational and occupational history, and lifestyle factors. Follow-up of incident fatal and non-fatal coronary heart disease and stroke events of a subset of the cohort is on-going as our database is being linked with the national healthcare registries and regional and central hospital databases. Events are recorded according to the International Classification of Diseases (ICD-10). All subjects provided written informed consent prior to participation and the approval for the study was granted by the Ethics Review Committee on Human Research at the University of Tartu. For current analyses a case-cohort set of 4,971 persons was available.<br><br>https://genomics.ut.ee/en
Kyrgyz Republic	Interepid	The international epidemiological project "Interepid" includes cross-sectional data on the prevalence of major non-communicable diseases and risk factors, and a prospective stage among rural residents of two countries — Russia and Kyrgyz Republic. The study was carried out by one protocol with specifically developed surveys in 2011-2012, with a representative selection of rural inhabitants of the Volzhskiy region of Samarskaya Oblast of Russia (n=1050), and of Chuyskaya Oblast of Kyrgyz Republic (n=1341),that included participants age 20-64 years. In 2019, the vital status of respondents was reassessed (7 years after the initial examination), and end points were collected, including all-cause and cardiovascular deaths. Informed consent status is unknown.<br><br>https://pubmed.ncbi.nlm.nih.gov/32870173/<br><br>https://russjcardiol.elpub.ru/jour/article/view/4999?locale=en_US
Lithuania	Kaunas	Epidemiological studies of heart disease in Kaunas, event registration and population surveys, were started in 1972 as part of the myocardial infarction register and the Kaunas-Rotterdam Intervention study (KRIS) coordinated by WHO. The latter study included only men. In Kaunas, the KRIS study continues as a cohort study. The Kaunas-MONICA study was the first to include both sexes in risk factor surveys, and it also initiated stroke registration.

		The main results of Kaunas-MONICA have been used to prepare strategies for health promotion and prevention of non-communicable diseases at local and national levels. Since 1994 mortality rates from the main cardiovascular diseases have declined in Lithuania. For current analyses, the MONICA cohorts from 1983-85, 1986-1987, and 1992-1993 were used. Follow-up is completed to 31st December 2013. Non-fatal strokes and cardiovascular events were followed up only up to age 65.<br><br>https://pubmed.ncbi.nlm.nih.gov/25479610/
Poland	Krakow	The Krakow study is based on residents age 25–64 of the south-eastern rural province of Tarnobrzeg Voivodship, Poland. Tarnobrzeg Voivodship was chosen for POLMONICA Krakow to contrast its rural population and health care with that of Warsaw. The Project also promoted cardiovascular disease prevention. However, the information on incident cardiovascular disease is absent in the Krakow study. Additional survey data was used locally and for the Poland and US Collaborative Study on Cardiopulmonary Epidemiology. MONICA monitored risk factors, medical care and trends in coronary heart disease mortality (which increased up to, and decreased after, 1992) during major political, economic and social changes. Three cohorts, from years 1983-84, 1987-88 and 1992-93 were used for the current analysis. Follow-up, completed to 31st December 1998, covers fatal cases only.
Poland	Warsaw	The Warsaw study includes residents age 25–64 of the two districts of the capital city of Warsaw east of the Vistula. These districts are partly industrial and partly residential and home to hospitals, banks, governmental offices and universities. Poland's changing economy has affected the living conditions and behaviour of the population. In 1989/90 the free market produced mixed benefits: loss of State social support, high inflation, high unemployment, but greater access to food products previously found only in the western markets. Risk-factor profiles have changed. Cardiovascular disease mortality, previously rising, began to decrease from 1991. The total population in 1991 was 494,000. In this analysis two cohorts were included with N=2,239 and N=2,550, respectively. Fatal events were followed up till 31st December 1998 and non-fatal events till 31st December 1994 with upper age limit of 64 years. All participants provided informed consent.<br><br>https://thl.fi/publications/morgam/cohorts/full/poland/pol-wara.htm
Russia	Novosibirsk Study	For the Novosibirsk Study residents age 25–64 of the city of Novosibirsk, central West Siberia, the industrial and scientific centre of Siberia, were included. Coronary heart disease and stroke morbidity and mortality rates are high in men and women in Novosibirsk. The total population in 1991 was 482,000 (Novosibirsk Control, NOCb) and 160,000 (Novosibirsk Intervention). Population surveys followed the MONICA protocol but with some additional items added. In this analysis we used four cohorts: cohort 1 (Round 1) with N=3,065, cohort 2 and 3 (Round 1) with 6,357, and cohort 4 (Round 1) with 1,546 individuals. The high cardiovascular mortality in Novosibirsk increased dramatically at the beginning of the 1990s but declined modestly after 1994. The follow-up is completed to 31st December 1998, with upper age limit of 64 years.<br><br>https://thl.fi/publications/morgam/cohorts/full/russia/rus-nova.htm
Russia	Interepid	The international epidemiological project "Interepid" includes cross-sectional data on the prevalence of major non-communicable diseases and risk factors, and a prospective stage among rural residents of two countries — Russia and Kyrgyz Republic. The study was carried out by one protocol with specifically developed surveys in 2011-2012, with a representative selection of rural inhabitants of the Volzhskiy region of Samarskaya Oblast of Russia (n=1050), and of Chuyskaya Oblast of Kyrgyz Republic (n=1341), age 20-64 years. In 2019, the vital status of respondents was reassessed (7 years after the initial examination), and end points were collected, including all-cause and cardiovascular deaths. Informed consent status is unknown.<br><br>https://russjcardiol.elpub.ru/jour/article/view/4999?locale=en_US
Turkey	TARFS	The Turkish Adult Risk Factor Study was established in 1990 and expanded in scope through successive waves. The aim of the study was to determine prevalence of cardiovascular risk

factors and coronary heart disease in a random sample of Turkish adults. Information on the cause of death was obtained from first-degree relatives and/or health personnel of a local health office. Information collected in survivors was based on medical history and physical examinations of the cardiovascular system and Minnesota coding of resting electrocardiograms.

TARFS did not collect information on baseline stroke. It is therefore, assumed that it was an exclusion criterion and is not present in the study population.

https://pubmed.ncbi.nlm.nih.gov/23917000/

Sub-Saharan Africa

Country	Study	Study/cohort full name and short description
Gambia	KWLS	The Kiang West Longitudinal Study was established in 2004 and includes 14.846 participants from 36 villages in the Kiang West district of The Gambia. The study collects data on anthropometric measures, blood pressure and diet and lifestyle factors. It also includes urine and blood samples of each participant. Besides the comprehensive demographic surveillance, the study also follows up on hospitalization and health issues, health phenotypes and nutritional data, and links medical records for all participants.<br><br>All studies and data collections in Kiang West are presented to and approved by the MRC Unit The Gambia Scientific Committee (SCC) and joint Gambian Government/MRC Unit The Gambia Ethics committee, which is overseen by the ethics board of the London School of Hygiene and Tropical Medicine (LSHTM). For research studies, all participants and/or legal guardians provide written, informed consent.<br><br>We thank all funders who have supported individual researchers and studies, as well as the infrastructure and research conducted out of MRC Keneba; major funding was received from the UK Medical Research Council (MRC) and the UK Department for International Development (DFID), under the MRC/DFID Concordat agreement (current grants are MC-A760-5QX00, U105960371 and U123261351)<br><br>https://www.ncbi.nlm.nih.gov/pmc/articles/PMC5837564/
Uganda	The General Cohort Study	The General Population Cohort (GPC) was set up in 1989 to examine trends in HIV prevalence and incidence, and their determinants in rural south-western Uganda. Since 2013, the cohort also includes data on non-communicable diseases (NCDs) to address the limited data on the burden and risk factors for NCDs in sub-Saharan Africa. The cohort comprises all residents (52% age ≥13years, men and women in equal proportions) within one-half of a rural sub-county, residing in scattered houses, and largely farmers of three major ethnic groups. Data collected through annual surveys include: mapping for spatial analysis and participant location; census for individual socio-demographic and household socioeconomic status assessment; and a medical survey for health, lifestyle and biophysical and blood measurements to ascertain disease outcomes and risk factors for selected participants.<br><br>Prior to inclusion, all participants gave written informed consent. For participants below 18 years, parental or guardian consent was obtained following Uganda National Council of Science and Technology (UNCST) guidelines. All study procedures including material transfer agreements are approved annually by the Uganda Virus Research Institute Science and Ethics Committee and the UNCST.<br><br>This work was supported by the Medical Research Council UK core funds through all the years, by a Wellcome Trust grant 2009–11 ‘Improving access and quality of data from a longitudinal HIV cohort in Uganda’ and [grant number MSU-G0901213] for the NCD (GPC round 22) by support from Wellcome Trust Sanger Institute for genomic studies.<br><br>https://www.ncbi.nlm.nih.gov/pmc/articles/PMC3600628/

Middle East

Country	Study	Study/cohort full name and short description
Iran	GCS	The Golestan Cohort Study (GCS) is a long-term population-based prospective study in Iran. The primary purpose was to study risk factors of esophageal cancer in a high-risk area in the north-eastern Iran. This population-based cohort enrolled 50045 men and women aged 40-75 years, without history of upper gastrointestinal cancers, from Gonbad City (n=10032) and 326 villages (n=40013) in Golestan Province, between 2004 and 2008. The design and objectives of the GCS have been described previously. In the main phase of the study (enrollment phase), biomarkers were not measured. In the first repeated measurement phase (2010-2012), a random sample of the participants (n=11400; 80% rural and 20% urban participants) were assessed again. Smoking, alcohol use, blood pressure, BMI, drug history, and history of chronic diseases were re-evaluated. In addition, fasting blood sugar, triglycerides, total cholesterol, low-density lipoprotein cholesterol, high-density lipoprotein cholesterol, and creatinine were measured. All participants are being followed up actively every 12 months.<br><br>In case of a reported death, the follow-up team collected all hospital documents, such as physician notes, electrocardiography reports, laboratory test results, and radiologic documents from all medical centers in Golestan Province and the neighboring provinces. Causes of death were determined based on ICD-10 codes.<br><br>The study protocol and the written informed consent used for this study were approved by the ethical review committees of Digestive Disease Research Centre, Tehran University of Medical Sciences (DDRC), International Agency for Research on Cancer (IARC), and National Cancer Institute (NCI). In June 2008, the accrual goal of 50 000 subjects was reached and enrolment was closed.<br><br>GCS was funded by Tehran University/Medical Sciences (81/15 to R.M., Principal Investigator); Cambridge University (Cancer Research UK, C20/A5860) the intramural research program of the NCI, National Institutes of Health; and the IARC.<br><br>https://www.ncbi.nlm.nih.gov/pmc/articles/PMC3709199/
Iran	PERSIAN Cohort	The Prospective Epidemiological Research Studies in IrAN (PERSIAN) is a nationwide cohort study launched in the year 2014 in an attempt to encourage research in the fields of medicine, epidemiology, health, and nutrition. PERSIAN also includes PERSIAN Birth Cohort, the PERSIAN Youth Cohort, and the PERSIAN Elderly Cohort, but only the original cohort study was used for this analysis. This cohort recruited around 170.000 men and women between 35-70 years of age from 17 regions in Iran. Among the aims are to determine patters of causes of death within these regions as well as research on incidence of non-communicable diseases and their prevention, and the relationship between risk factors and these diseases in different regions, cohorts and ethnic groups. Additionally, the PERSIAN cohort study aims to become the largest biobank in Iran that includes genetic scientific research and biomarker analysis. The PERSIAN investigators plan to follow up participants for at least 15 years after enrollment. During this time, participants will receive annual phone calls through which follow-up questionnaires will be completed regarding the occurrence of death or the incidence of any medical events, hospitalizations, or diagnostic/therapeutic care. If a participant has expired or has been diagnosed with a major NCD, investigators will follow the phone call with a house/hospital visit to perform a more thorough follow-up and to collect copies of pertinent medical documents for further evaluation and recording. If needed, medical/physical examinations are performed to formulate a diagnosis. A verbal autopsy form validated in the Iranian population is also completed in the event of death. Two trained internists review the medical documents to determine the final diagnosis of disease or cause of death. If a consensus is not reached, a

		third internist performs a final review of the documents to reach a decision. The same follow-up procedures are followed in the case of self-reported incidence of events.<br><br>Upon arrival at the cohort center, written informed consent is obtained from each participant.<br><br>https://www.ncbi.nlm.nih.gov/pmc/articles/PMC6279089/
Iran	PCS	The Pars Cohort Study (PCS) is a 10-year cohort study aiming to investigate the burden and the major risk factors on non-communicable diseases, and to establish a setting to launch interventions for prevention of these diseases and controlling their risk factors. The participants were recruited between 2012 and 2014 and are from the district of Valashahr. The 9264 participants included, aged 40-75 years, represent both healthy and disease populations of the south of Iran. All participants were actively followed-up for 12 months and then contacted annually for long-term follow-up for at least 10 years. PCS was developed by the joint collaboration of research teams from Non-Communicable Disease Research Center (NCDRC) in Shiraz University of Medical Sciences (SUMS) and Digestive Diseases Research Institute (DDRI) in Tehran University of Medical Sciences. The study protocol and the informed consent used for this investigation were approved by the ethical review committees of DDRI and the SUMS. The informed consent form was completed and signed in the presence of other visitors (third party). These forms and the questionnaires are kept in a secure and safe place. The questionnaires are also available with the online version of the article. The project was operated by a 13-member team in Pars Cohort Study Center (PCSC), including general practitioners, nurses, and nutritionists to work on the food frequency questionnaire (FFQ), lab technicians, data entry team, and receptionists in Valashahr and the nearby villages, in southern Fars Province starting in fall, 2012.<br><br>This work was funded by Shiraz University of Medical Sciences, Grant Number [910210]. All procedures performed in this study involving human participants were in accordance with the ethical standards of the institutional research committees of both Shiraz University of Medical Sciences and Tehran University of Medical Sciences and with the 1964 Helsinki declaration and its later amendments or comparable ethical standards. Informed consent was obtained from all individual participants included in this study.<br><br>https://pubmed.ncbi.nlm.nih.gov/27349480/
Iran	TLGS	The Tehran Lipid and Glucose Study (TLGS), as the first and longest running cohort of its sort in Iran and the middle east, was initiated in 1999-2001 on a population in district no. 13 of Tehran. The purpose of the study was to determine the prevalence and incidence of risk factors for atherosclerosis among the general urban population of Tehran. It also includes a long-term integrated community-based program for prevention of non-communicable disorders by development of a healthy lifestyle. Totally, 15005 individuals, aged ≥ 3 years, participated in the first examination; re-examinations were conducted in a triennial manner and 3550 individuals were added in the second examination (2002-2005). All participants were also followed up annually and asked about any medical event leading to hospitalization or death. A part of participants was assigned to an educational program for lifestyle modification. The study is still ongoing. Besides the triennial re-examinations during in-person visits, all participants were followed up annually by telephone call to them or their family and asked about any medical event leading to hospitalization during the past year. In case of positive responses, related data were collected by a trained physician using hospital records or, if needed, a home visit. Moreover, in the case of mortality outside the hospital, data were collected from the death certificate, the report of forensic medicine and if needed a verbal autopsy from witnesses. All documents collected were reviewed by an adjudication committee and the final diagnosis was recorded, using a predefined coding protocol. The committee consisted of the physician who collected the data, an internist, an epidemiologist, a cardiologist, an endocrinologist, and other experts invited as needed. Response rate for follow-up was around 80-90 percent for cardiovascular events.<br><br>Written informed consent was obtained from all subjects and the study was approved by the ethical committee of Research Institute for Endocrine Sciences.

		https://www.ncbi.nlm.nih.gov/pmc/articles/PMC6289309/<br>https://pubmed.ncbi.nlm.nih.gov/30584434/
Israel	IIHD	The Israeli Ischaemic Heart Disease (IIHD) prospective study was planned to facilitate an approximate representation of mostly migrants from over 20 countries from 5 major areas in 3 continents, Eastern Europe, Central Europe Balkan countries, North African Arab states and Western Asian Mediterranean countries of origin. Diverse sampling rates assured resemblance to ethnic origins of males at ages 40 years through the 65-yr retirement age at the time recruitment began. Over 10,000 male civil servants and municipal employees underwent an extensive clinical, biochemical. Anthropometric, sociodemographic and psychosocial evaluation in the surveys in 1963, 1965 and 1968. Information on use of antihypertensive medications was collected for the 1965 but not in the 1963 survey. Since this information is included as a covariate in the regression models and the 1963 survey is being used as the baseline, those individuals on antihypertensive medications or having no such information available according to the 1965 survey, were excluded from the analyses. They belonged to either government or municipal service in the areas of Tel Aviv, Haifa and Jerusalem. Follow-up for mortality was undertaken in1970 and periodically updated until 2019, primarily based on the national Birth and Mortality Registers. All cancer diagnoses through 2018 were obtained from the National Cancer Registry. Prevalence of Dementia was assessed at 1999/2000 for the remaining 2000 survivors. The original recruitment of patients took place before the establishment of current ethics framework and was arranged through the Israeli Civil Service Commission. Results judged as necessitating added or revised/improved treatment were reported in detail to participants' family physicians. Later end point event assessment was obtained under Helsinki Committee approval or Tel Aviv University Ethics Committee, as appropriate. Informed consent status is unknown. Prime scientific interest, as the study reaches its 60th year of activities in diverse directions, have focused on identified and estimating the role and magnitude of risk factors for the incidence of coronary heart disease, hypertension, subsequently fatal stroke, diagnoses of cancer in specific sites and overall, and survivors' dementia.<br>https://pubmed.ncbi.nlm.nih.gov/8324774/<br>https://pubmed.ncbi.nlm.nih.gov/15308782/

Asia

Country	Study	Study/cohort full name and short description
China	CHARLS	The China Health and Retirement Longitudinal Study (CHARLS) is a nationally representative longitudinal survey that aims to collect a high-quality sample of Chinese residents ages 45 and older to serve the needs of scientific research on the elderly. The CHARLS recruitment was performed from 2011 to 2012 and included 17,707 participants that were collected from 150 counties and 450 villages/resident committees in 28 provinces. Individuals are followed up every two years via surveys. All data will be made public one year after the end of data collection.<br>https://charls.pku.edu.cn/en/
China	CLHLS	The Chinese Longitudinal Healthy Longevity Survey (CLHLS) is a prospective cohort study investigating healthy aging and longevity among older population (>60 years). CLHLS collected a random nationwide sample from half of China's counties and 23 out of 31 provinces, covering about 85% of China's population. Since 1998, every 2-3 years a new wave of the survey has been conducted. To reduce attrition in numbers from death and loss to follow-up, new 'make-up' participants are enrolled during each follow-up wave.<br>Among the randomly selected survey sites of counties/city districts, CLHLS selected eight longevity areas (counties/cities), where the proportion of centenarians are exceptionally high, to conduct in-depth study with 3470, 2860, 2639 and 2786 elderly participants in the CLHLS 2008-2009, 2011-2012, 2014 and 2017-2018 waves. The CLHLS in-depth study in the

		8 longevity areas conducted face-to-face interviews using the same questionnaire as that used in all other study sites plus home-based basic health exams by medical doctors/nurses and blood/urine sample collections from the participants, and biomarkers lab tests and analyses. Follow-ups were conducted to assess different incidences and fatal events were verified through questionnaires with close relatives and treating doctors or nurses.<br><br>All participants gave informed consent.<br><br>CLHLS was approved by the Biomedical Ethics Committee, Peking University (IRB00001052–13074). All participants provided written informed consent prior to participation.<br><br>CLHLS was supported by the Healthy Aging Consortium of the China Cohort Consortium; grants from National Key R&D Program of China [grant number: 2018YFC2000400] and the Natural Science Foundation of China [grant numbers: 81903392, 81941021, 72061137004]<br><br>https://www.icpsr.umich.edu/web/NACDA/studies/36692
Japan	Hisayama	The Hisayama Study is a population-based prospective cohort study that has been conducted in the town of Hisayama, Japan since 1961 and is still ongoing. The town of Hisayama is located in a suburb of the Fukuoka metropolitan area in Kyushu, Japan. This study is designed to evaluate the risk factors for lifestyle-related diseases, such as stroke, coronary heart disease, hypertension, diabetes, and dementia, in a general Japanese population.<br><br>For this collaboration the individual participant data from the cohort in 1988-2012, which includes a total of 2742 residents aged ≥40 years was sent. 80.9% of the total population in this age group were followed up annually prospectively for 24 years, from December 1988 to November 2012, via surveys. All available information about potential CVD events and deaths among the study participants was collected and reviewed by physician members of the study to determine the occurrence of CVD events or cause of death under the standardized diagnostic criteria throughout the study period. Furthermore, if consent for an autopsy was given, autopsy findings were used to adjudicate the underlying cause of death and confirm the existence of CVD (stroke lesions, myocardial necrosis, and atherosclerotic lesions in coronary, carotid, cerebral, and other major arteries) and to classify subtypes of stroke.<br><br>The ethics committee of Kyushu University approved this study, participants provided written informed consent, and the procedures followed were in accordance with national guidelines.<br><br>https://pubmed.ncbi.nlm.nih.gov/30298863/<br>https://pubmed.ncbi.nlm.nih.gov/23902756/
Japan	Yamagata	The Yamagata Study is a community-based study and recruited its participants older than 40 years between 2009 and 2015, who received an annual health check in the Yamagata Prefecture. The aim of Yamagata Study is to elucidate the pathology of risk genes targeting 5 major cancers (lung cancer, stomach cancer, colon cancer, liver cancer (hepatitis), breast cancer), stroke, acute myocardial infarction, hypertension, renal failure, and diabetes, and further to identify drug targets. Hypertension at baseline was defined as a systolic blood pressure ≥140 mm Hg or diastolic blood pressure ≥90 mm Hg, or the use of antihypertensive medications. Subjects with a baseline body-mass index ≥25.0 kg/m2 were categorized as obese. Presence of diabetes was defined as a plasma glucose level ≥126 mg/dL, hemoglobin A1c ≥6.5% (Japanese Diabetes Society value), or the use of antidiabetic medications. Incidence was followed up through the linkage with the Yamagata Stroke and Acute Myocardial Infarction (AMI) registries and the Yamagata Society in Treatment for Cerebral Stroke (YSTCS). Fatal events were collected through ICD-10 coding from causes of death of death certificates.<br><br>This analysis included 20,971 subjects that were followed up until end of 2016. All subjects provided written informed consent prior to enrolment.

The Yamagata study is supported in part by a Grant in-Aid from the 21st Century Center of Excellence (COE) and Global COE program of the Japan Society for the Promotion of Science.

https://www.ncbi.nlm.nih.gov/pmc/articles/PMC7064551/

Australia

Country	Study	Study/cohort full name and short description
Australia	AusDiab	The baseline study conducted in 1999–2000 provided benchmark national data on the prevalence (or number of people) of diabetes, obesity, hypertension, and kidney disease in Australia. The second phase of AusDiab, completed in December 2005, was a five-year follow-up of the people who participated in the baseline survey. A twelve-year follow-up was completed in 2012. AusDiab is a national population-based longitudinal study established to examine the prevalence of diabetes and related risk factors in Australia. Using a stratified cluster sampling method, it enrolled 11,247 adults (aged 25 years and above) from 42 randomly selected census collector districts across Australia between May 1999 and December 2000. Over 85% of the participants were from an Australian, New Zealand or British background. The baseline survey consisted of an initial household interview and was followed by biomedical exams. At baseline and follow-ups, anthropometric measures, and fasting blood samples were collected, and participants completed interviewer-administered questionnaires on health and lifestyle factors. Participants underwent a standard 75 g oral glucose tolerance test and T2D was classified as fasting plasma glucose ≥7.0 mmol/L, 2 h plasma glucose ≥ 11.1 mmol/L or current treatment with insulin or oral hypoglycemic agents.<br><br>Informed consent was obtained from all participants.<br><br>https://www.baker.edu.au/ausdiab/
Australia	Dubbo	The Dubbo Study is an ongoing prospective study of a cohort of elderly people first examined in 1988–1989 in Dubbo, New South Wales. It aims to identify predictors of mortality, hospitalization and placement in long-term care, with special focus on risk factors for cardiovascular disease. The study population were non-institutionalized subjects, comprising 1237 males and 1568 females aged 60 years or older. he goals of the Dubbo Study are to identify predictors of mortality, hospitalization and placement in long-term care, while specific aims are the study of risk factors for chronic diseases and disability. This report describes the study rationale, reasons for selecting Dubbo as the study site, methods and measures, participation rates and demography. The target population included all non-institutionalised subjects 60 years and over, domiciled in Dubbo. The attendance rate for an extensive medical and sociological baseline assessment over a 13 months' period was 73% for both sexes.<br><br>All participants gave informed, written consent.<br><br>https://pubmed.ncbi.nlm.nih.gov/2291727/
Australia	MCCS	The Melbourne Collaborative Cohort Study is a longitudinal study established in the 1990s by Cancer Council Victoria to investigate prospectively the role of diet and other lifestyle factors in cancer. Between 1990 and 1994, 41,500 people (24,500 women and 17,000 men) aged 40 to 69 were recruited into the study. Approximately one quarter of participants were southern European migrants to Australia, who were deliberately over-sampled to extend the range of data on lifestyle exposures and to increase genetic variation. At baseline, lifestyle exposure information, including dietary intake, was collected in a face-to-face interview. Physical measurements and blood pressure were also taken. A sample of blood was drawn and stored for analysis of DNA and other molecules of interest. Follow-up was conducted by mailed questionnaire and telephone to update lifestyle exposures and self-reports of non-cancer, non-fatal health events at 3 to 4 years after baseline. During 2003-2006, approximately 27,000 cohort participants attended the study centre to repeat

		the baseline measures and health survey. Follow-up is continuing. The study's main focus has been on identifying risk factors for cancer and other chronic diseases, such as type 2 diabetes, cardiovascular disease, eye disease, and arthritis. Through data collected from this contemporary large cohort study, the investigators are studying the determinants of chronic disease, with the aim of developing prediction tools applicable to the current Australian population. Results from this study will allow future patterns of chronic disease to be accurately forecasted, which in turn permits preventive strategies to be used in a more effective manner. The MCCS does not provide data on incident cardiovascular disease events.<br>Written informed consent was obtained from all study participants.<br>https://www.cancervic.org.au/research/epidemiology/health_2020/health2020-overview
Australia	MONICA Newcastle	The MONICA Newcastle cohort consists of residents aged 25–69 years of the five local government areas of Newcastle, Lake Macquarie, Port Stephens, Maitland, and Cessnock participated in the MONICA Project in Newcastle. There were 76,831 men and 76,502 women aged 35 to 64 years in the study population in 1991. Three surveys of risk factors were conducted during the study period, the first in 1983, the second in 1988 and 1989 and the third in 1994. The subjects were followed-up for fatal events until 22nd December 1998 by using the national death index which is linked to data from the Australian Institute of Health and Welfare and AIHW National Mortality Database. All participants gave informed consent.<br>https://www.maelstrom-research.org/study/monica-newcastle

Description of health examination survey data

North America

Country	Study	Study/cohort full name and short description
USA	NHANES	The National Health and Nutrition Examination Survey (NHANES) is a program of studies designed to assess the health and nutritional status of adults and children in the United States. The survey is unique in that it combines interviews and physical examinations. NHANES is a major program of the National Center for Health Statistics (NCHS). NCHS is part of the Centers for Disease Control and Prevention (CDC) and has the responsibility for producing vital and health statistics for the Nation. The NHANES program began in the early 1960s and has been conducted as a series of surveys focusing on different population groups or health topics. In 1999, the survey became a continuous program that has a changing focus on a variety of health and nutrition measurements to meet emerging needs. The survey examines a nationally representative sample of about 5,000 persons each year. These persons are located in counties across the country, 15 of which are visited each year. The NHANES interview includes demographic, socioeconomic, dietary, and health-related questions. The examination component consists of medical, dental, and physiological measurements, as well as laboratory tests administered by highly trained medical personnel. Findings from this survey will be used to determine the prevalence of major diseases and risk factors for diseases. Information will be used to assess nutritional status and its association with health promotion and disease prevention. NHANES findings are also the basis for national standards for such measurements as height, weight, and blood pressure. Data from this survey will be used in epidemiological studies and health sciences research, which help develop sound public health policy, direct and design health programs and services, and expand the health knowledge for the Nation.<br><br>https://www.cdc.gov/nchs/nhanes/about_nhanes.htm

Latin America

Country	Study	Study/cohort full name and short description
Brazil	Pesquisa Nacional de Saúde	Pesquisa Nacional de Saúde (PNS) is a Brazilian national health examination survey that was initiated in 2013. The survey is a collaboration of the national ministry for health and the Brazilian institute for geography and statistics. The aim of PNS is to collect data on Brazilian health and lifestyle data with a focus on access to healthcare services, the continuous access to healthcare and healthcare expenditure. PNS collects data through survey questionnaires and computer assisted interviews.<br><br>https://www.ibge.gov.br/estatisticas/sociais/saude/9160-pesquisa-nacional-de-saude.html?=&t=conceitos-e-metodos<br><br>https://pubmed.ncbi.nlm.nih.gov/33027428/
Chile	Encuesta Nacional de Salud	The Encuesta Nacional de Salud (ENS) is a national health examination survey in Chile that started 2016 and is repeated every 5 years. ENS investigates the health status of men and women from 15 years on. The collected data offers important insides on prevalence of around 60 diseases and risk factors. The participants first get a survey questionnaire and later undergo clinical examinations that follow a specific guideline and are executed by trained medical staff. ENS collects data in 15 regions of Chile.<br><br>http://epi.minsal.cl/encuesta-ens/
Guyana	WHO STEPS<br><br>Guyana	The WHO STEPwise Approach to NCD Risk Factor Surveillance (STEPS) is a simple, standardized method for collecting, analyzing and disseminating data on key NCD risk factors in countries. The survey instrument covers key behavioral risk factors: tobacco use, alcohol use, physical inactivity, unhealthy diet, as well as key biological risk factors: overweight and obesity, raised blood pressure, raised blood glucose, and abnormal blood

		lipids. Through the use of expanded modules, the survey instrument can be expanded to cover a range of topics beyond these risk factors, such as oral health, sexual health and road safety. By using the same standardized questions and protocols, all countries can use STEPS information not only for monitoring within-country trends, but also for making comparisons across countries. The approach encourages the collection of small amounts of useful information on a regular basis.<br><br>https://www.who.int/teams/noncommunicable-diseases/surveillance/systems-tools/steps
Mexico	ENSANUT	The National Health and Nutrition Survey (ENSANUT) in Mexico is a fundamental reference for monitoring and evaluating the performance of the health system. Since 2006 the National Institute of Public Health combined the different existing health and nutrition surveys into ENSANUT. ENSANUT is conducted at the end of every federal administration period, that is, every six years. The general objective of the ENSANUT is update the panorama on the frequency, distribution and trends of health and nutrition conditions, and their determinants, and the demand for health services by the population, as well as examine the State's response. Additionally, as a secondary objective, it seeks to collect information to estimate the trend in the prevalence of chronic diseases with the greatest impact: diabetes, hypertension and obesity. ENSANUT covers nine different regions of Mexico. For the state of Guanajuato, which belongs to the Central-North region, an oversampling was carried out to select at least 1,250 dwellings. The sampling frame of the primary sampling units (UPM) is a list of Basic Geostatistical Areas (AGEB) built from the AGEB defined by the National Institute of Geography and Statistics (INEGI). The rural AGEBs from the 2005 Population and Housing Count were used to form conglomerates of rural localities from the 2010 Population and Housing Census; On the other hand, in urban localities, the AGEBs of the 2010 Census were used as conglomerates of urban blocks. Data was obtained via standardized questionnaires and medical examinations. All participants or their parents or guardians gave written informed consent.<br><br>https://www.ncbi.nlm.nih.gov/pmc/articles/PMC6723052/

Western Europe

Country	Study	Study/cohort full name and short description
Finland	FINHEALTH 2017	The National FinHealth 2017 Study is a Finnish population-based survey study investigating health and well-being of Finnish residents. The study covers topics like perceived health, quality of life, lifestyle, need for care and assistance, use of health services and prevalence of risk factors for common health problems. The data is collected through questionnaires and a physical examination. For this analysis we used a dataset of 6665 individuals.<br><br>https://thl.fi/en/web/thlfi-en/research-and-development/research-and-projects/national-finhealth-study
Germany	DEGS1	The German Health Interview and Examination Survey for Adults (DEGS1, Studie zur Gesundheit Erwachsener in Deutschland) is a randomly chosen national sample of the adult population in Germany age between 18-79 years. Between 2008 and 2011 DEGS1 recruited 8,152 subjects (4,193 newly recruited participants with a response rate of 42% and 3,959 who had previously taken part in GNHIES98 with a response rate of 62%). It aims to provide current and normative data for the clinical research and investigate the health-related quality of life in Germany. The survey includes a visit to a local study center for examinations and a questionnaire for the survey. DEGS1 was approved by the local IRB and participants prove informed consent.<br><br>https://www.degs-studie.de/english/home.html
Germany	HCHS	The Hamburg City Health Study (HCHS) is a long-term population-based cohort study that included a random sample age 45-74 from the general population of Hamburg, Germany.

After giving informed consent, the participants undergo extensive examinations for several hours targeting the major organ system function and structures. Among other topics, Lifestyle, dietary habits, environmental conditions, medical and family history, and physical activity are assessed through self-reported questionnaires. The examination is completed with a characterization of the genome and proteome. Subjects at risk for coronary artery disease, atrial fibrillation, heart failure, stroke and dementia are invited to an additional visit to conduct an additional MRI examination of either heart or brain. HCHS aims to include 45.000 participants in total, however, for this analysis we included only 10.000 participant. Data from the Statistisches Bundesamt (DESTATIS) was used to weight the data and make it more representative in terms of sex and age distribution. The estimates used for this were from 2011.

https://pubmed.ncbi.nlm.nih.gov/31705407/

Eastern Europe and Russia

Country	Study	Study/cohort full name and short description
Russia	Know Your Heart	The Know Your Heart Study was started in 2015 and recruited 4542 individuals of the general Russian population aged 35-69 by 2018. The study took place in Novosibirsk and Arkhangelsk and included a baseline interview to collect information on socio-demographic characteristics and cardiovascular risk factors and a comprehensive health check which included detailed examination of the cardiovascular system. Among its aims are the characterization of CVD phenotypes in the Russian population, differences in CVD phenotypes in Novosibirsk and Arkhangelsk and to investigate the associations of CVD phenotypes with health behaviours and socio-demographic factors, as well as, known CVD risk factors.<br><br>Ethical approval for the study was received from the ethics committees of the London School of Hygiene & Tropical Medicine (approval number 8808 received 24.02.2015; for sub-study involving patients in treatment for alcohol problems approval number 12018; received 11/01/2017), Novosibirsk State Medical University (approval number 75 approval received 21/05/2015), the Institute of Preventative Medicine (no approval number ; approval received 26/12/2014), Novosibirsk and the Northern State Medical University, Arkhangelsk(approval number 01/01-15 received 27/01/2015; for sub-study involving patients in treatment for alcohol problems approval number 05/11-16 received 02/11/2016). Signed informed consent was obtained both at baseline interview and at the health check.<br><br>The International Project on Cardiovascular Disease in Russia (IPCDR) was supported in part by a Wellcome Trust Strategic Award [100217]. https://wellcomeopenresearch.org/articles/3-67/v3
Belarus	WHO STEPS<br><br>Belarus	The WHO STEPS survey Belarus is a population-based survey that was carried out between 2016 and 2017 and included a total of 5760 participants aged 18-69. A Multi-stage cluster sampling design was used to produce representative data for that age range in Belarus. The overall response rate was 87.1%. The survey collected data on socio-demographics, medical history, behavioural information, and physical and biochemical measurements.<br><br>https://extranet.who.int/ncdsmicrodata/index.php/catalog/100
Georgia	WHO STEPS<br><br>Georgia	The WHO STEPS survey Georgia was established in 2010 and is a population-based survey of adults age 18-69 years. For this analysis the second STEPS survey from 2016 was used. A multi-stage cluster sampling design was used to produce representative data for that age range in Georgia. A total of 5554 adults were included in the survey. The overall response rate was 75.7%. The survey collected data on socio-demographics, medical history, behavioural information, and physical and biochemical measurements.<br><br>https://extranet.who.int/ncdsmicrodata/index.php/catalog/223

Tajikistan	WHO STEPS Tajikistan	The WHO STEPS survey Tajikistan was established in 2016 and is a population-based survey of adults age 18-69 years. For this analysis the STEPS survey from 2016/2017 was used. A multi-stage cluster sampling design was used to produce representative data for that age range in Tajikistan. A total of 2718 adults were included in the survey. The overall response rate was 94.3%. The survey collected data on socio-demographics, medical history, behavioural information, and physical and biochemical measurements.<br><br>https://extranet.who.int/ncdsmicrodata/index.php/catalog/270
Turk-menistan	WHO STEPS Turk-menistan	The WHO STEPS survey Turkmenistan was established in 2013 and is a population-based survey of adults age 18-69 years. For this analysis the second STEPS survey from 2018 was used. A multi-stage cluster sampling design was used to produce representative data for that age range in Turkmenistan. A total of 4053 adults were included in the survey. The overall response rate was 93.8%. The survey collected data on socio-demographics, medical history, behavioural information, and physical and biochemical measurements.<br><br>https://extranet.who.int/ncdsmicrodata/index.php/catalog/854

Sub-Saharan Africa

Country	Study	Study/cohort full name and short description
South Africa	SANHANES	The South African National Health and Nutrition Examination Survey (SANHANES) was established as a continuous population health survey to address the changing health needs in the country and provide a broader and more comprehensive platform to study the health status of the nation on a regular basis. The SANHANES-1, was conducted in 2011-2012 among 27,580 eligible individuals, of which 25,532 individuals completed the interview, 12,025 underwent physical examinations and 8,078 provided blood specimens for biomarker testing. This survey provides critical information to map the emerging epidemic of non-communicable diseases (NCDs) in South Africa among other defined priorities of the National Department of Health and analyses their social, economic, behavioural and environmental determinants. Data on the magnitude of and trends in NCDs, as well as other existing/emerging health priorities, is essential to develop national prevention and control programmes, assessing the impact of interventions, and evaluating the health status of the country. The primary objectives of the SANHANES-1 were to assess defined aspects of the health and nutritional status of South Africans with respect to the prevalence of NCDs (specifically cardiovascular disease, diabetes, and hypertension) and their risk factors (diet, physical activity and tobacco use). Among other aims, SANHANES-1 investigates the behavioural (smoking, diet, physical inactivity) and social determinants of health and nutrition (demographic, socioeconomic status, and locality) and how these relate to the health and nutritional status of the population. The survey covers 9 provinces of South Africa.<br><br>Every participant that agreed to the interviews, physical examinations and blood analyses gave informed consent.<br><br>https://repository.hsrc.ac.za/handle/20.500.11910/2864
Eswatini	WHO STEPS Eswatini	The WHO STEPS survey Eswatini (or Swaziland) was established in 2014 and is a population-based survey of adults age 15-69 years. For this analysis the STEPS survey from 2014 was used. A multi-stage cluster sampling design was used to produce representative data for that age range in Eswatini. A total of 3281 adults were included in the survey. The overall response rate was 76%. The survey collected data on socio-demographics, medical history, behavioural information, and physical and biochemical measurements. Additionally, it contains an extensive questionnaire on tobacco use.<br><br>https://extranet.who.int/ncdsmicrodata/index.php/catalog/688

Ethiopia	WHO STEPS Ethiopia	The WHO STEPS survey Ethiopia was established in 2015 and is a population-based survey of adults age 15-69 years. For this analysis the STEPS survey from 2014 was used. A multi-stage cluster sampling design was used to produce representative data for that age range in Ethiopia. A total of 9801 adults from eleven regions were included in the survey. The overall response rate was 95.5%. The survey collected data on socio-demographics, medical history, behavioural information, and physical and biochemical measurements. Only permanent residents of Ethiopia were included in this survey. All data was collected unlinked and anonymously. All participants under 18 years of age provided an additional informed consent from their parents or guardians.<br><br>https://extranet.who.int/ncdsmicrodata/index.php/catalog/794
Sudan	WHO STEPS Sudan	The WHO STEPS survey Sudan was established in 2016 and is a population-based survey of adults age 18-69 years. For this analysis the STEPS survey from 2016 was used. A multi-stage cluster sampling design was used to produce representative data for that age range in Sudan. A total of 8145 adults were included in the survey. The overall response rate was 95%. The survey collected data on socio-demographics, medical history, behavioural information, and physical and biochemical measurements.<br><br>https://extranet.who.int/ncdsmicrodata/index.php/catalog/438

Middle East and North Africa

Country	Study	Study/cohort full name and short description
Azerbaijan	WHO STEPS Azerbaijan	The WHO STEPS survey Azerbaijan is a population-based survey that was carried out in 2017 and included a total of 1852 households from 240 regional clusters and included participants age 18-69 years. A multi-stage cluster sampling design was used to produce representative data for that age range in Azerbaijan. The survey collected data on socio-demographics, medical history, behavioural information, and physical and biochemical measurements. 2880 individual adults were included in this survey.<br><br>https://extranet.who.int/ncdsmicrodata/index.php/catalog/127
Iraq	WHO STEPS Iraq	The WHO STEPS survey Iraq was established in 2006 and is a population-based survey of adults age 18 years or older. For this analysis the second STEPS survey from 2015 was used. A multi-stage cluster sampling design was used to produce representative data for that age range in Iraq. A total of 4120 adults were included in the survey. The overall response rate was 93.5%. The survey collected data on socio-demographics, medical history, behavioural information, and physical and biochemical measurements. Additionally, the survey also tested the concentration of salt in urine samples.<br><br>https://extranet.who.int/ncdsmicrodata/index.php/catalog/420
Jordan	WHO STEPS Jordan	The WHO STEPS survey Jordan was established in 2007 and is a population-based survey of adults age 18-69 years. For this analysis the second STEPS survey from 2019 was used. A multi-stage cluster sampling design was used to produce representative data for that age range in Jordan. A total of 5713 adults were included in the survey. The response rate was 63%. The survey collected data on socio-demographics, medical history, behavioural information, and physical and biochemical measurements.<br><br>https://extranet.who.int/ncdsmicrodata/index.php/catalog/853

Lebanon	WHO STEPS Lebanon	The WHO STEPS survey Lebanon was established in 2016 and is a population-based survey of adults age 18-69 years. A multi-stage cluster sampling design was used to produce representative data for that age range in Lebanon. As Lebanon has a significant number of Syrian immigrants and refugees, a Lebanese (n=1899) and a Syrian sample (n=2134), was created. For this analysis both STEPS survey samples from 2017 were used. A total of 4033 adults were included in the survey. The overall response rate for the Lebanese sample was 65.9% and 74.1% for the Syrian sample. The survey collected data on socio-demographics, medical history, behavioural information, and physical and biochemical measurements.<br><br>https://extranet.who.int/ncdsmicrodata/index.php/catalog/410

Asia

Country	Study	Study/cohort full name and short description
Bangladesh	WHO STEPS Bangladesh	The WHO STEPS survey Bangladesh was established in 2009 and is a population-based survey of adults age 18-69 years. For this analysis the second STEPS survey from 2018 was used. A multi-stage cluster sampling design was used to produce representative data for that age range in Bangladesh. A total of 8185 adults were included in the survey. The overall response rate was around 80%. The survey collected data on socio-demographics, medical history, behavioural information, and physical and biochemical measurements. Additionally, the survey collected data on oral health and an assessment of the national health system and its ability to detect and treat key risk factors for non-communicable diseases.<br><br>https://apps.who.int/iris/rest/bitstreams/1284802/retrieve
Bhutan	WHO STEPS Bhutan	The WHO STEPS survey Bhutan was established in 2014 and is a population-based survey of adults age 15-6 years. For this analysis the second STEPS survey from 2019 was used. A multi-stage cluster sampling design was used to produce representative data for that age range in Bhutan. A total of 5575 adults were included in the survey. The overall response rate was around 94.8%. The survey collected data on socio-demographics, medical history, behavioural information, and physical and biochemical measurements.<br><br>https://extranet.who.int/ncdsmicrodata/index.php/catalog/855/download/6026
Myanmar	WHO STEPS Myanmar	The WHO STEPS survey Myanmar was carried out from September to December in 2014. The survey was a population-based survey of adults age 25-64 years. A multi-stage cluster sample design was used to produce representative data for that age range from 52 townships in Myanmar. A total of 8757 adults participated in the survey. The survey collected information on sociodemographic and behavioural characteristics, physical measurements such as height, weight and blood pressure and biochemical measurements (blood glucose-both fasting and 2-h PG, levels of total cholesterol, triglycerides, HDL and LDL. The overall response rate was 94% for risk factors, 91% for physical measurements and 90% for biochemical measurements.<br><br>https://www.who.int/publications/m/item/2014-steps-country-report-myanmar

Australia

Country	Study	Study/cohort full name and short description
Australia	AusDiab	The baseline study conducted in 1999–2000 provided benchmark national data on the prevalence (or number of people) of diabetes, obesity, hypertension, and kidney disease in Australia. The second phase of AusDiab, completed in December 2005, was a five-year follow-up of the people who participated in the baseline survey. A twelve-year follow-up was completed in 2012. AusDiab is a national population-based longitudinal study established to examine the prevalence of diabetes and related risk factors in Australia. Using a stratified cluster sampling method, it enrolled 11,247 adults (aged 25 years and above) from 42 randomly selected census collector districts across Australia between May 1999 and December 2000. Over 85% of the participants were from an Australian, New Zealand or British background. The baseline survey consisted of an initial household interview and was followed by biomedical exams. At baseline and follow-ups, anthropometric measures, and fasting blood samples were collected and participants completed interviewer-administered questionnaires on health and lifestyle factors. Participants underwent a standard 75 g oral glucose tolerance test and T2D was classified as fasting plasma glucose ≥7.0 mmol/L, 2 h plasma glucose ≥ 11.1 mmol/L or current treatment with insulin or oral hypoglycemic agents.<br><br>This study was weighted to be used as Health Examination Survey. The information downloaded from the Australian Bureau of Statistics (https://www.abs.gov.au/). The estimates from 2021 were used for age and sex distribution.<br><br>Informed consent was obtained from all participants.<br><br>https://www.baker.edu.au/ausdiab/

Data harmonization

A list of variables required for this project, including their definitions, was created based on the definitions used for variables in the MONICA/MORGAM project.[3] Centers representing studies that did not belong to the MORGAM project received a list of variables with definitions and were asked to provide those variables, if possible, harmonized with the MORGAM project definitions.

With regards to age, sex and ethnicity, the participating studies were asked to provide the age of the participant at the date of examination (in years); sex (coded as male or female); and ethnicity information (coded as American Indian or Alaska native, Asian, Black or African American, Hispanic or Latino, Hawaiian or Other Pacific Islander, White). In the MORGAM project, ethnicity information, when available, categorized individuals as European or non-European.

Data from centers not taking part in the MORGAM project were transferred to Hamburg. If the dataset was not harmonized with the MORGAM definitions, this was done by the study investigators in Hamburg. Each transferred dataset was examined by a data analyst, who further harmonized the data and communicated with the individual or group that provided the data when clarifications were needed. After the first round of harmonization was performed, a second data analyst examined the data as harmonized by the first analyst and updated the harmonization if needed. Automatic controls were built-in in the code used during the harmonization process (e.g., checking that categorical variables take only allowed values or checking if negative follow-up times were present). When issues with the data harmonization became apparent only after merging the different datasets, the harmonization was reviewed again, and the dataset was updated.

Statistical methods

Multiple imputation by chained equations[4] was performed separately for each study and for each sex, with the exception of the Kiang West Longitudinal Population Study dataset where information from other Sub-Saharan African datasets was used during the imputation process. Follow-up data for the end points considered was included in the imputation models via the event indicator and the Nelson-Aalen estimate of the cumulative hazard function as recommended by White et al.[5] Available observations per variable and according to geographic region are provided in **Table S4**. Ten imputed datasets were generated.

Continuous variables are presented as quartiles or minimum and maximum, and binary variables as absolute and relative frequencies. Regional baseline characteristics were estimated with and without direct standardization according to the age and sex distribution of the GCVRC dataset. The following age groups were used in the standardization: age ≤40 years; >40 to ≤45; >45 to ≤50; >50 to ≤55; >55 to ≤60; >65 to ≤70; and >70 years.

The median (25th, 75th percentile) duration of follow-up was computed using the Kaplan-Meier potential follow-up estimator.[6]

For each sex, ten-year incidences and events per 1000 person years were estimated. Direct standardization according to the age distribution of the GCVRC dataset for each sex, was used when computing these estimates for each geographic region. Poisson regression models with follow-up time that was log-transformed as an offset were used in the estimation of the events per 1000 person years.

Cumulative incidence curves with a) time-on-study and b) age as the time scale were estimated for cardiovascular disease and all-cause mortality. For the cardiovascular disease end point non-cardiovascular disease related death was treated as competing event and the Aalen-Johansen estimator was used.[7]

Risk factors and outcome events associations were estimated using a two-stage random effects multivariate individual participant data meta-analysis.[8] More precisely, sex-specific Cox models were computed for each study, then coefficients were pooled across studies by region as well as over all datasets using multivariate random effects meta-analysis. All models used age as the time scale.[9] Besides age, all models included the following variables: body-mass index (BMI), systolic blood pressure (SBP), non-high-density lipoprotein (HDL) cholesterol, current smoking, diabetes, and use of antihypertensive medications. Different sets of Cox models were computed. First all covariates were entered into the model linearly. Then the continuous covariates (BMI, SBP and non-HDL cholesterol) were modelled using restricted cubic splines with 4 knots. The same knots were used for each study. The last set of models allowed for the effects to change with age (the time scale). In the event that hazard ratios do not remain constant over age, the hazard ratio can be interpreted as age-averaged effect.[10] A one-year landmark analysis was used in all these models (only those individuals surviving at least one year were used in the computations). The use of lipid-lowering medications was not included in the aforementioned models, because it was missing in approximately 20% of the individuals. Sensitivity analyses were performed: a) restricting the data to those cohort studies with baseline examination at or after the year 2000; b) using an alternative definition of cardiovascular disease, which modifies the definition used by removing the angina, revascularization, and unclassifiable death components; c) Including lipid-lowering medications as an additional covariate in the models; d) using a two-year landmark instead of a one-year landmark. Sensitivity analyses using an alternative

definition of cardiovascular disease was performed only in a subgroup of cohort studies where the alternative definition had been harmonized. Sensitivity analyses computed Fine and Gray models were performed. Fine and Gray models can be computed by calculating a weighted Cox regression using a special dataset, obtained from the original data, together with time-dependent weights to account for left-truncation and censoring.[11] The special dataset can be much larger than the original data and the required computations were not feasible for all cohorts. Fine and Gray models were computed on those datasets where the created special dataset had less than six million rows.

Population-attrbutable fractions (PAF) for the 10-year incidence of cardiovascular disease and all-cause mortality were estimated using the methods from Laaksonen and colleagues.[12] This approach allows the estimation of population-attributable fractions for combinations of risk factors. Time-to-event models, similar to the ones used in the association analyses but with categorized covariates, were constructed using the cohort studies and then applied to each individual in the contemporary health examination surveys to estimate the population-attributable fractions.

PAF estimates the proportion of the outcomes that could be avoided if some of the risk factors could be changed to given target values. For incident cardiovascular disease the computations were based on the following formula[12]:

$$PAF(t) = 1 - \frac{\sum_{i=1}^{n} P\{T_i^{CVD} \leq \min(T_i^M, t) \,|X_i^*\}}{\sum_{i=1}^{n} P\{T_i^{CVD} \leq \min(T_i^M, t) \,|X_i\}},$$

where $T_i^{CVD}$is the time to CVD for individual i; $T_i^M$is the time to death for individual i; X_i are the covariate values for individual i; X_i^* are the covariate target values for individual i; $P\{T_i^{CVD} \leq min(T_i^M, t)|X_i\}$ is the probability of developing CVD before time t given the covariates X_i accounting for death as competing event; $P\{T_i^{CVD} \leq min(T_i^M, t)|X_i^*\}$ is the probability of developing CVD before time t given the target covariates X_i^* accounting for death as competing event. A similar, but simpler, formula was used for all-cause mortality since there are no competing events. This approach gives the estimation of PAF for combinations of risk factors, without producing estimates that exceed 100%.

Weibull proportional hazard models were used to estimate the 10-year event probabilities required for the PAF estimation. Specifically, cause-specific Weibull models were used in the case of cardiovascular disease, treating death from non-cardiovascular disease causes as competing events. As it was done with the Cox regressions, a two-stage random effects multivariate individual participant data meta-analysis was used. Age was used as the time scale and the following variables were included simultaneously in the models: BMI (categorized), SBP (categorized), non-HDL cholesterol (categorized), current smoking, diabetes, and use of antihypertensive medications. BMI was grouped according to an adapted version of the WHO classification (BMI <20 kg/m2; 20 to <25 kg/m2 (reference category); 25 to <30 kg/m2; ≥30 kg/m2). The cut-off value of 20 instead of 18.5 kg/m2 was used because of the low number of individuals with BMI <18.5kg/m2, as was done by Leong et al.[13] SBP was categorized as <120 mmHg (reference category), 120 to <140 mmHg, 140 to <160 mmHg, and ≥160 mmHg; and non-HDL cholesterol as < 116.01 mg/dL (reference category), 116.01 to < 154.68 mg/dL , 154.68 to < 193.35 mg/dL, and ≥ 193.35 mg/dL. Current smoking and diabetes were used as binary variables (yes/no), with the “no” category being the reference. The distributional assumptions of the Weibull model was checked for each end point, cohort and sex, as described in Harrell.[14] Specifically, the estimated survival function of the standardized residuals was plotted alongside the survival function of the assumed distribution. For the former the Kaplan-Meier estimator was used. The models did not allow

for time-varying coefficients, and we are not aware of any implementation that allows for computation of probabilities for such models. As the population-attributable fractions vary with the prevalence of the risk factor, which may have changed over time, as may have risk factor management,[15,16] we used contemporary cross-sectional national health examination surveys (from 2010 or later) to estimate contemporary risk factor prevalence when calculating the population-attributable fractions. The only exception was the data used for Australia, where the baseline assessment was conducted in 1999-2000. Individual-level survey data of 153,982 individuals, which, after weighting of the sample represented a total of 874,105,228 inhabitants from the different geographic regions, were available for analyses. Each national health examination survey is described above. We apply the term cohort study to refer to those population-based cohort studies used to perform any time-to-event analyses and the term health examination *survey* to refer to the surveys and studies of a cross-sectional nature used for contemporary risk factor prevalence. The pooled Weibull models were applied to the individuals in the health examination surveys. Specifically, the parameters of the models used to compute the 10-year probability of event (cardiovascular disease or all-cause mortality) were estimated using the cohort studies data, but the actual probabilities used in the formula above were estimated in the health examination surveys. For this purpose, only the global models were used. Confidence intervals were computed with help of the bootstrap, with 250 repetitions, as follows: first the parameters of the pooled Weibull models were sampled from a multivariate normal distribution using the mean vector and covariance matrix estimated in the original data; afterwards the new models obtained this way were applied to a dataset drawn with replacement from the health examination surveys; and then PAFs were computed based on the bootstrapped Weibull parameters and bootstrapped health examination surveys. The methods used allow for the estimation of PAFs for single risk factors or any combination of them, including an aggregated PAF in which all risk factors are set to their target values simultaneously. As it was done with the Cox models, a one-year landmark analysis was used in the computation of the Weibull models. Sensitivity analyses were performed using Weibull models that included only one risk factor at a time (not adjusting for other risk factors).

For all time-to-event analyses, individuals with cardiovascular disease at baseline were excluded. Presented confidence intervals were not adjusted for multiple comparisons and should not be used in place of hypothesis testing. All statistical methods were implemented in R statistical language, version 4.1.3.[17]

Supplementary Figures

Figure S1. Flow chart.

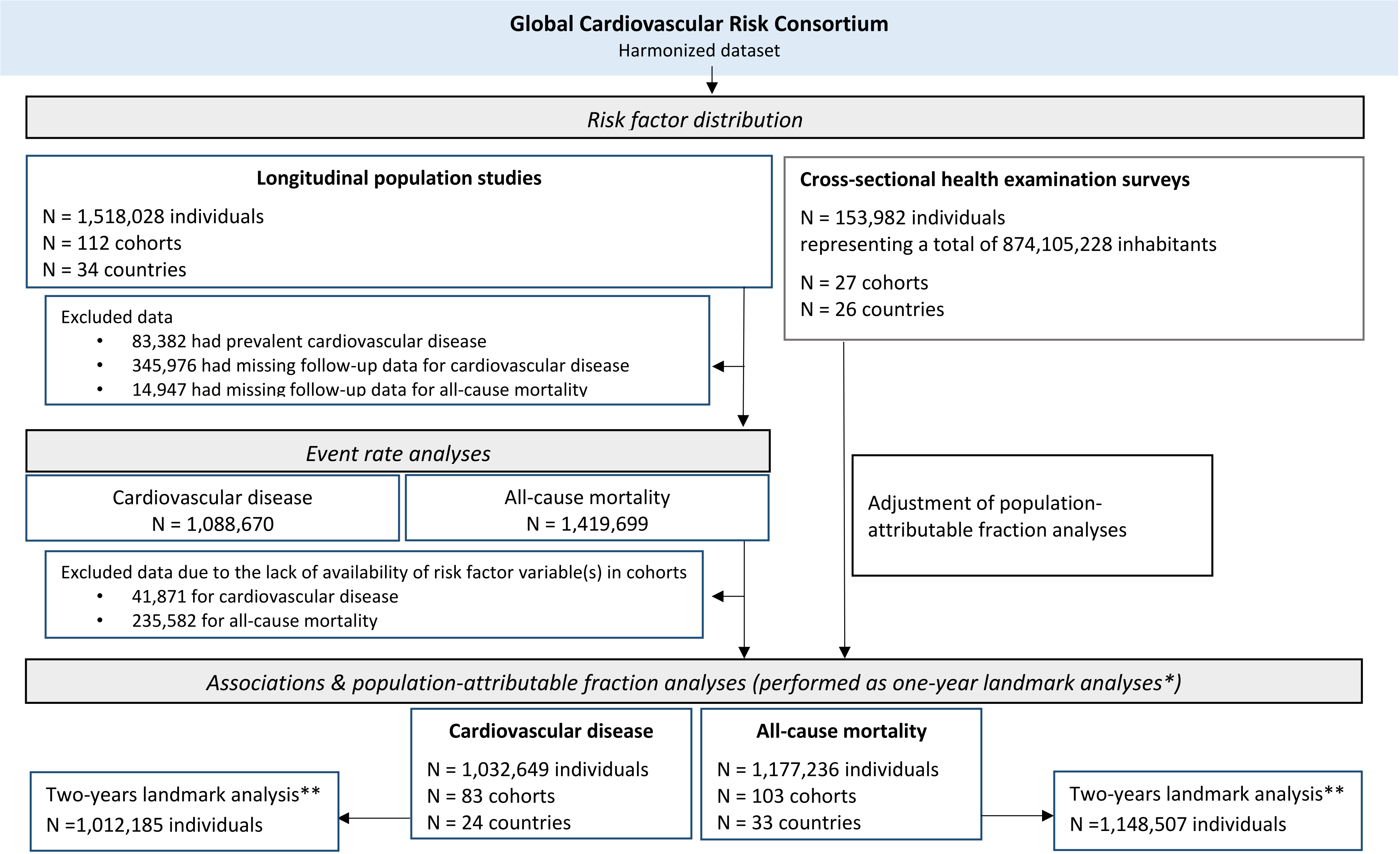

*Excluded individuals with less than one year of follow-up for the specified end point: N=14,150 (cardiovascular disease): N= 6,881 (all-cause mortality)

** Excluded individuals with less than two years of follow-up for the specified end point (only done in association analyses): N=20,464 (cardiovascular disease) and N=28,729 (all-cause mortality)

Figure S2. Sex-specific cumulative incidences for individuals 40 years of age for a) cardiovascular disease and b) all-cause mortality using age as time scale. *Individuals with cardiovascular disease at baseline were excluded. Confidence interval widths have not been adjusted for multiplicity and should not be used in place of hypothesis testing.*

a) Cardiovascular disease

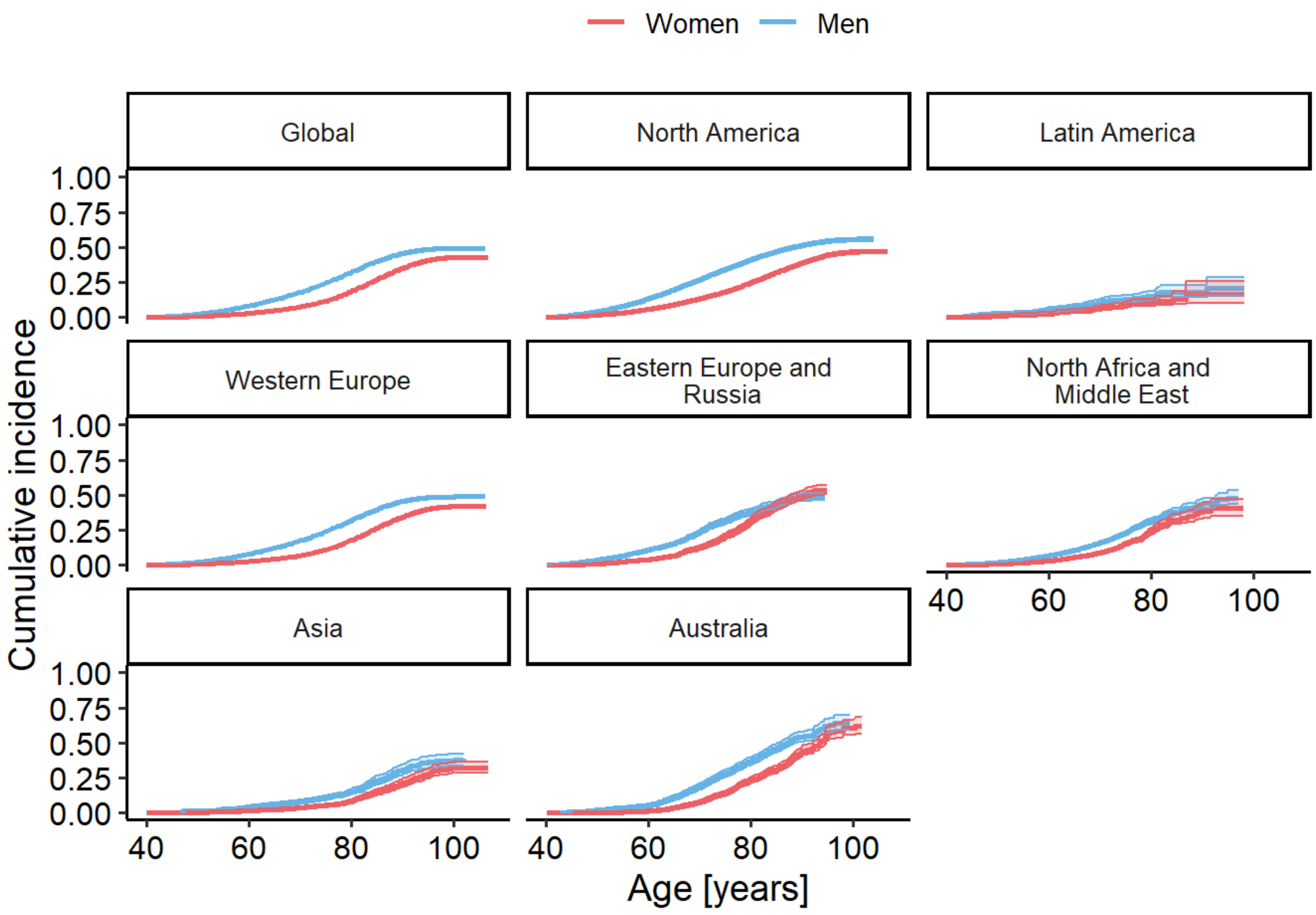

b) All-cause mortality

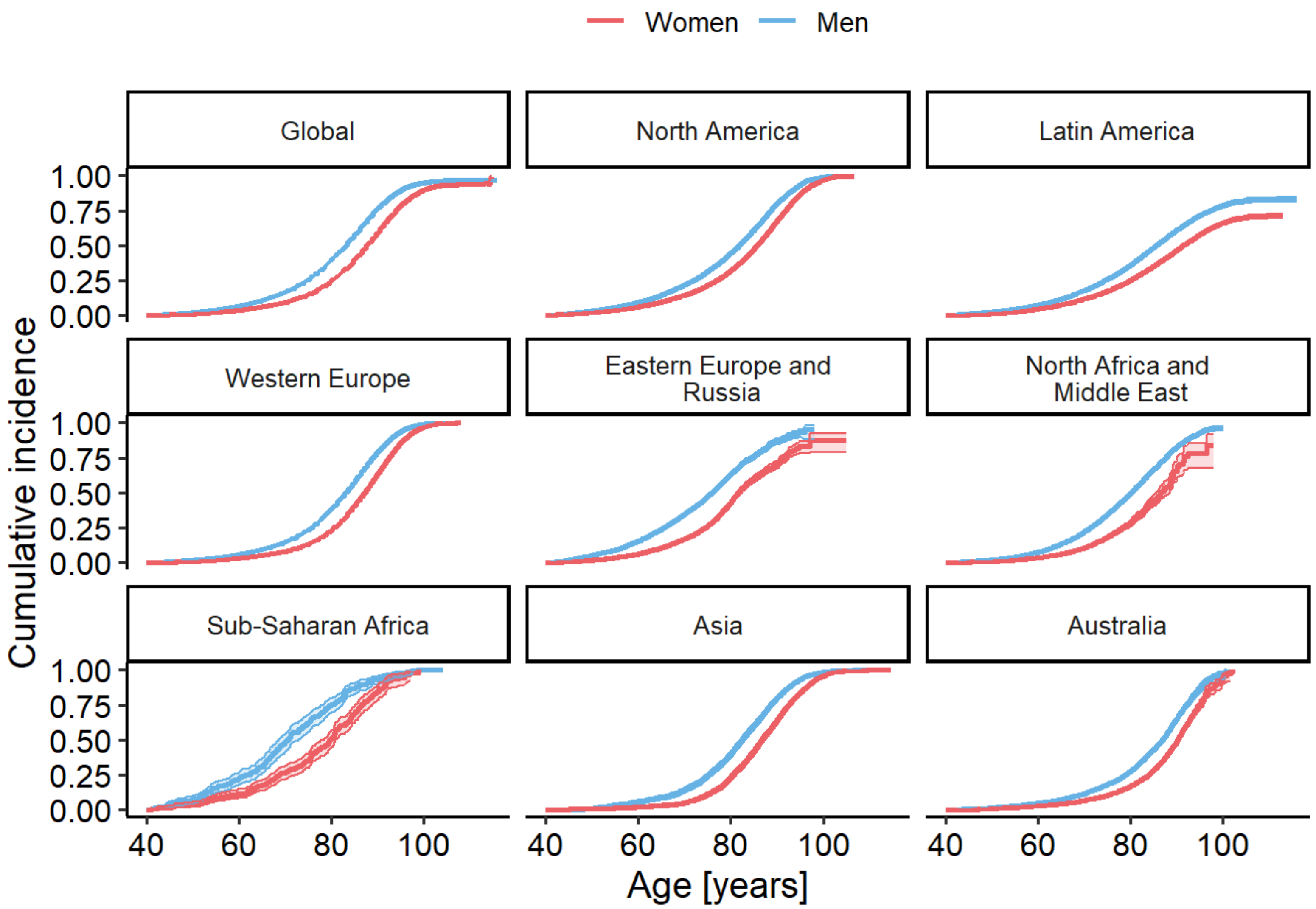

Figure S3. Categorized body-mass index hazard ratios for a) cardiovascular disease and b) all-cause mortality.
Individuals with cardiovascular disease at baseline were excluded. Age was used as the time scale. Besides categorized body-mass index the models included the following covariates: systolic blood pressure, non-HDL cholesterol, current smoking, diabetes and use of antihypertensive medications. A one-year landmark analysis was performed. Confidence interval widths have not been adjusted for multiplicity and should not be used in place of hypothesis testing.

a) Cardiovascular disease

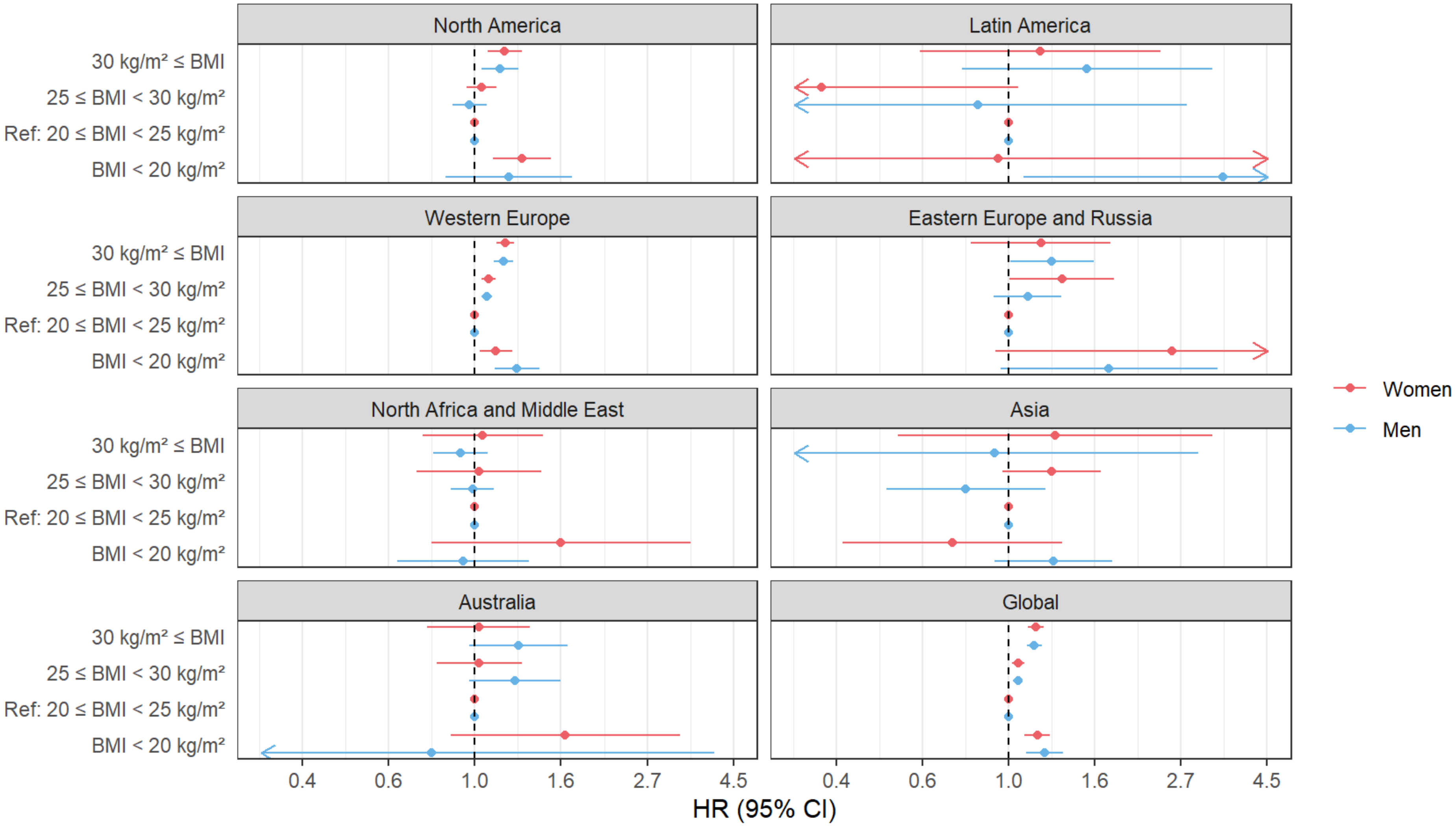

b) All-cause mortality

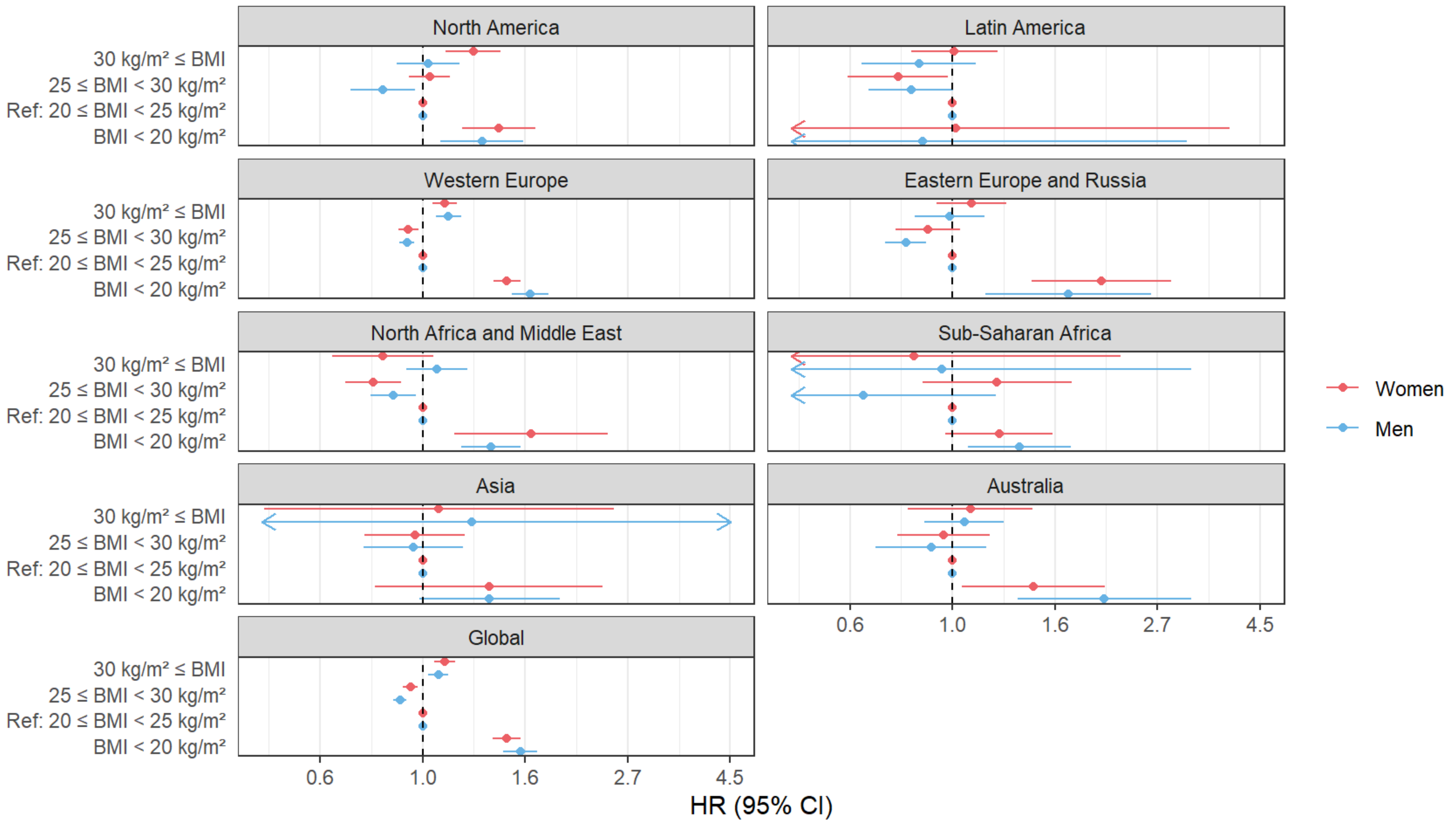

Figure S4. Associations of continuous risk factors with a) cardiovascular disease and b) all-cause mortality allowing for non-linear effects by geographic region. *Individuals with cardiovascular disease at baseline were excluded. Age was used as the time scale. All five risk factors considered were included in the models together with use of antihypertensive medications. A one-year landmark analysis was performed. Confidence interval widths have not been adjusted for multiplicity and should not be used in place of hypothesis testing. To convert the values for non-HDL cholesterol from milligrams per deciliter (mg/dL) to millimoles per liter (mmol/L), multiply by 0.02586.*

a) Cardiovascular disease

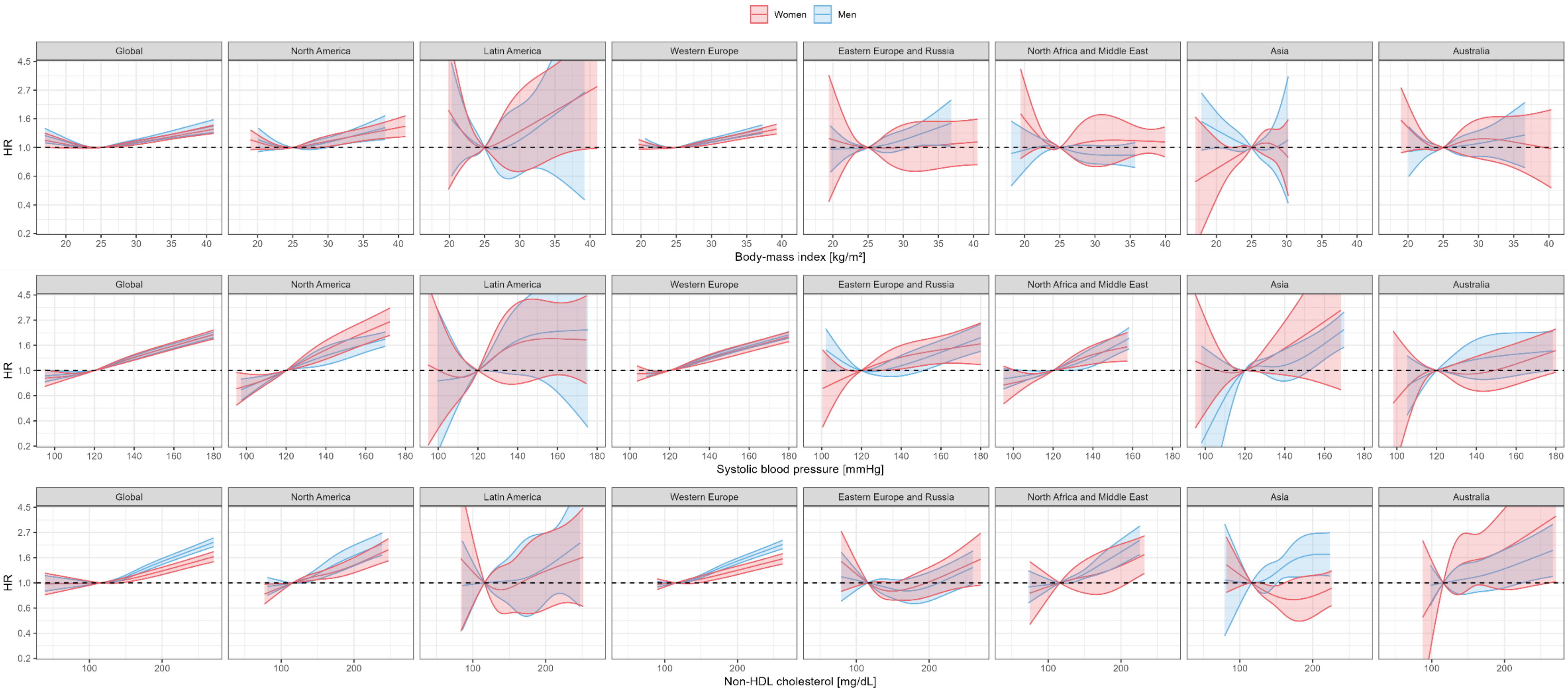

b) All-cause mortality

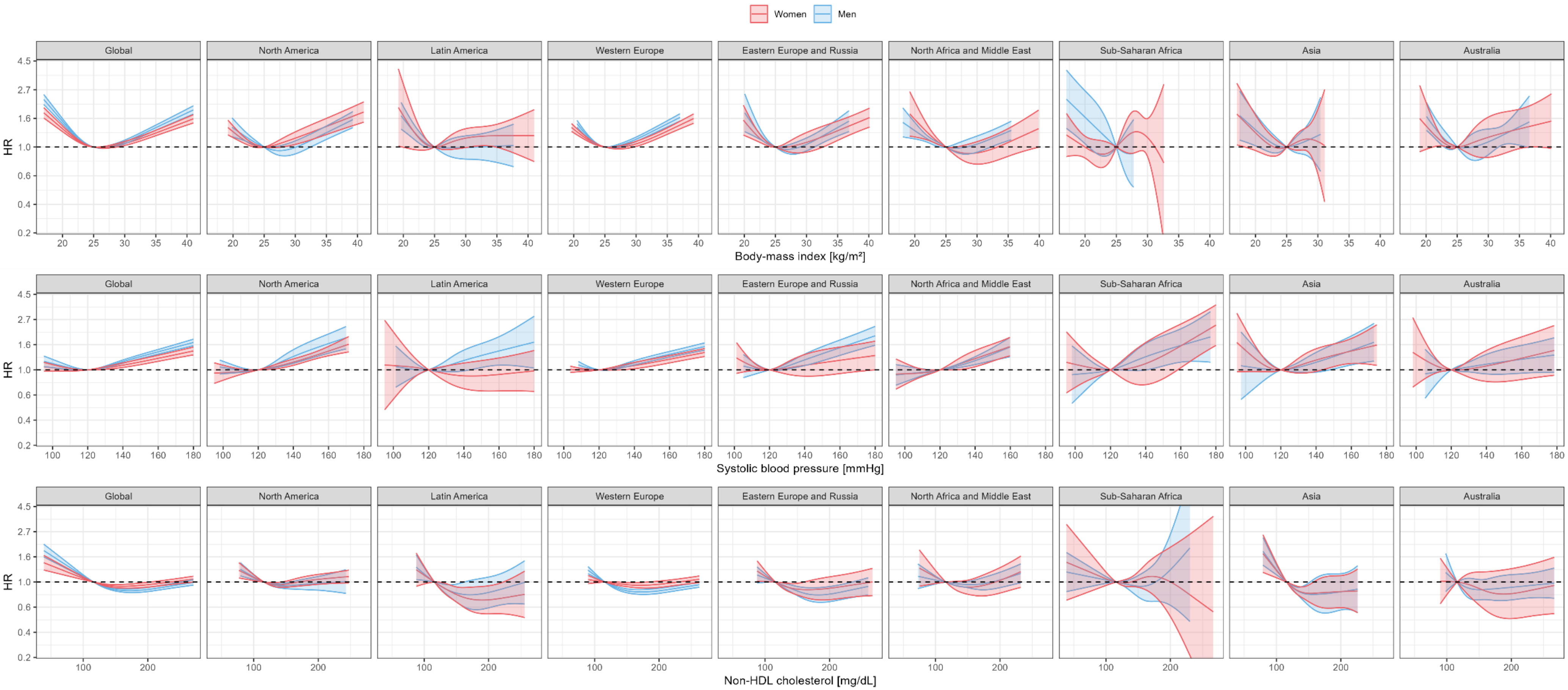

Figure S5. Associations of continuous risk factors with a) cardiovascular disease and b) all-cause mortality allowing for non-linear effects by geographic region using a two-year landmark analysis.

Individuals with cardiovascular disease at baseline were excluded. Age was used as the time scale. All five risk factors considered were included in the models together with use of antihypertensive medications. Confidence interval widths have not been adjusted for multiplicity and should not be used in place of hypothesis testing. To convert the values for non-HDL cholesterol from milligrams per deciliter (mg/dL) to millimoles per liter (mmol/L), multiply by 0.02586.

a) Cardiovascular disease

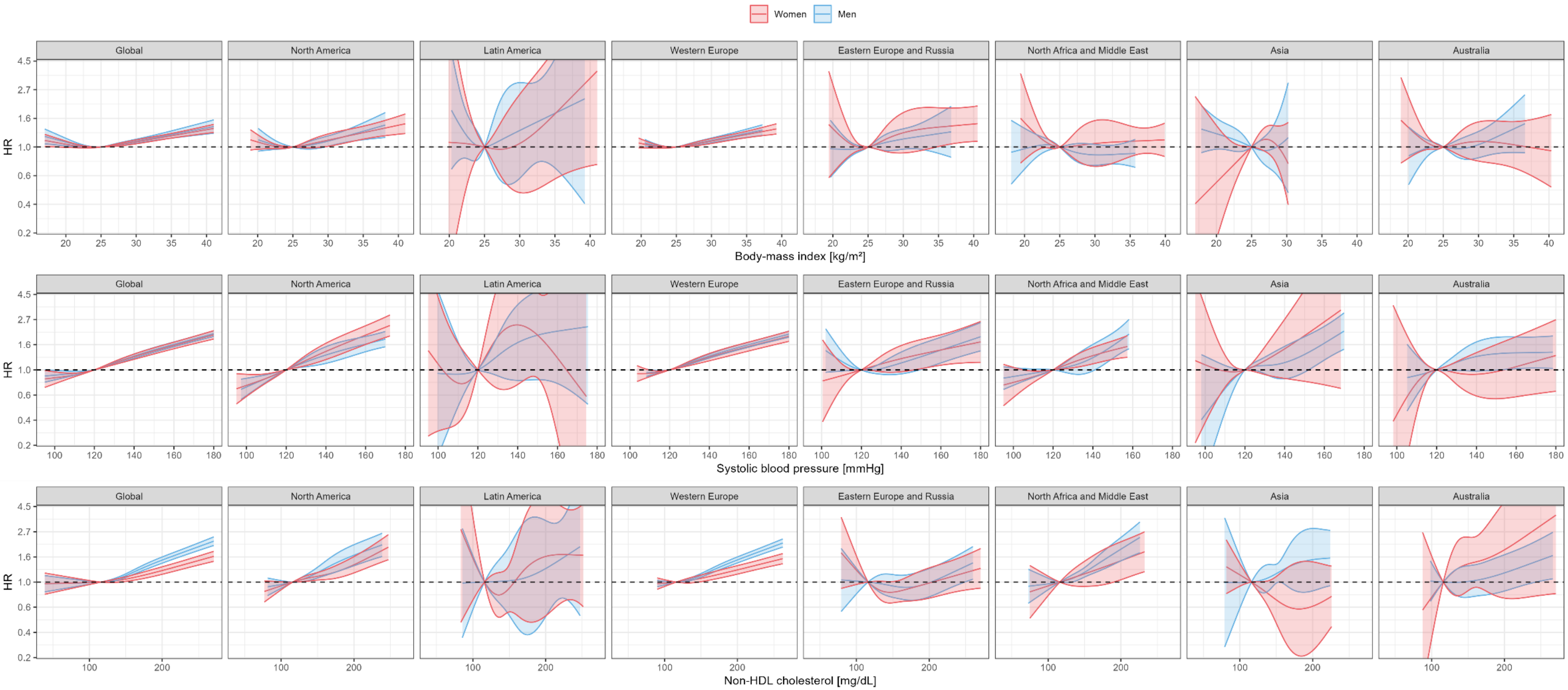

b) All-cause mortality

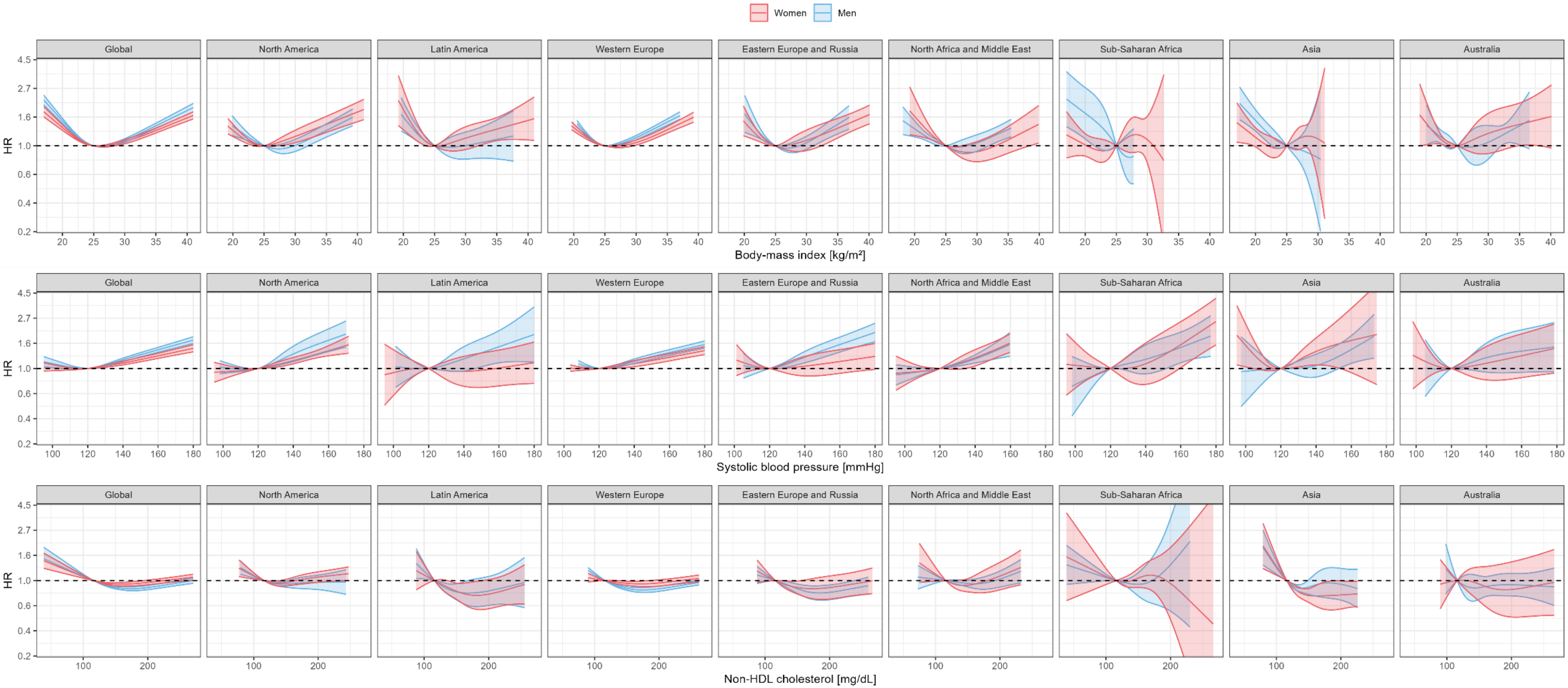

Figure S6. Associations of continuous risk factors with a) cardiovascular disease and b) all-cause mortality allowing for non-linear effects by geographic region and adjusting for lipid-lowering medications.

Individuals with cardiovascular disease at baseline were excluded. Age was used as the time scale. All five risk factors considered were included in the models together with use of antihypertensive medications and use of lipid-lowering medications. A one-year landmark analysis was performed. Confidence interval widths have not been adjusted for multiplicity and should not be used in place of hypothesis testing. To convert the values for non-HDL cholesterol from milligrams per deciliter (mg/dL) to millimoles per liter (mmol/L), multiply by 0.02586.

a) Cardiovascular disease

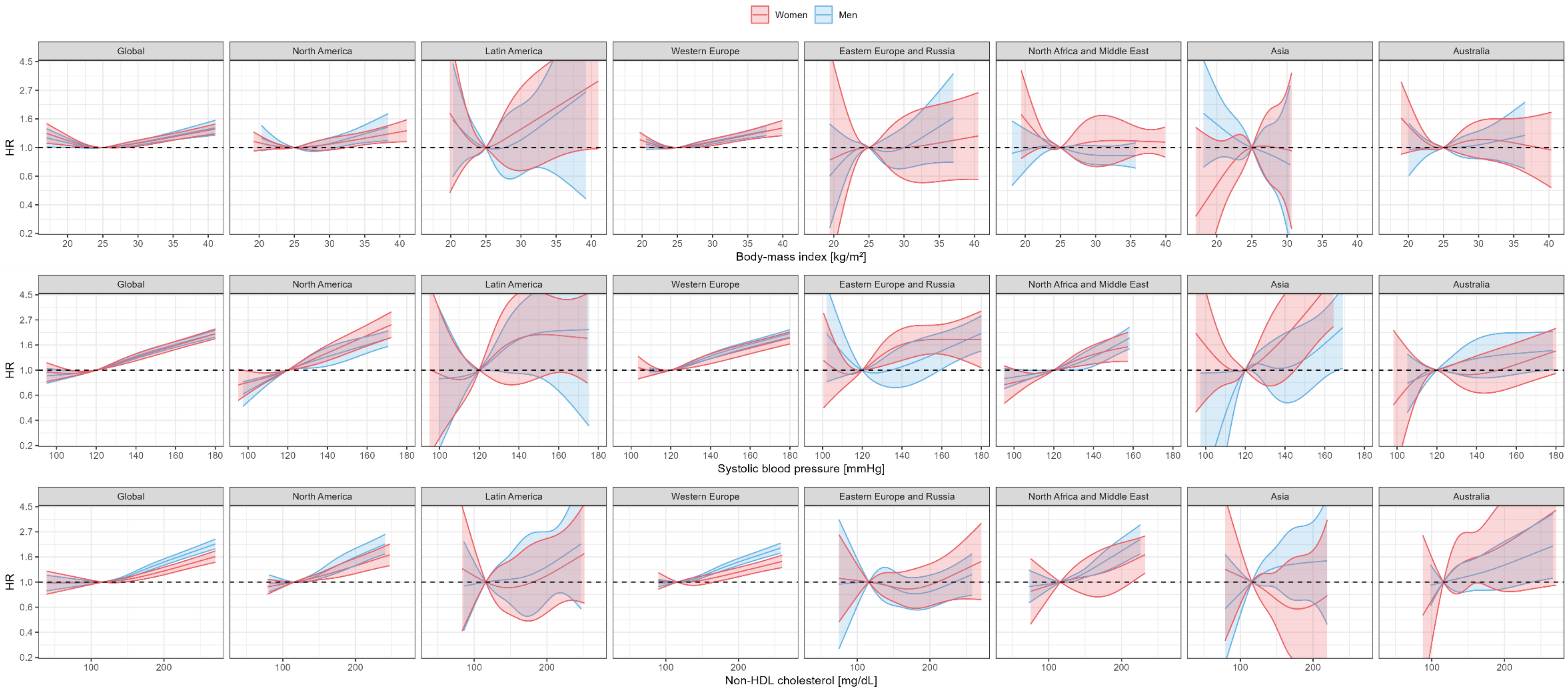

b) All-cause mortality

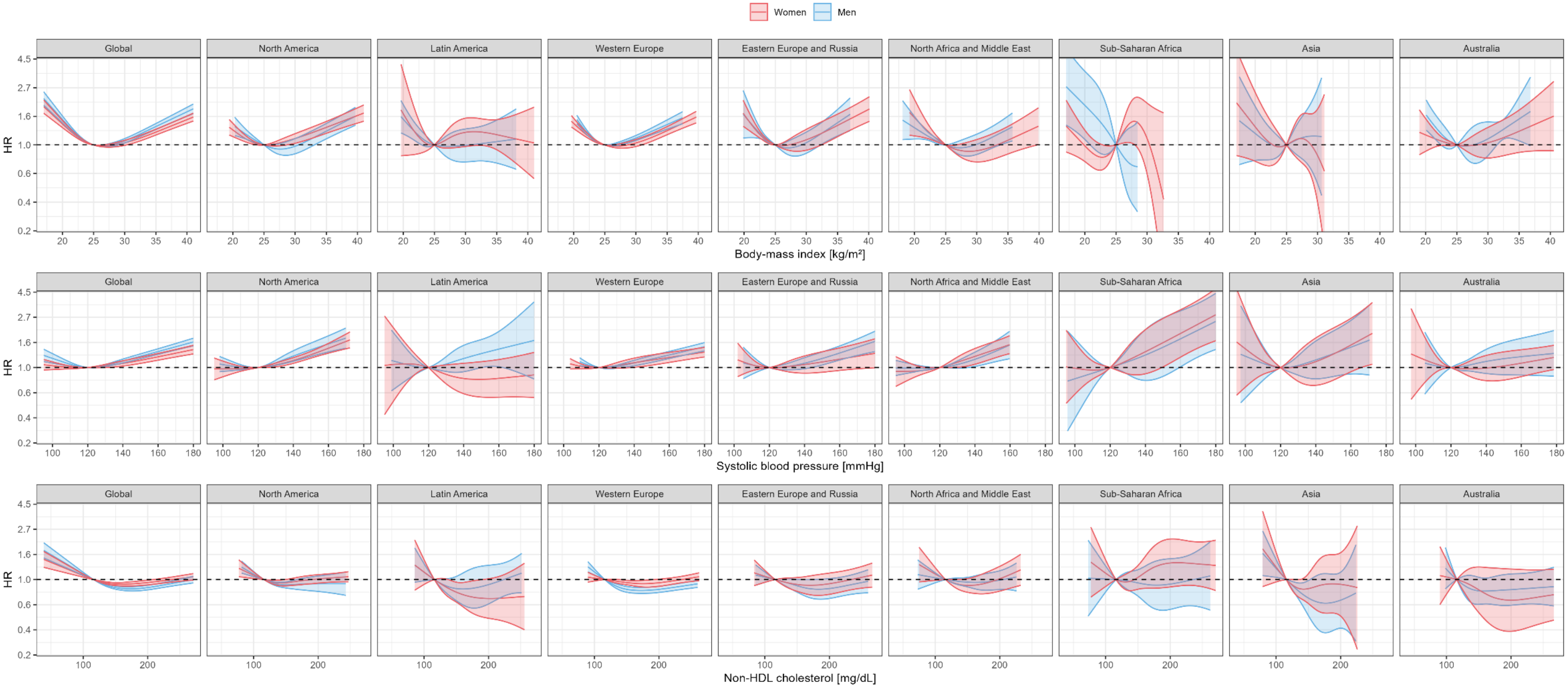

Figure S7. Associations of risk factors with a) cardiovascular disease and b) all-cause mortality allowing for effects to change with age by geographic region. *Individuals with cardiovascular disease at baseline were excluded. Age was used as the time scale. All five risk factors considered were included in the models together with the use of antihypertensive medications. A one-year landmark analysis was performed. Confidence interval widths have not been adjusted for multiplicity and should not be used in place of hypothesis testing. To convert the values for non-HDL cholesterol from milligrams per deciliter (mg/dL) to millimoles per liter (mmol/L), multiply by 0.02586.*

a) Cardiovascular disease

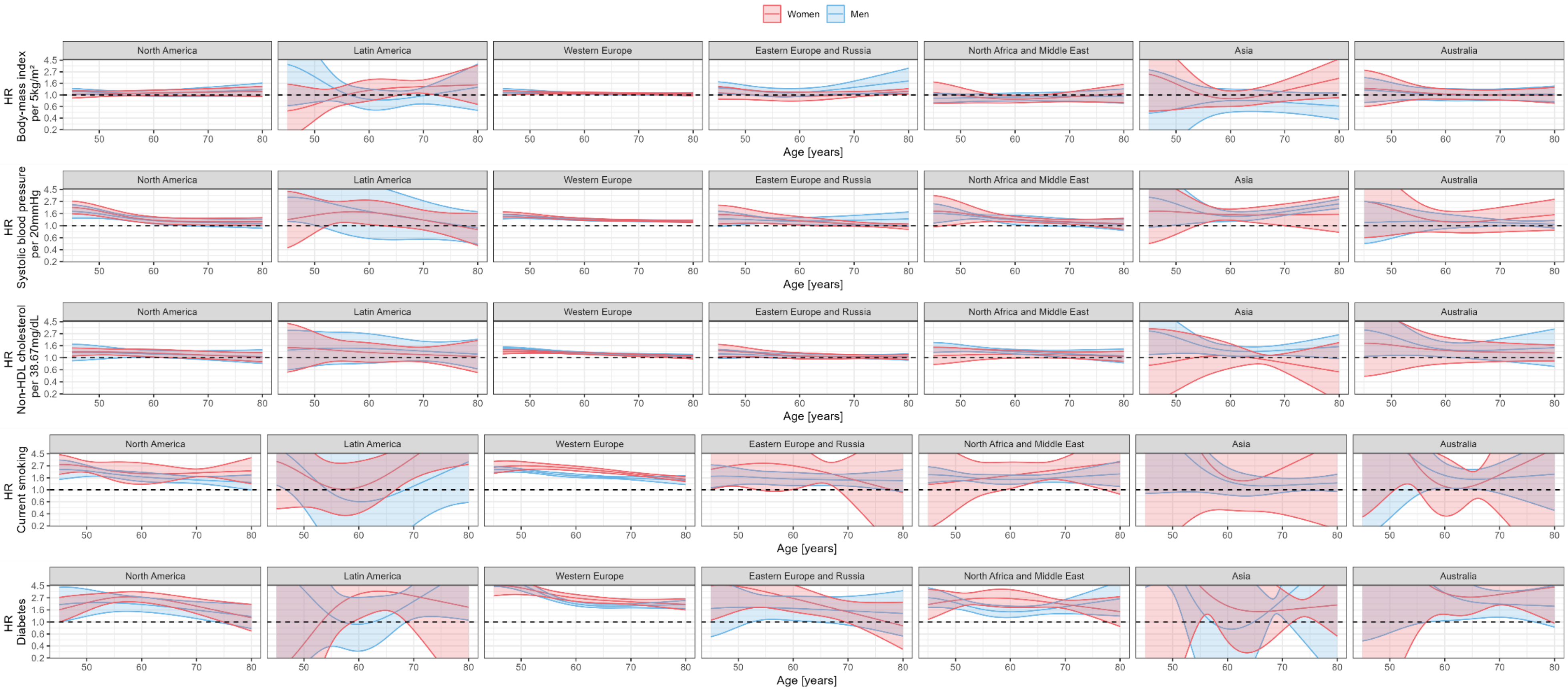

b) All- cause mortality

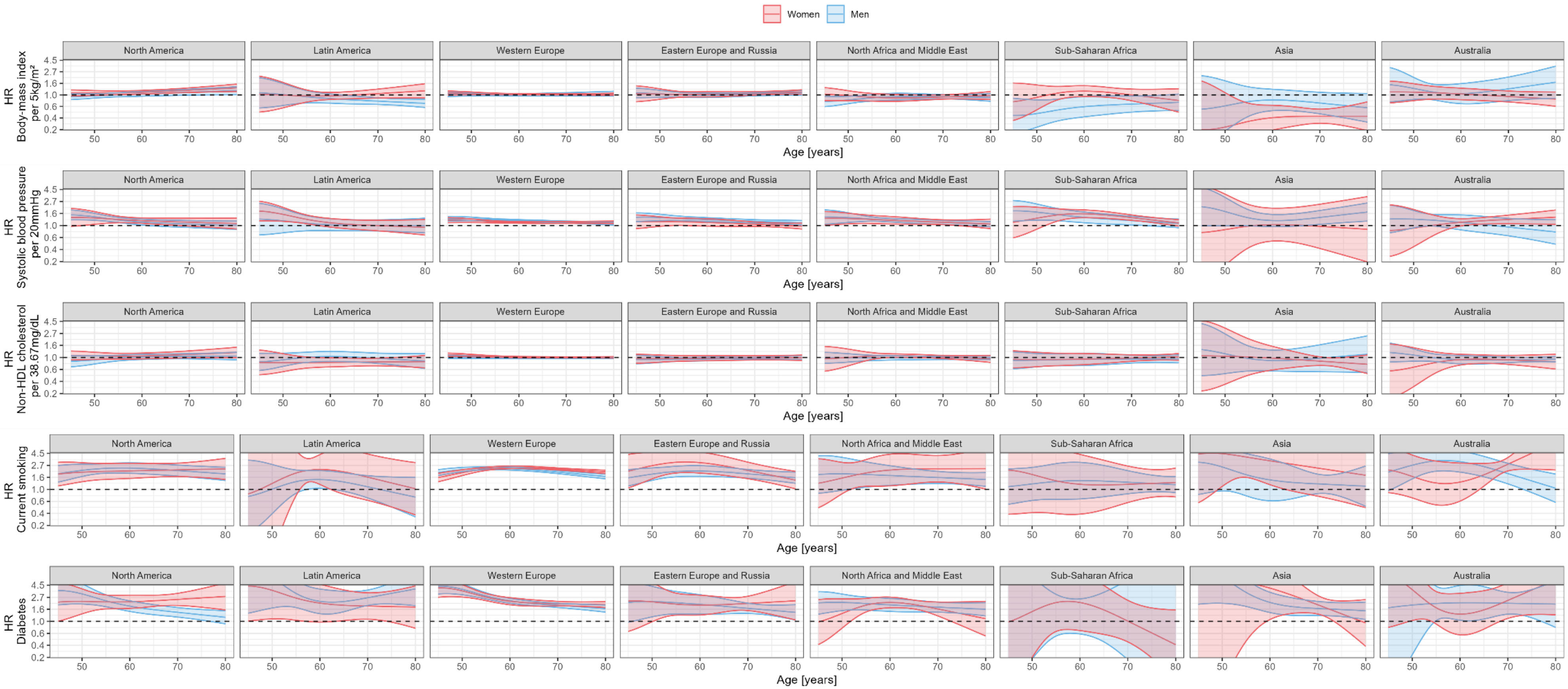

Figure S8. Graphical verification of distributional assumptions of Weibull models used for population-attributable fraction computations.

The estimated survival function of the standardized residuals obtained using the Kaplan-Meier estimator, and the survival function of the assumed distribution are shown for a selection of cohort studies from two geographic regions.

North America

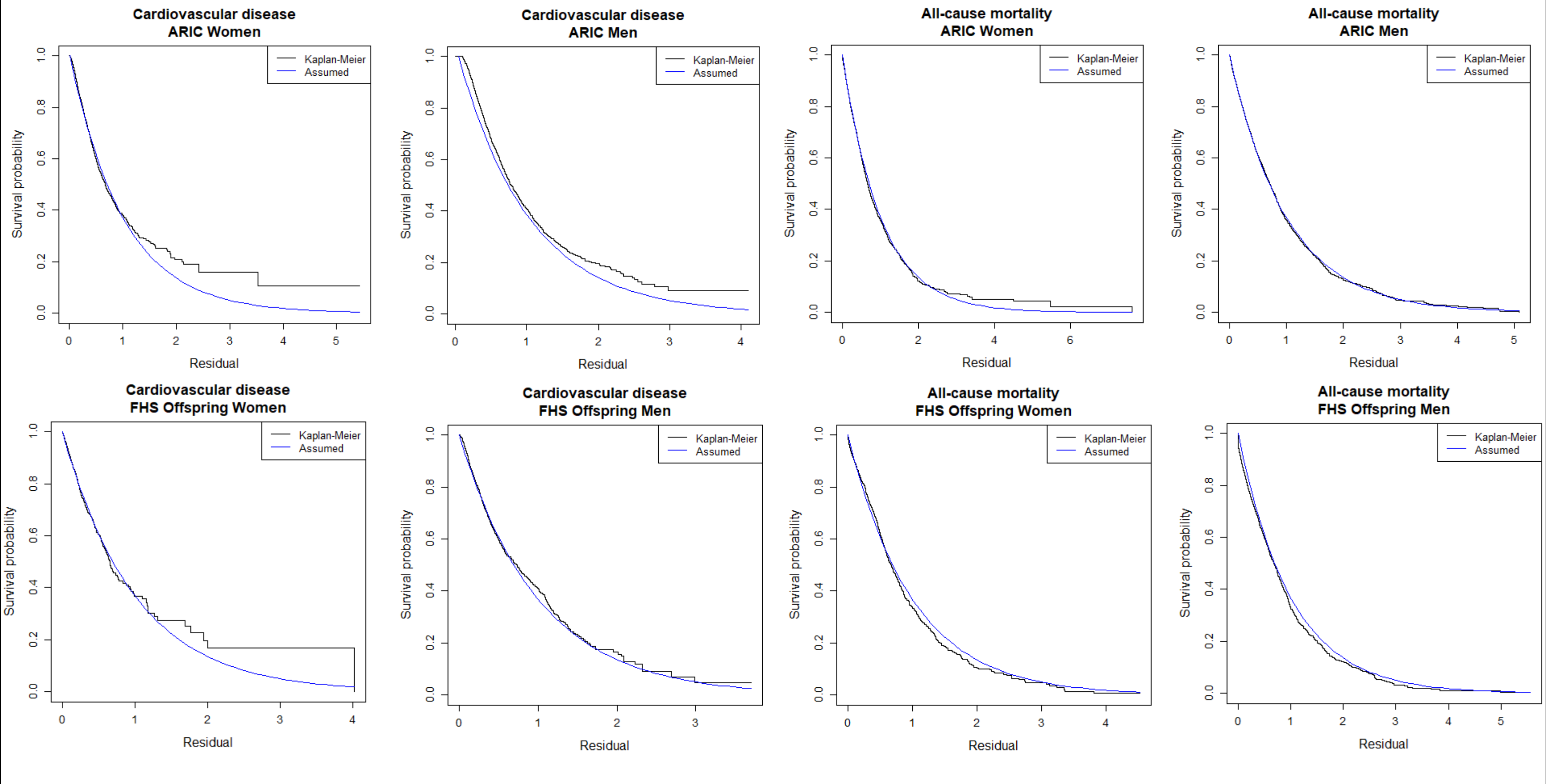

North America [continued]

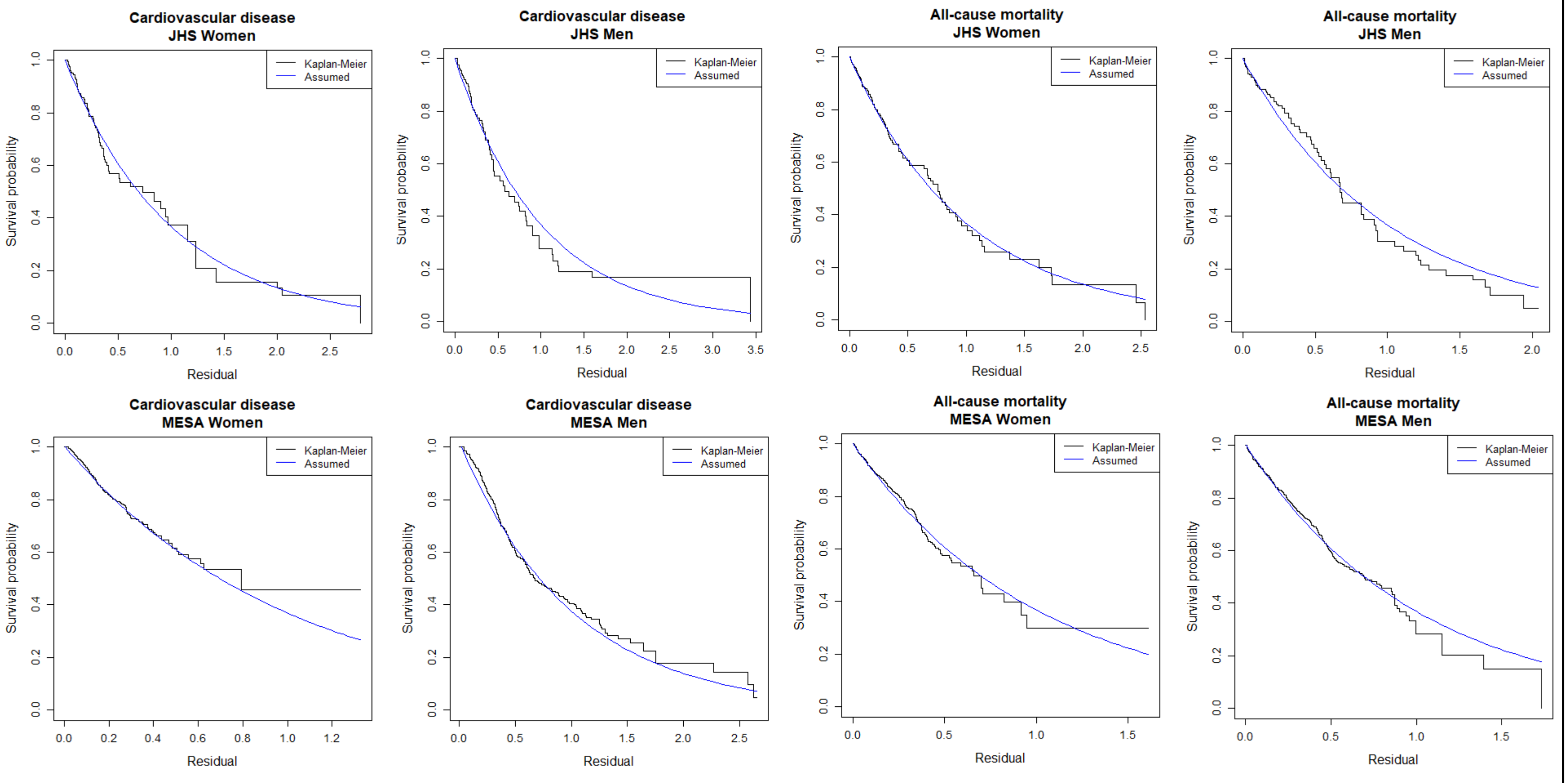

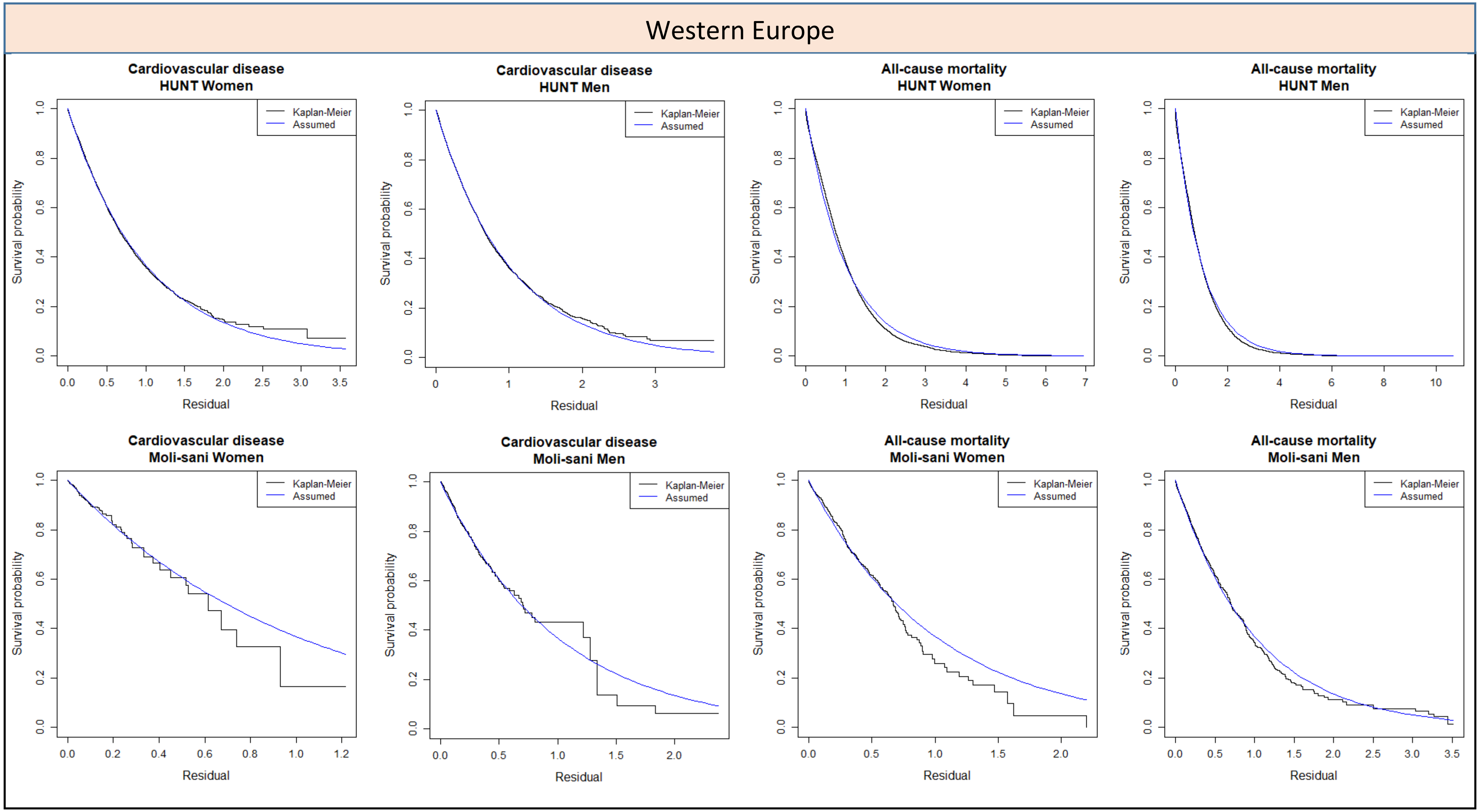
Western Europe
Cardiovascular disease
HUNT Women
Cardiovascular disease
HUNT Men
All-cause mortality
HUNT Women
All-cause mortality
HUNT Men
Cardiovascular disease
Moli-sani Women
Cardiovascular disease
Moli-sani Men
All-cause mortality
Moli-sani Women
All-cause mortality
Moli-sani Men
Kaplan-Meier
Assumed
Survival probability
Residual

Western Europe [continued]

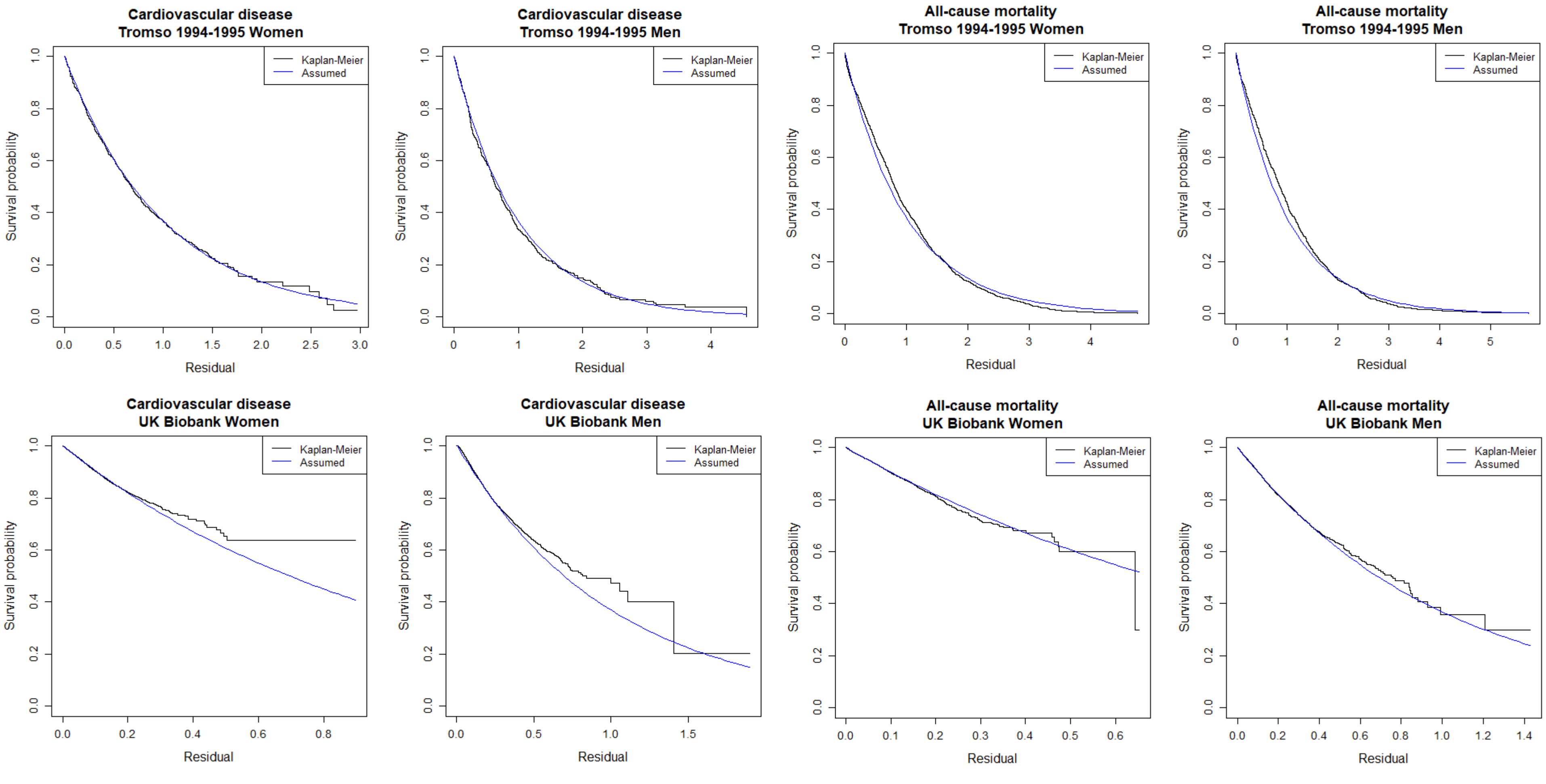

Supplementary Tables

**Table S1.** Variables of interest.	
**Variable**	**Description**
**Age**	Participant's age at baseline
**Sex**	Participant's sex
**Body-mass index**	Participant's measured body-mass index at baseline
**Smoking**	Self-reported daily or occasional tobacco consumption (including cigars, cigarillos or pipes)
**Systolic blood pressure**	Participant's measured systolic blood pressure
**Diastolic blood pressure**	Participant's measured diastolic blood pressure
**Antihypertensive drugs**	Intake of antihypertensive drugs (self-reported/medical records)
**Total cholesterol**	Participant's measured total cholesterol
**Non-HDL cholesterol**	Participant's derived non-HDL cholesterol
**HDL cholesterol**	Participant's measured HDL cholesterol
**Lipid-lowering drugs**	Intake of lipid-lowering drugs (self-reported/medical records)
**History of cardiovascular disease**	Any history of coronary heart disease, unstable angina, myocardial infarction, previous coronary revascularization or stroke at baseline (medical records)
**Incident cardiovascular disease**	First fatal or non-fatal myocardial infarction, unstable angina, coronary revascularization, ischemic and/or hemorrhagic stroke, and cardiovascular or unclassifiable death
**Incident cardiovascular disease (secondary end point)**	First fatal and non-fatal myocardial infarction, ischemic and/or hemorrhagic stroke, and cardiovascular death
**All-cause mortality**	Death from any cause

**Table S2.** End point definition.			
**Global Cardiovascular Risk Consortium end points**	**Definition and composition**	**Corresponding ICD code** ICD-10	ICD-9
**All-cause mortality**	Death from any cause	N/A	N/A
**Coronary heart disease (CHD)***	First possible or definite non-fatal CHD event	I20.0-I23	410-411
**CHD death***	Fatal CHD event, cardiac arrest or unclassifiable death	I20.0-I25, I46, R96, R98, R99	410-414, 798
**Stroke**	First possible or definite non-fatal stroke event	I61-I64, I69	431-434, 436, 438
**Stroke death**	Death due to cerebrovascular event	I61-I64, I69	431-434, 436, 438
**Revascularization**	First coronary revascularization	N/A	N/A
**Cardiovascular disease (CVD)**	First possible or definite CHD event, Possible or definite stroke event, Coronary revascularization, CHD death, Stroke death, Unclassifiable death		

* If it is not possible to distinguish I20.0 from I20, I20 was used. Coronary heart disease was defined as history of myocardial infarction, unstable angina, and coronary revascularization. N/A denotes not applicable.

Table S3. Background information and representativeness of the broader population affected by cardiovascular disease.

**Disease, problem, or condition under investigation**	Cardiovascular disease (CVD): first fatal or non-fatal myocardial infarction, unstable angina, coronary revascularization, ischemic or hemorrhagic stroke, and cardiovascular or unclassifiable death.<br>All-cause mortality: death from any cause.
**Special consideration related to**	
**Sex and gender**	Event rates of both CVD and all-cause mortality are higher in men than in women. Women in general have a more favorable risk factor profile than men. Global aggregate population-attributable fractions for CVD are higher in women than in men (for women: 57.2%, for men: 52.6%).
**Age**	The incidence of both CVD and all-cause mortality increases steeply with age.
**Race or ethnic group**	CVD prevalence is higher in Blacks than in Whites, Hispanics and Asians, while the difference seems to be more pronounced in women than in men.[18] Due to differing definitions of ethnicity in the different geographic regions, this variable is not analyzed in our study. In most cohorts, information on ethnicity is incompletely collected or not available in comparable standard.
**Geography**	Prevalence, event rates of CVD and all-cause mortality and impact of risk factors vary by geographic region.
**Other considerations**	The participants in the present study demonstrate the expected ratio of women to men. End points were carefully harmonized, but adjudication of causes of death might differ when analyzed using by healthcare systems.<br>Information on incident CVD was not available for sub-Saharan Africa.
**Overall representativeness of this trial**	Present analyses were performed for both sexes and eight geographic regions covering large parts of the world. The analyses were carried out on population-based cohorts with good participation rates. Generalizability of results within these regions is assumed.

Table S4. Cohort studies' data availability according to geographic region.

	Geographic regions							
	North America	Latin America	Western Europe	Eastern Europe and Russia	North Africa and Middle East	Sub-Saharan Africa	Asia	Australia
	no. participants (percentage of participants per total in region)							
**Survey years included**	35026 (53.7)	191244 (100)	907760 (100)	50417 (98.6)	185608 (100)	10390 (100)	59802 (100)	46909 (100)
**Examination age**	65182 (100)	191244 (100)	907760 (100)	51133 (100)	185608 (100)	10390 (100)	59802 (100)	46909 (100)
**Male/Female sex**	65182 (100)	191244 (100)	907760 (100)	51133 (100)	185608 (100)	10390 (100)	59802 (100)	46909 (100)
**Body-mass index**	64922 (99.6)	187472 (98.0)	901145 (99.3)	47477 (92.9)	184356 (99.3)	10120 (97.4)	53669 (89.7)	46644 (99.4)
**Systolic blood pressure**	65074 (99.8)	182300 (95.3)	873680 (96.2)	47992 (93.9)	184514 (99.4)	10355 (99.7)	54859 (91.7)	41621 (88.7)
**Diastolic blood pressure**	65020 (99.8)	182300 (95.3)	867017 (95.5)	31455 (61.5)	184509 (99.4)	10355 (99.7)	54834 (91.7)	41621 (88.7)
**Non-HDL cholesterol**	64177 (98.5)	21168 (11.1)	753329 (83.0)	41646 (81.4)	179633 (96.8)	9980 (96.1)	30957 (51.8)	46243 (98.6)
**HDL cholesterol**	64180 (98.5)	21169 (11.1)	753575 (83.0)	41675 (81.5)	179652 (96.8)	9994 (96.2)	36143 (60.4)	46256 (98.6)
**Current smoking**	64951 (99.6)	191071 (99.9)	899773 (99.1)	50861 (99.5)	184628 (99.5)	5863 (56.4)	59015 (98.7)	45805 (97.6)
**Diabetes**	64507 (99.0)	191173 (100.0)	895600 (98.7)	45674 (89.3)	184724 (99.5)	9848 (94.8)	58479 (97.8)	42430 (90.5)
**Antihypertensive medications**	64635 (99.2)	185477 (97.0)	865445 (95.3)	47086 (92.1)	184156 (99.2)	10387 (100.0)	40462 (67.7)	19825 (42.3)
**Lipid-lowering medications**	59545 (91.4)	178480 (93.3)	694183 (76.5)	34251 (67.0)	175659 (94.6)	0 (0)	37569 (62.8)	17324 (36.9)
**History of CVD**	64830 (99.5)	188719 (98.7)	891118 (98.2)	38897 (76.1)	184975 (99.7)	4524 (43.5)	58583 (98.0)	45742 (97.5)

Counts and percentages are given. HDL cholesterol, high-density lipoprotein cholesterol. Survey years included denotes the defined time range of baseline assessment. Examination age denotes information about the age of participants at the beginning of the study.

Table S5. Cohort studies' sex- and age-standardized selected baseline characteristics according to geographic region.

	Geographic regions								
	**Global**	**North America**	**Latin America**	**Western Europe**	**Eastern Europe and Russia**	**North Africa and Middle East**	**Sub-Saharan Africa**	**Asia**	**Australia**
**Participants — no.**	1,518,028	65,182	191,244	907,760	51,133	185,608	10,390	59,802	46,909
**Body-mass index — kg/m²**									
**< 20 %**	4.2	3.2	1.7	3.4	2.9	5.7	35.4	19.4	3.6
**20 - < 25 %**	32.3	28.6	19.7	35.1	27.6	26.9	47.3	53.9	33.0
**25 - < 30 %**	40.1	37.9	43.4	40.7	40.5	40.2	13.6	22.4	41.3
**≥ 30 %**	23.4	30.2	35.3	20.7	29.1	27.2	3.6	4.3	22.1
**Systolic blood pressure — mm Hg**									
**< 120 %**	28.0	44.1	28.1	20.2	22.6	55.2	38.2	41.4	31.9
**120 - < 140 %**	40.0	35.9	48.8	40.5	39.1	30.2	36.4	38.4	43.4
**140 - < 160 %**	22.1	14.4	17.2	26.6	24.5	10.6	14.9	14.7	18.7
**≥ 160 %**	9.9	5.6	5.8	12.6	13.8	4.0	10.5	5.5	6.0
**Non-HDL cholesterol — mg/dL**									
**< 116.01 %**	15.3	19.4	13.3	12.0	12.0	25.4	35.5	23.3	18.4
**116.01 - < 154.68 %**	32.6	35.1	35.1	30.3	30.6	38.8	34.3	41.3	34.7
**154.68 - < 193.35 %**	30.4	28.8	31.5	32.1	33.4	25.1	18.2	25.8	29.1
**≥ 193.35 %**	21.7	16.7	20.1	25.6	24.0	10.6	11.9	9.6	17.7
**HDL cholesterol — mg/dL**	52.1 (43.0, 63.0)	49.0 (39.8, 59.2)	41.0 (34.0, 49.1)	53.8 (44.7, 64.3)	51.4 (43.0, 62.0)	49.0 (41.0, 58.0)	38.7 (38.7, 69.2)	55.0 (44.9, 67.0)	55.3 (45.2, 66.9)

Percentages are used for categorical variables. Percentages and quartiles are based on available cases per variable. Percentages may not add up to 100 due to rounding. Quartiles and percentages per geographic region were computed using direct standardization according to the age and sex distribution of the Global Cardiovascular Risk Consortium dataset. For the standardization the following age groups were considered: age ≤40 years; 40< to ≤45; 45< to ≤50; 55< to ≤60; 65< to ≤70; and >70 years. Non-HDL cholesterol denotes non-high-density lipoprotein cholesterol, HDL-C high-density lipoprotein cholesterol. To convert the values for non-HDL cholesterol from milligrams per deciliter (mg/dL) to millimoles per liter (mmol/L), multiply by 0.02586.

Table S6. Cohort studies' baseline characteristics according to geographic region.

	Geographic regions							
	North America	Latin America	Western Europe	Eastern Europe and Russia	North Africa and Middle East	Sub-Saharan Africa	Asia	Australia
**N**	65182	191244	907760	51133	185608	10390	59802	46909
**N cohorts**	11	10	58	16	5	2	4	6
**Survey years included**	1971-2011	1990-2013	1970-2015	1983-2014	1963-2020	2011-2017	1988-2015	1983-2007
**Examination age — yr**	51.0 (40.0, 61.0)	52.0 (42.0, 63.0)	55.4 (46.6, 62.8)	54.0 (46.1, 61.1)	49.0 (42.0, 57.0)	38.0 (26.0, 52.0)	67.0 (58.0, 79.0)	62.2 (53.5, 70.9)
**Male sex — no. (%)**	28777 (44.1)	66580 (34.8)	436990 (48.1)	25784 (50.4)	88613 (47.7)	3741 (36.0)	26036 (43.5)	19849 (42.3)
**Body-mass index — kg/m²**	26.9 (23.8, 30.8)	28.2 (25.4, 31.6)	26.2 (23.7, 29.3)	27.2 (24.4, 30.5)	27.1 (24.1, 30.4)	21.0 (19.0, 23.4)	22.1 (19.6, 24.6)	26.5 (23.8, 29.6)
**Systolic blood pressure — mmHg**	120.0 (110.0, 134.0)	126.0 (116.7, 137.3)	134.5 (122.0, 148.0)	133.0 (120.0, 148.0)	113.5 (102.5, 125.0)	119.0 (110.0, 131.0)	129.0 (117.0, 143.0)	131.0 (120.0, 145.0)
**Diastolic blood pressure — mmHg**	73.0 (66.0, 80.0)	82.0 (76.7, 90.0)	81.0 (74.5, 89.0)	83.0 (77.2, 92.0)	74.0 (67.0, 80.0)	73.0 (67.0, 80.0)	77.0 (69.5, 84.0)	72.0 (64.0, 80.0)
**Non-HDL cholesterol — mg/dL**	146.0 (119.1, 175.9)	155.1 (129.2, 184.1)	163.4 (135.3, 194.1)	162.0 (134.6, 191.8)	139.2 (115.1, 165.9)	116.0 (77.3, 153.1)	138.4 (115.0, 163.6)	146.9 (119.9, 176.7)
**HDL cholesterol — mg/dL**	49.1 (40.4, 59.7)	42.1 (34.0, 49.9)	53.5 (44.5, 64.2)	51.4 (42.9, 62.0)	48.0 (40.0, 57.0)	38.7 (38.7, 66.5)	55.7 (45.2, 67.7)	56.8 (46.8, 68.8)
**Current smoking — no. (%)**	14759 (22.7)	54763 (28.7)	184028 (20.5)	15977 (31.4)	28848 (15.6)	635 (10.8)	11242 (19.0)	4847 (10.6)
**Diabetes — no. (%)**	7800 (12.1)	27347 (14.3)	43502 (4.9)	4165 (9.1)	28248 (15.3)	117 (1.2)	3251 (5.6)	2987 (7.0)
**Antihypertensive medications — no. (%)**	15667 (24.2)	35219 (19.0)	158389 (18.3)	13293 (28.2)	34649 (18.8)	1177 (11.3)	7111 (17.6)	4222 (21.3)
**Lipid-lowering medications — no. (%)**	4742 (7.3)	3508 (2.0)	99468 (12.3)	3747 (8.1)	17255 (9.3)	-	2811 (7.0)	1197 (6.0)
**History of CVD — no. (%)**	4315 (6.6)	6704 (3.5)	49468 (5.4)	5723 (11.2)	7101 (3.8)	0 (0)	4715 (7.9)	5356 (11.4)

Quartiles are presented for continuous variables, with the exception of survey years included where minimum and maximum are given. Percentages are used for binary variables.
Percentages and quartiles are based on available cases per variable. Percentages may not add up to 100 due to rounding.
HDL cholesterol, high-density lipoprotein cholesterol. To convert the values for non-HDL cholesterol from milligrams per deciliter (mg/dL) to millimoles per liter (mmol/L), multiply by 0.02586.

Table S7. Cohort studies' baseline characteristics according to geographic region and sex.

	North America		Latin America		Western Europe		Eastern Europe and Russia		North Africa and Middle East		Sub-Saharan Africa		Asia		Australia	
	Women	Men	Women	Men	Women	Men	Women	Men	Women	Men	Women	Men	Women	Men	Women	Men
N	36405	28777	124664	66580	470770	436990	25349	25784	96995	88613	6649	3741	33766	26036	27060	19849
Examination age — yr	51.0 (40.0, 61.0)	51.0 (39.0, 61.0)	51.0 (42.0, 63.0)	53.8 (43.0, 65.0)	55.5 (46.8, 62.8)	55.2 (46.3, 62.9)	54.0 (46.6, 61.6)	53.6 (46.0, 61.0)	48.8 (41.0, 56.1)	49.0 (42.0, 57.0)	37.4 (27.0, 51.1)	39.0 (25.0, 54.2)	66.0 (57.0, 81.0)	67.0 (58.0, 76.0)	62.4 (53.9, 71.0)	62.0 (53.0, 70.8)
Body-mass index — kg/m²	26.9 (23.3, 31.6)	27.0 (24.4, 30.0)	28.8 (25.7, 32.4)	27.4 (24.9, 30.1)	25.7 (23.0, 29.3)	26.7 (24.4, 29.3)	27.9 (24.5, 31.7)	26.8 (24.3, 29.6)	28.5 (25.3, 31.8)	25.9 (23.1, 28.7)	21.6 (19.5, 24.0)	20.4 (19.0, 22.0)	21.8 (19.3, 24.5)	22.4 (20.0, 24.8)	26.1 (23.2, 29.8)	26.9 (24.6, 29.4)
Systolic blood pressure — mmHg	118.0 (107.0, 134.0)	123.0 (113.0, 135.0)	125.3 (116.0, 136.7)	127.3 (119.3, 138.6)	131.5 (119.5, 146.5)	137.0 (126.0, 150.0)	130.0 (119.0, 146.5)	135.0 (122.5, 149.0)	111.0 (100.0, 122.5)	115.0 (105.0, 128.0)	117.0 (107.5, 129.0)	123.0 (114.0, 134.5)	128.0 (116.0, 142.0)	130.0 (119.0, 143.0)	130.0 (117.0, 144.0)	133.0 (122.5, 146.0)
Diastolic blood pressure — mmHg	71.3 (65.0, 79.0)	75.0 (69.0, 82.0)	81.3 (75.3, 89.3)	83.3 (78.0, 90.0)	80.0 (73.0, 87.0)	83.0 (76.5, 90.0)	82.0 (75.0, 90.0)	85.0 (79.0, 93.0)	72.0 (65.0, 80.0)	75.0 (69.0, 81.0)	73.0 (66.5, 80.0)	73.0 (67.0, 81.0)	76.0 (68.0, 83.0)	79.0 (71.0, 86.0)	68.0 (61.0, 76.0)	76.0 (69.0, 84.0)
Non-HDL cholesterol — mg/dL	143.0 (116.0, 173.2)	150.0 (123.4, 178.7)	155.1 (129.2, 184.8)	155.1 (129.2, 182.1)	161.6 (133.6, 193.0)	165.8 (137.1, 195.7)	161.2 (133.4, 191.8)	162.9 (135.3, 191.8)	139.1 (115.0, 165.9)	139.5 (115.2, 165.9)	116.0 (79.3, 154.7)	109.8 (77.3, 126.1)	141.9 (118.3, 166.7)	134.6 (111.4, 159.0)	144.6 (118.7, 174.0)	150.8 (122.6, 180.2)
HDL cholesterol — mg/dL	54.0 (44.9, 64.2)	44.0 (37.0, 51.8)	44.1 (37.1, 53.0)	37.9 (32.1, 46.0)	59.2 (50.3, 69.6)	47.6 (40.6, 56.1)	55.7 (46.8, 66.1)	47.6 (40.0, 57.0)	51.0 (43.7, 60.0)	44.1 (37.1, 52.0)	42.1 (38.7, 72.3)	38.7 (38.7, 53.4)	58.0 (47.6, 70.8)	52.2 (43.0, 63.0)	63.0 (52.6, 74.6)	49.5 (41.8, 59.2)
Antihypertensive medications — no. (%)	9297 (25.8)	6370 (22.3)	25513 (20.9)	9706 (15.3)	75558 (16.8)	82831 (19.9)	7224 (30.9)	6069 (25.6)	23067 (23.9)	11582 (13.2)	862 (13.0)	315 (8.4)	3819 (17.0)	3292 (18.3)	2421 (22.7)	1801 (19.7)
Lipid-lowering medications — no. (%)	2632 (7.3)	2110 (7.4)	2361 (2.0)	1147 (1.9)	39730 (9.5)	59738 (15.3)	2008 (8.8)	1739 (7.4)	11281 (11.6)	5974 (6.8)	-	-	1810 (8.1)	1001 (5.6)	618 (5.8)	579 (6.3)
History of CVD — no. (%)	1959 (5.4)	2356 (8.2)	3849 (3.1)	2855 (4.3)	16555 (3.5)	32913 (7.5)	2521 (9.9)	3202 (12.4)	3259 (3.4)	3842 (4.3)	0 (0)	0 (0)	2472 (7.3)	2243 (8.6)	2258 (8.3)	3098 (15.6)

Quartiles are presented for continuous variables. Counts and percentages are shown for binary variables. Percentages and quartiles are based on available cases per variable. HDL cholesterol, high-density lipoprotein cholesterol. To convert the values for non-HDL cholesterol from milligrams per deciliter (mg/dL) to millimoles per liter (mmol/L), multiply by 0.02586.

Table S8. Cohort studies' risk factor prevalence according to geographic region and sex.

	North America		Latin America		Western Europe		Eastern Europe and Russia		North Africa and Middle East		Sub-Saharan Africa		Asia		Australia	
	Women	Men	Women	Men	Women	Men	Women	Men	Women	Men	Women	Men	Women	Men	Women	Men
**N**	36405	28777	124664	66580	470770	436990	25349	25784	96995	88613	6649	3741	33766	26036	27060	19849
**Body-mass index**																
**< 20 kg/m² — no. (%)**	1875 (5.2)	632 (2.2)	2119 (1.7)	1300 (2.0)	21414 (4.6)	7312 (1.7)	548 (2.3)	555 (2.3)	3217 (3.3)	6730 (7.6)	1838 (28.6)	1368 (37.0)	9411 (31.0)	5745 (24.6)	1357 (5.0)	337 (1.7)
**20 - < 25 kg/m² — no. (%)**	11622 (32.1)	8088 (28.2)	22101 (18.1)	15327 (23.5)	181331 (38.8)	128863 (29.7)	6099 (26.0)	7101 (29.6)	19032 (19.8)	29466 (33.5)	3178 (49.5)	2024 (54.7)	14322 (47.2)	12177 (52.1)	9520 (35.4)	5314 (26.9)
**25 - < 30 kg/m² — no. (%)**	11019 (30.4)	12717 (44.3)	48425 (39.6)	31244 (48.0)	164179 (35.1)	208272 (48.0)	8526 (36.3)	11035 (46.0)	37598 (39.0)	37437 (42.5)	1061 (16.5)	278 (7.5)	5532 (18.2)	4856 (20.8)	9582 (35.6)	9969 (50.5)
**≥ 30 kg/m² — no. (%)**	11715 (32.3)	7254 (25.3)	49728 (40.6)	17228 (26.5)	100444 (21.5)	89330 (20.6)	8290 (35.3)	5323 (22.2)	36489 (37.9)	14387 (16.3)	341 (5.3)	32 (0.9)	1050 (3.5)	576 (2.5)	6426 (23.9)	4139 (20.9)
**Systolic blood pressure**																
**< 120 mmHg — no. (%)**	18921 (52.1)	11899 (41.4)	39463 (32.9)	15917 (25.6)	114236 (25.3)	54302 (12.9)	6159 (25.9)	4161 (17.2)	62441 (64.7)	49808 (56.6)	3780 (57.0)	1483 (39.8)	9915 (31.8)	6216 (26.2)	6936 (29.3)	3369 (18.8)
**120 - < 140 mmHg — no. (%)**	10872 (29.9)	11506 (40.0)	54706 (45.5)	31986 (51.4)	176756 (39.1)	180448 (42.8)	9120 (38.4)	9947 (41.1)	24607 (25.5)	26927 (30.6)	1936 (29.2)	1588 (42.6)	12186 (39.1)	10064 (42.5)	9379 (39.6)	8060 (44.9)
**140 - < 160 mmHg — no. (%)**	4574 (12.6)	3920 (13.6)	19154 (15.9)	10724 (17.2)	108417 (24.0)	131091 (31.1)	5316 (22.4)	6742 (27.8)	7236 (7.5)	8286 (9.4)	525 (7.9)	452 (12.1)	6056 (19.4)	5209 (22.0)	5156 (21.8)	4731 (26.4)
**≥ 160 mmHg — no. (%)**	1976 (5.4)	1406 (4.9)	6790 (5.7)	3560 (5.7)	52822 (11.7)	55608 (13.2)	3170 (13.3)	3377 (13.9)	2231 (2.3)	2978 (3.4)	390 (5.9)	201 (5.4)	3000 (9.6)	2213 (9.3)	2214 (9.3)	1776 (9.9)
**Non-HDL cholesterol**																
**< 116.01 mg/dL— no. (%)**	8712 (24.4)	5474 (19.3)	1775 (14.6)	1293 (14.3)	47641 (12.2)	40399 (11.1)	2657 (12.9)	2457 (11.7)	24717 (25.8)	21479 (25.6)	2248 (35.3)	1881 (52.0)	3797 (22.4)	4102 (29.4)	6009 (22.6)	4000 (20.4)
**116.01 - < 154.68 mg/dL— no. (%)**	12970 (36.3)	10077 (35.5)	4273 (35.3)	3211 (35.5)	122911 (31.5)	103918 (28.7)	6348 (30.9)	6405 (30.4)	38011 (39.7)	33369 (39.7)	2269 (35.7)	1131 (31.3)	7017 (41.3)	5806 (41.5)	9840 (37.0)	6547 (33.4)

Table S8 [continued].

**154.68 - < 193.35 mg/dL— no. (%)**	8981 (25.1)	8425 (29.6)	3671 (30.3)	2913 (32.2)	123236 (31.5)	121314 (33.5)	6604 (32.1)	7160 (34.0)	23411 (24.5)	21043 (25.1)	1122 (17.6)	407 (11.2)	4521 (26.6)	3106 (22.2)	6882 (25.8)	5638 (28.7)
**≥ 193.35 mg/dL— no. (%)**	5091 (14.2)	4447 (15.6)	2401 (19.8)	1631 (18.0)	96976 (24.8)	96934 (26.7)	4956 (24.1)	5059 (24.0)	9542 (10.0)	8061 (9.6)	722 (11.4)	200 (5.5)	1647 (9.7)	961 (6.9)	3897 (14.6)	3430 (17.5)
**Current smoking — no. (%)**	7527 (20.7)	7232 (25.3)	26046 (20.9)	28717 (43.2)	79822 (17.1)	104206 (24.0)	4768 (18.9)	11209 (43.7)	2530 (2.6)	26318 (29.9)	86 (2.5)	549 (22.7)	1772 (5.3)	9470 (37.3)	2296 (8.7)	2551 (13.2)
**Diabetes — no. (%)**	4308 (12.0)	3492 (12.2)	17969 (14.4)	9378 (14.1)	18125 (3.9)	25377 (5.9)	2056 (8.8)	2109 (9.4)	17202 (17.8)	11046 (12.5)	67 (1.1)	50 (1.4)	1532 (4.7)	1719 (6.7)	1481 (6.1)	1506 (8.3)

Counts and percentages are presented. Percentages are based on available cases per variable. Percentages may not add up to 100 due to rounding. HDL cholesterol, high-density lipoprotein cholesterol. To convert the values for non-HDL cholesterol from milligrams per deciliter (mg/dL) to millimoles per liter (mmol/L), multiply by 0.02586.

Table S9. Health examination surveys' baseline characteristics by survey.

Survey	N	Examination age *yr*	Male sex *%*	Body-mass index *kg/m²*	Systolic blood pressure *mmHg*	Diastolic blood pressure *mmHg*	Non-HDL cholesterol *mg/dL*	HDL cholesterol *mg/dL*	Current smoking *%*	Diabetes *%*	Anti-hypertensive medications *%*	Lipid-lowering medications *%*	History of CVD *%*
**NHANES**	5856	47.0 (32.0, 61.0)	48.2	28.5 (24.5, 33.5)	120.0 (111.0, 133.0)	72.0 (65.0, 79.0)	130.7 (105.2, 158.9)	51.0 (42.1, 61.9)	18.7	11.2	23.8	40.0	8.6
**ENS**	3691	43.0 (30.0, 57.0)	49.0	28.2 (25.3, 31.6)	120.5 (110.0, 132.5)	74.0 (67.0, 81.0)	128.0 (102.9, 155.1)	44.9 (37.9, 54.9)	35.4	12.5	17.4	7.9	6.1
**ENSANUT**	17263	42.0 (30.0, 56.0)	45.3	28.2 (25.0, 31.7)	121.0 (110.0, 133.0)	75.0 (68.0, 82.0)	139.2 (116.0, 164.0)	42.9 (37.1, 49.9)	17.7	10.3	12.9	6.7	2.5
**PNS**	8946	43.0 (31.0, 57.0)	47.1	25.9 (22.8, 29.4)	123.0 (113.0, 135.0)	77.5 (70.5, 85.0)	135.0 (111.0, 162.0)	44.9 (37.9, 54.1)	14.8	7.5	19.2	11.0	3.6
**WHO STEPS Guyana**	1178	35.0 (25.0, 48.0)	50.5	25.2 (21.9, 30.0)	123.0 (114.0, 136.5)	76.5 (69.5, 85.5)	140.0 (110.0, 169.0)	47.0 (37.0, 60.0)	13.7	19.2	13.5	16.0	9.2
**DEGS1**	7115	47.0 (34.0, 61.0)	49.7	26.2 (23.2, 29.8)	123.0 (113.5, 133.0)	72.5 (67.5, 78.5)	143.5 (117.6, 173.6)	53.0 (45.2, 63.0)	23.7	7.2	22.9	8.7	5.4
**FinHealth 2017**	5698	50.5 (34.9, 65.4)	49.2	26.6 (23.7, 30.1)	127.0 (117.0, 141.0)	77.0 (70.0, 85.0)	137.7 (112.5, 165.5)	55.3 (46.8, 65.7)	21.8	10.2	23.8	18.5	7.2
**HCHS**	10000	58.0 (49.0, 70.0)	47.0	26.0 (23.4, 29.0)	135.5 (123.5, 149.0)	81.5 (75.0, 88.0)	140.8 (114.1, 169.0)	61.9 (49.9, 76.2)	18.7	5.3	29.8	15.3	7.0
**KYH**	4504	55.2 (46.4, 63.1)	41.9	27.5 (24.2, 31.2)	130.0 (118.5, 144.5)	82.5 (75.0, 90.5)	154.7 (128.0, 184.5)	54.1 (46.0, 63.8)	24.9	7.8	33.8	57.5	10.9
**WHO STEPS Azerbaijan**	2688	38.0 (28.0, 51.0)	49.0	25.8 (22.9, 29.3)	122.5 (113.5, 134.0)	80.5 (73.5, 88.0)	124.9 (97.4, 152.8)	41.4 (35.2, 49.9)	23.7	16.5	15.4	7.1	6.3
**WHO STEPS Belarus**	5010	43.0 (30.0, 55.0)	47.6	26.4 (23.1, 30.1)	130.0 (120.5, 145.5)	83.0 (77.0, 92.0)	126.1 (101.7, 153.5)	50.3 (42.1, 61.9)	29.6	6.4	20.3	3.5	6.7
**WHO STEPS Georgia**	3243	42.0 (30.0, 55.0)	48.0	27.4 (23.8, 32.0)	125.5 (116.0, 140.0)	81.0 (74.0, 90.0)	121.8 (93.2, 158.2)	35.6 (27.5, 44.9)	29.6	14.0	20.8	10.5	21.4
**WHO STEPS Tajikistan**	2717	28.0 (24.0, 34.0)	51.9	24.6 (22.0, 27.6)	125.5 (117.0, 136.5)	83.0 (77.0, 90.0)	95.9 (76.6, 123.4)	40.2 (34.0, 49.5)	4.8	13.1	6.7	5.0	2.7
**WHO STEPS Turkmenistan**	4053	33.0 (26.0, 48.0)	51.1	24.8 (22.4, 28.0)	124.0 (118.0, 132.5)	81.5 (76.5, 87.0)	107.1 (80.0, 133.0)	50.3 (41.4, 61.9)	3.5	2.8	8.8	1.6	5.9

Table S9 [continued].													
**WHO STEPS Iraq**	4060	31.0 (23.0, 46.0)	51.9	27.4 (23.5, 31.8)	125.0 (119.5, 138.5)	80.5 (77.5, 90.0)	129.0 (100.0, 164.0)	43.0 (36.5, 52.0)	20.7	21.3	16.7	25.4	4.4
**WHO STEPS Jordan**	3643	35.0 (25.0, 45.0)	50.3	27.1 (22.9, 31.7)	114.5 (105.0, 125.5)	77.0 (71.0, 85.0)	97.8 (76.2, 127.6)	38.3 (31.7, 46.0)	40.3	23.7	17.4	23.0	8.3
**WHO STEPS Lebanon**	1208	40.0 (32.0, 51.0)	48.5	27.0 (24.0, 30.8)	124.0 (114.0, 138.0)	75.0 (70.0, 82.5)	155.0 (127.0, 187.0)	46.0 (39.0, 55.0)	37.0	18.2	17.9	23.2	5.9
**WHO STEPS Lebanon (Syrian)**	1261	36.0 (29.0, 43.0)	42.6	27.1 (23.9, 31.0)	125.0 (116.0, 138.0)	79.0 (70.0, 83.5)	142.0 (117.0, 170.0)	44.0 (38.0, 53.0)	27.0	34.6	13.8	30.3	3.6
**WHO STEPS Morocco**	4884	39.0 (28.0, 53.0)	49.1	25.5 (22.0, 29.1)	126.5 (116.5, 137.5)	77.0 (70.5, 84.5)	88.0 (71.0, 115.0)	42.0 (34.0, 51.0)	11.0	19.7	9.8	11.1	3.4
**WHO STEPS Sudan**	7273	31.0 (23.0, 43.0)	54.7	21.8 (19.2, 25.7)	125.0 (116.0, 136.0)	82.0 (76.0, 89.0)	103.6 (84.7, 132.6)	30.9 (22.4, 41.4)	9.7	25.4	8.9	12.3	1.2
**SANHANES**	5014	36.0 (26.0, 49.0)	41.6	26.2 (21.8, 31.5)	127.0 (115.0, 140.0)	75.0 (67.0, 84.0)	117.6 (93.2, 146.9)	46.4 (38.7, 58.0)	19.5	7.3	13.2	2.9	6.6
**WHO STEPS Eswatini**	3259	29.0 (22.0, 41.0)	44.0	24.7 (21.6, 29.4)	120.5 (112.5, 133.0)	79.0 (72.0, 87.0)	90.1 (69.6, 116.8)	44.1 (36.0, 55.3)	6.8	12.8	9.4	11.8	4.5
**WHO STEPS Ethiopia**	9251	29.0 (24.0, 40.0)	54.0	20.1 (18.6, 21.9)	118.0 (109.5, 128.0)	78.0 (71.0, 84.5)	84.0 (72.0, 108.0)	38.0 (31.0, 48.0)	4.6	19.6	3.7	5.3	3.5
**WHO STEPS Bangladesh**	7199	37.0 (28.0, 48.0)	45.5	22.3 (19.6, 25.4)	117.5 (109.0, 127.5)	78.0 (71.0, 85.5)	128.0 (105.0, 154.0)	38.0 (33.0, 44.0)	23.0	21.2	8.9	18.6	11.0
**WHO STEPS Bhutan**	5450	34.0 (24.0, 45.0)	54.4	24.5 (21.9, 27.4)	122.5 (113.5, 132.5)	81.0 (74.5, 89.0)	92.0 (72.0, 119.0)	39.0 (33.0, 47.0)	10.6	9.4	7.0	6.8	3.6
**WHO STEPS Myanmar**	8271	41.0 (32.0, 50.0)	50.9	22.0 (19.5, 25.1)	122.5 (112.5, 134.5)	81.0 (73.5, 88.5)	127.0 (102.0, 155.0)	45.0 (38.0, 53.0)	26.3	23.6	11.9	11.6	6.3
**AusDiab**	11247	49.4 (36.3, 63.9)	49.0	26.2 (23.4, 29.4)	127.0 (116.0, 140.0)	69.5 (61.5, 77.0)	159.3 (132.2, 187.6)	52.6 (44.1, 63.4)	16.5	5.0	15.5	8.4	6.0

Quartiles are presented for continuous variables and percentages are used for binary variables. Percentages and quartiles are based on available cases per variable. Estimates are weighted if sampling weights were available. HDL cholesterol, high-density lipoprotein cholesterol. To convert the values for non-HDL cholesterol from milligrams per deciliter (mg/dL) to millimoles per liter (mmol/L), multiply by 0.02586.

Table S10. Health examination surveys' baseline characteristics according to geographic region.

	Geographic regions								
	Global	North America	Latin America	Western Europe	Eastern Europe and Russia	North Africa and Middle East	Sub-Saharan Africa	Asia	Australia
**N**	153982	5856	31078	22813	22215	22329	17524	20920	11247
**Survey years included**	1999-2019	2017-2018	2014-2019	2008-2017	2015-2018	2015-2019	2012-2015	2014-2019	1999-2000
**Examination age — yr**	42.0 (30.0, 56.0)	47.0 (32.0, 61.0)	43.0 (31.0, 57.0)	48.0 (34.0, 61.0)	36.0 (26.0, 50.0)	32.0 (24.0, 45.0)	32.0 (25.0, 45.0)	38.0 (30.0, 49.0)	49.4 (36.3, 63.9)
**Male sex — %**	47.9	48.2	46.6	49.6	49.5	52.7	47.5	47.2	49.0
**Body-mass index — kg/m²**	25.9 (22.1, 30.1)	28.5 (24.5, 33.5)	26.8 (23.5, 30.4)	26.2 (23.3, 29.8)	25.6 (22.7, 29.1)	24.7 (20.8, 29.4)	22.0 (19.5, 27.2)	22.2 (19.6, 25.3)	26.2 (23.4, 29.4)
**Systolic blood pressure — mmHg**	121.5 (111.5, 133.0)	120.0 (111.0, 133.0)	122.0 (112.0, 134.0)	123.5 (114.0, 133.5)	125.5 (117.0, 138.0)	123.5 (115.0, 135.5)	122.0 (112.0, 134.0)	119.0 (110.0, 130.0)	127.0 (116.0, 140.0)
**Diastolic blood pressure — mmHg**	75.5 (68.5, 83.5)	72.0 (65.0, 79.0)	76.5 (69.5, 84.0)	72.5 (67.5, 79.0)	82.0 (76.0, 90.0)	80.5 (75.0, 88.5)	76.5 (69.0, 84.0)	79.0 (71.5, 86.5)	69.5 (61.5, 77.0)
**Non-HDL cholesterol — mg/dL**	130.7 (104.4, 159.0)	130.7 (105.2, 158.9)	136.1 (112.1, 162.0)	143.5 (117.6, 172.8)	116.4 (88.9, 145.4)	115.6 (86.6, 149.3)	100.5 (77.3, 131.5)	128.0 (104.0, 154.0)	159.3 (132.2, 187.6)
**HDL cholesterol — mg/L**	46.0 (37.9, 56.0)	51.0 (42.1, 61.9)	44.1 (37.9, 53.0)	53.4 (45.2, 63.4)	44.5 (36.4, 54.1)	38.0 (29.0, 48.0)	42.5 (34.0, 53.4)	40.0 (34.0, 47.0)	52.6 (44.1, 63.4)
**Current smoking — %**	19.1	18.7	16.9	23.6	18.7	18.9	11.9	24.0	16.5
**Diabetes — %**	10.4	11.2	8.8	7.3	8.5	22.5	7.7	21.6	5.0
**Antihypertensive medications — %**	18.0	23.8	16.9	23.0	14.8	14.3	11.3	9.8	15.5
**Lipid-lowering medications — %**	16.1	40.0	9.2	9.3	3.9	22.6	2.9	17.2	8.4
**History of CVD — %**	6.5	8.6	3.4	5.5	6.5	3.4	5.1	9.5	6.0

Quartiles are presented for continuous variables, with the exception of survey years included where minimum and maximum are presented. Percentages are presented for binary variables. Percentages and quartiles are based on available cases per variable. Quartiles and percentages are weighted using sampling weights. HDL cholesterol, high-density lipoprotein cholesterol. To convert the values for non-HDL cholesterol from milligrams per deciliter (mg/dL) to millimoles per liter (mmol/L), multiply by 0.02586.

Table S11. Health examination surveys' risk factor prevalence according to geographic region and sex.

	Geographic regions															
	North America		Latin America		Western Europe		Eastern Europe and Russia		North Africa and Middle East		Sub-Saharan Africa		Asia		Australia	
	Women	Men	Women	Men	Women	Men	Women	Men	Women	Men	Women	Men	Women	Men	Women	Men
**N**	3016	2840	18120	12958	11894	10919	13424	8791	14306	8023	10980	6544	12603	8317	6199	5048
**Body-mass index**																
**< 20 kg/m² — %**	6.2	4.0	6.2	5.8	6.9	2.5	9.3	6.1	15.9	22.7	23.0	39.0	24.1	34.9	6.3	2.4
**20 - < 25 kg/m² — %**	24.1	19.7	27.9	31.4	39.8	30.3	36.6	38.4	28.6	35.5	32.4	42.0	41.4	46.9	38.8	29.2
**25 - < 30 kg/m² — %**	27.9	34.2	34.5	40.6	29.3	43.9	29.1	39.1	26.0	24.9	18.5	12.4	26.0	15.5	32.1	48.2
**≥ 30 kg/m² — %**	41.8	42.1	31.4	22.3	24.0	23.3	25.0	16.4	29.5	17.0	26.1	6.6	8.6	2.7	22.7	20.1
**Systolic blood pressure**																
**< 120 mmHg — %**	53.5	42.5	52.0	33.7	52.2	28.8	40.1	23.7	42.4	26.6	48.5	40.6	55.9	49.4	43.5	22.8
**120 - < 140 mmHg — %**	29.8	41.8	32.0	46.4	34.7	53.6	38.5	52.2	38.5	52.3	33.3	39.8	28.9	37.5	34.4	48.2
**140 - < 160 mmHg — %**	11.6	12.5	10.6	14.1	11.2	15.4	13.4	16.6	12.9	14.8	12.0	14.0	10.0	8.5	14.8	20.9
**≥ 160 mmHg — %**	5.1	3.2	5.4	5.8	2.0	2.2	8.0	7.5	6.1	6.3	6.2	5.6	5.2	4.7	7.4	8.0
**Non-HDL cholesterol**																
**< 116.01 mg/dL — %**	35.4	34.9	28.9	28.2	26.5	20.7	48.7	50.2	46.6	53.1	57.8	70.4	37.1	36.2	15.8	10.0
**116.01 - < 154.68 mg/dL — %**	38.0	34.3	39.9	39.7	37.6	36.3	30.5	32.2	30.2	25.7	26.1	19.1	37.7	39.6	34.2	30.7

Table S11 [continued].

**154.68 - < 193.35 mg/dL — %**	19.1	21.6	23.2	23.9	24.0	27.5	14.5	13.1	14.9	13.9	11.0	7.3	18.4	18.8	31.2	34.9
**≥ 193.35 mg/dL — %**	7.6	9.2	8.0	8.1	11.9	15.5	6.2	4.6	8.3	7.3	5.1	3.2	6.8	5.5	18.8	24.4
**Current smoking — %**	15.9	21.7	11.2	23.5	21.3	25.9	4.2	33.5	5.3	31.1	5.9	18.6	2.8	47.7	14.6	18.4
**Diabetes — %**	9.9	12.6	9.9	7.5	7.4	7.2	9.3	7.5	20.8	24.5	9.1	5.9	21.9	21.3	4.4	5.7

Percentages are presented are based on available cases per variable. Percentages may not add up to 100 due to rounding. HDL cholesterol, high-density lipoprotein cholesterol. To convert the values for non-HDL cholesterol from milligrams per deciliter (mg/dL) to millimoles per liter (mmol/L), multiply by 0.02586.

Table S12. Cohort studies' follow-up information for cardiovascular disease.

Cohort	Survey years included Min-Max	Examination age *Yr* *Min-Max*	Age at end of follow-up *Yr* *Min-Max*	Follow-up time *Yr* *Min-Max*	Number of individuals with follow-up information	Number of events	Follow-up time *Yr* *Median (25th percentile, 75th percentile)*
All	1970-2020	18-101.1	18.5-106.8	0-47.3	1088670	80596	7.3 (5.9, 11.8)
ARIC	1986-1990	44-66	45.1-94.3	0-30.1	14174	3869	27.4 (21.8, 28.5)
CARDIA	1985-1986	18-30	18.5-61.9	0.5-32.1	5112	233	31.0 (30.8, 31.2)
CHS	1989-1993	65-90	65-106.7	0-22.5	4503	2218	18.0 (11.5, 21.7)
DHS	2000-2002	18-67	29.8-78.2	0.1-15.4	2947	320	12.4 (12.0, 12.9)
FHS Gen3/OMNI 2/NOS	2002-2005	19-83	22.9-94.4	0-15.8	4447	129	12.2 (10.1, 13.8)
FHS Offspring	1971-1975	18-70	20.4-100.7	0-47.3	4823	1483	43.6 (38.6, 45.3)
FHS OMNI 1	1994-1998	27-78	41.8-94.4	0-24.4	448	64	21.0 (18.6, 22.8)
FHS Original Cohort	1975-1982	56-89	59.4-104.9	0-41.1	2013	1044	22.4 (13.0, 29.9)
JHS	2000-2004	20-93	26.2-102.7	0.1-14.2	2362	138	11.7 (10.9, 12.5)
MESA	2000-2002	44-84	45.2-92.9	0-10.9	6777	525	8.5 (7.8, 8.6)
CESCAS	2010-2012	30.3-79.8	31.4-85.6	0-6.9	6773	134	4.9 (4.5, 5.3)
CRONICAS	2009-2012	34.9-91.7	41-98.3	0-8.8	2343	26	7.2 (6.9, 7.5)
ATTICA	2001-2003	18-89	27-99	4-10	1209	67	10.0 (10.0, 10.0)
BRHS	1998-2000	58-81	60.7-95.4	0-16.3	3256	634	14.9 (12.6, 15.7)
CCHS 1976-1978	1976-1978	20.6-93.2	24.9-104.8	0-42.8	13773	4710	29.2 (16.4, 41.1)
CCHS 1981-1983	1981-1983	20.8-89.4	26.1-101.5	0.3-37.7	1467	426	31.8 (15.2, 36.5)
CCHS 2001-2003 and 2011-2015	2001-2015	20.3-93.7	23.9-98.6	0-17.2	3148	228	15.6 (5.9, 16.5)
DanMONICA I	1982-1985	30.5-71.1	31.8-96.6	0-28.2	3921	947	27.5 (27.0, 27.8)
DanMONICA II	1986-1987	29.8-61.1	31.4-85	0.2-24.4	1455	275	24.1 (23.9, 24.3)
DanMONICA III	1991-1992	29.6-71.2	31.5-90	0.1-19.9	1945	367	19.4 (19.0, 19.7)
DETECT	2003	18-95	19-99.1	0-4.6	6224	453	4.0 (1.0, 4.1)
ESTHER	2000-2003	48.2-75.3	50.3-84.5	0-10.5	8158	513	8.0 (6.2, 8.2)
FINRISK 1982	1982	24.2-63.7	25.9-92.5	0-29	7628	1651	28.8 (28.8, 28.9)
FINRISK 1987	1987	24.1-64.2	29.5-87.8	0-23.9	4771	745	23.8 (23.8, 23.9)
FINRISK 1992	1992	24.1-64.2	30.5-83	0.2-19	4945	428	18.9 (18.8, 18.9)
FINRISK 1997	1997	24.2-74.3	29.1-88	0.1-13.9	6298	458	13.8 (13.8, 13.9)
FINRISK 2002	2002	24.1-74.2	28.4-83	0.1-9	8538	264	8.9 (8.8, 8.9)
Friuli I	1985-1986	24.2-65.3	28.1-78	0.1-14	1845	62	12.8 (12.6, 12.9)
Friuli II	1989	24.4-64.4	26.9-74	0.2-9.8	1796	41	9.6 (9.5, 9.6)
Friuli III and Friuli Studio Emostatico	1994-1996	24.4-65.2	27.2-69	0-4.8	2067	25	4.5 (4.2, 4.6)

Table S12 [continued].

**GHS**	2007-2012	35-74	36-79	0-5	13822	783	5.0 (5.0, 5.0)
**Health 2000**	2000-2001	30.3-100.3	33.9-104.7	0-15.3	5954	784	15.1 (14.9, 15.2)
**HUNT**	1995-1997	19.1-101.1	21.2-105.9	0-23.4	61737	10134	22.3 (21.8, 22.9)
**MATISS I**	1983-1984	19.6-69.9	22.1-91	0-21.5	3538	302	20.7 (20.6, 21.0)
**MATISS II and MATISS III**	1986-1996	18.8-77.7	23.4-89.6	0-18.3	4663	248	17.1 (10.3, 17.7)
**MDC**	1991-1995	45.8-68.1	49.2-91	0-25.2	5955	1431	23.7 (22.9, 24.4)
**Moli-sani**	2005-2010	34.6-98.7	37.3-102.5	0-6.8	22596	385	4.3 (3.5, 5.4)
**MONICA-Brianza I**	1986-1987	25.6-66	28.3-87.8	0.1-22.7	1625	168	22.1 (22.0, 22.2)
**MONICA-Brianza II**	1989-1990	25.9-66	29.5-85	0-19.7	1554	129	19.1 (18.9, 19.5)
**MONICA-Brianza III**	1993-1994	26.2-66.6	29.5-80.9	0-15.3	1639	91	14.6 (14.3, 14.9)
**MONICA-Catalonia I and II**	1986-1992	24.8-67.8	26.9-77.8	0.1-11.6	5313	94	8.4 (7.5, 9.7)
**MONICA-Latina**	1982-1987	24.4-66.9	25.8-86.7	0.3-22.6	1723	125	21.2 (19.8, 21.6)
**MONICA/KORA S3**	1994-1995	24.9-75.2	28.6-89.4	0.1-15.2	3942	273	14.1 (13.6, 14.3)
**MONICA/KORA S4**	1999-2001	24.6-75.3	29.3-84	0.1-10	3475	147	8.8 (8.4, 9.2)
**MPP**	1974-1992	26.5-61.2	28.9-96	0-42.3	33182	10184	34.7 (27.9, 37.2)
**Northern Sweden 1986**	1986	25.2-65.1	28.1-90.6	0.3-26	1422	291	25.9 (25.8, 25.9)
**Northern Sweden 1990**	1990	24.1-64.1	35.7-85.8	0.3-22	1456	231	21.8 (21.8, 21.9)
**Northern Sweden 1994**	1994	24.1-74.2	28.4-91.8	0-18	1664	256	17.9 (17.8, 17.9)
**Northern Sweden 1999**	1999	24.1-79.2	29.8-88.3	0.1-13	1888	170	12.8 (12.7, 12.9)
**Northern Sweden 2004 and 2009**	2004-2009	24.1-75.1	25.7-83	0.1-8	3179	102	7.7 (2.9, 7.9)
**PAMELA**	1990-1993	25.6-75.3	29.8-84.7	0.3-12.1	1935	99	11.3 (11.0, 11.7)
**PREVEND**	1997-1998	28-75	29.7-87.7	0-13.3	8117	720	12.5 (12.2, 12.9)
**PRIME/Belfast**	1991-1994	50-60.3	51.1-78.1	0.1-18.8	2386	397	18.0 (18.0, 18.0)
**PRIME/Lille**	1991-1993	49.1-64.3	51.3-74.4	0-10	2419	173	10.0 (10.0, 10.0)
**PRIME/Strasbourg**	1991-1993	49.5-60.4	52.3-70.4	0.1-10	2408	162	10.0 (10.0, 10.0)
**PRIME/Toulouse**	1991-1993	49.2-61	50.3-71	0.1-10	2498	169	10.0 (10.0, 10.0)
**RS-I**	1997-1999	61.4-101	62.2-106.4	0-17.7	3329	757	15.0 (9.8, 15.9)
**RS-II**	2000-2001	55.2-95.5	57.3-106.2	0-14.9	2378	348	13.7 (12.9, 14.2)
**SHHEC**	1984-1995	25.2-75.7	35-93	0-25.1	14053	2500	23.2 (17.8, 23.8)

Table S12 [continued].							
**Tromso 1986-1987**	1986-1987	19.7-62.2	20.7-86	0-24.4	20055	2022	23.9 (23.0, 24.1)
**Tromso 1994-1995**	1994-1995	24.7-97.2	25-105.3	0-16.3	10271	1242	15.7 (9.5, 15.9)
**UK Biobank**	2006-2010	37.4-73.7	40.4-82	0-10	479196	14682	7.1 (6.4, 7.8)
**ULSAM**	1970-1973	48.6-51.1	49.1-97.3	0-46.8	2308	1261	36.9 (28.9, 42.2)
**Estonia**	2002-2013	18.1-85	22-94.9	0-11.5	2813	1147	5.4 (4.1, 6.4)
**Interepid-Kyrgyzstan**	2012	20-70.5	22.1-74.8	0.2-4.3	1242	53	4.1 (4.1, 4.2)
**Kaunas I**	1983-1985	35.3-65	37.2-94.7	0.1-33.8	1352	356	22.2 (14.1, 27.3)
**Kaunas II**	1986-1987	35-64.9	39.1-93.1	0-30	1616	357	20.9 (14.1, 26.2)
**Kaunas III**	1992-1993	33.2-65	35.5-87.8	0-24.9	1171	180	17.9 (10.2, 23.6)
**Novosibirsk 1**	1985-1986	24.2-65	26.3-65	0-14	2828	119	13.1 (10.1, 13.2)
**Novosibirsk 2**	1988-1989	24.3-65	29-65	0-10.7	3016	70	10.0 (9.7, 10.3)
**Novosibirsk 21**	1983-1985	23.2-63.5	37.6-65	0.1-15.1	1481	113	14.5 (12.8, 14.7)
**Novosibirsk 3**	1994-1995	24.6-65	28.9-65	0-4.6	3000	48	3.9 (3.7, 4.1)
**Warsaw 1**	1983-1985	35.1-64.9	38.5-65	0-11.1	1896	103	10.2 (7.2, 10.7)
**Warsaw 2/3**	1988-1993	34.1-64.8	36-65	0-7	2310	40	1.9 (1.6, 6.5)
**PCS**	2012-2015	37-91	40.1-98	0-7.3	8180	122	5.8 (5.3, 6.1)
**PERSIAN Cohort**	2014-2020	35-70	35-77.4	0-7.5	138044	1637	4.8 (3.2, 5.4)
**TLGS**	1999-2005	20.1-88.9	22.9-97.6	0-17.1	10836	1192	15.9 (12.1, 16.5)
**KWLPS**	2012-2017	18-94.1	20.6-101.5	0.1-9.7	4524	4	7.5 (6.2, 8.6)
**Hisayama**	1988	40.9-96.9	46.4-106.8	0-24	2634	525	24.0 (19.8, 24.0)
**Yamagata**	2010-2015	39-77	40.1-83	0-7	20005	139	4.5 (3.2, 5.6)
**AusDiab**	1999-2000	23.1-90.4	30.1-99.4	0-11.6	8774	273	10.1 (9.6, 10.7)
**Dubbo Study**	1988-1989	59-98	59.4-101.7	0-16.1	2102	781	15.3 (14.7, 15.7)

The follow-up quartiles were estimated using the Kaplan-Meier potential follow-up estimator. For some of the cohorts sampling weights are used when estimating the follow-up quartiles. Individuals with cardiovascular disease at baseline were excluded. Min denotes minimum and Max maximum.

Table S13a. Risk factor hazard ratios for cardiovascular disease.

	Geographic regions							
	Global	North America	Latin America	Western Europe	Eastern Europe and Russia	North Africa and Middle East	Asia	Australia
**Women**								
**N**	564698	26348	5167	417682	8934	86929	13489	6149
**No. of events**	25714	4536	59	18393	954	1020	290	462
**Body-mass index per 5kg/m²**	1.07 (1.05, 1.09)	1.07 (1.03, 1.11)	1.26 (0.97, 1.63)	1.09 (1.06, 1.11)	1.03 (0.90, 1.17)	0.94 (0.79, 1.11)	1.20 (0.90, 1.61)	0.97 (0.83, 1.13)
**Systolic blood pressure per 20mmHg**	1.28 (1.24, 1.32)	1.47 (1.29, 1.67)	1.31 (0.98, 1.75)	1.25 (1.22, 1.29)	1.22 (1.05, 1.42)	1.28 (1.17, 1.39)	1.48 (0.96, 2.27)	1.19 (1.04, 1.36)
**Non-HDL cholesterol per 38.67 mg/dL**	1.15 (1.13, 1.18)	1.20 (1.13, 1.28)	1.13 (0.86, 1.48)	1.14 (1.11, 1.17)	1.09 (0.97, 1.23)	1.14 (0.98, 1.33)	0.90 (0.75, 1.09)	1.36 (0.93, 2.00)
**Current smoking**	2.02 (1.88, 2.16)	1.94 (1.57, 2.41)	1.97 (1.02, 3.79)	2.08 (1.94, 2.23)	3.34 (1.89, 5.91)	1.91 (1.32, 2.76)	2.37 (0.51, 11.09)	1.44 (1.07, 1.94)
**Diabetes**	2.12 (1.96, 2.29)	2.05 (1.73, 2.42)	2.69 (0.76, 9.57)	2.22 (1.98, 2.49)	2.30 (1.51, 3.52)	2.24 (1.96, 2.57)	1.36 (0.86, 2.15)	2.33 (1.58, 3.43)
**Men**								
**N**	467951	20627	3899	351580	8969	69217	9050	4609
**No. of events**	37012	5031	74	28351	1177	1566	317	496
**Body-mass index per 5kg/m²**	1.08 (1.05, 1.10)	1.09 (1.04, 1.16)	1.21 (0.82, 1.80)	1.09 (1.06, 1.12)	1.14 (1.01, 1.29)	0.96 (0.88, 1.05)	0.79 (0.58, 1.10)	1.09 (0.92, 1.29)
**Systolic blood pressure per 20mmHg**	1.27 (1.24, 1.29)	1.28 (1.21, 1.35)	1.39 (0.76, 2.56)	1.27 (1.24, 1.29)	1.18 (1.08, 1.29)	1.32 (1.23, 1.41)	1.45 (1.23, 1.72)	1.17 (1.01, 1.35)
**Non-HDL cholesterol per 38.67 mg/dL**	1.23 (1.20, 1.26)	1.27 (1.15, 1.41)	1.24 (0.92, 1.66)	1.23 (1.20, 1.26)	1.10 (0.99, 1.22)	1.28 (1.18, 1.39)	1.23 (1.09, 1.40)	1.19 (1.04, 1.36)
**Current smoking**	1.68 (1.62, 1.76)	1.65 (1.38, 1.98)	0.93 (0.10, 8.75)	1.72 (1.65, 1.79)	1.73 (1.48, 2.03)	1.67 (1.50, 1.87)	1.60 (1.07, 2.40)	1.42 (1.12, 1.81)
**Diabetes**	1.93 (1.82, 2.05)	2.01 (1.73, 2.33)	2.90 (0.72, 11.68)	2.00 (1.85, 2.16)	1.46 (0.87, 2.45)	1.83 (1.56, 2.14)	1.77 (1.19, 2.63)	1.61 (0.98, 2.64)

Hazard ratios with 95% confidence intervals are presented. Individuals with cardiovascular disease at baseline were excluded. Age was used as the time scale. All five risk factors considered were included in the models together with use of antihypertensive medications. A one-year landmark analysis was performed. Confidence interval widths have not been adjusted for multiple comparisons and should not be used in place of hypothesis testing. HDL cholesterol denotes high-density lipoprotein cholesterol. To convert the values for non-HDL cholesterol from milligrams per deciliter (mg/dL) to millimoles per liter (mmol/L), multiply by 0.02586.

Table S13b. Risk factor hazard ratios for all-cause mortality.

	Geographic regions								
	Global	North America	Latin America	Western Europe	Eastern Europe and Russia	North Africa and Middle East	Sub-Saharan Africa	Asia	Australia
**Women**									
**N**	640188	34270	14283	443860	18346	92438	6328	20788	9875
**No. of events**	51601	8579	1020	35393	2868	1529	400	689	1123
**Body-mass index per 5kg/m²**	1.03 (1.01, 1.06)	1.09 (1.05, 1.14)	0.97 (0.90, 1.05)	1.03 (1.00, 1.06)	1.07 (1.00, 1.15)	0.92 (0.82, 1.03)	0.93 (0.76, 1.14)	0.80 (0.57, 1.12)	1.00 (0.87, 1.15)
**Systolic blood pressure per 20mmHg**	1.12 (1.10, 1.14)	1.20 (1.15, 1.26)	1.09 (0.98, 1.21)	1.11 (1.09, 1.14)	1.07 (0.99, 1.16)	1.18 (1.10, 1.26)	1.28 (1.11, 1.48)	1.08 (0.90, 1.31)	1.08 (0.94, 1.25)
**Non-HDL cholesterol per 38.67 mg/dL**	1.00 (0.98, 1.01)	1.00 (0.95, 1.05)	0.83 (0.69, 1.00)	1.01 (1.00, 1.03)	0.97 (0.90, 1.05)	1.03 (0.96, 1.10)	1.01 (0.87, 1.16)	0.87 (0.79, 0.96)	0.97 (0.84, 1.12)
**Current smoking**	2.05 (1.93, 2.18)	2.25 (1.88, 2.68)	1.14 (0.82, 1.60)	2.13 (2.02, 2.25)	2.48 (1.86, 3.30)	2.02 (1.43, 2.84)	1.21 (0.66, 2.22)	1.81 (0.64, 5.16)	2.25 (1.80, 2.82)
**Diabetes**	1.87 (1.76, 1.99)	1.92 (1.57, 2.35)	2.44 (1.93, 3.08)	1.95 (1.80, 2.11)	2.07 (1.63, 2.64)	1.76 (1.47, 2.11)	0.86 (0.32, 2.35)	1.26 (0.88, 1.81)	1.68 (1.13, 2.50)
**Men**									
**N**	537048	26238	11452	372338	16345	83147	3521	15954	8053
**No. of events**	64345	7991	1036	41111	3895	7936	422	732	1222
**Body-mass index per 5kg/m²**	0.99 (0.96, 1.02)	1.06 (1.02, 1.09)	0.84 (0.74, 0.96)	1.03 (1.00, 1.07)	1.00 (0.91, 1.10)	0.92 (0.86, 0.99)	0.64 (0.44, 0.92)	0.72 (0.62, 0.83)	1.01 (0.92, 1.10)
**Systolic blood pressure per 20mmHg**	1.18 (1.15, 1.20)	1.21 (1.13, 1.29)	1.17 (1.01, 1.36)	1.16 (1.13, 1.19)	1.24 (1.17, 1.32)	1.18 (1.11, 1.26)	1.25 (1.06, 1.49)	1.16 (1.06, 1.27)	1.11 (0.98, 1.25)
**Non-HDL cholesterol per 38.67 mg/dL**	0.96 (0.95, 0.98)	0.93 (0.87, 1.00)	0.88 (0.78, 1.00)	0.97 (0.96, 0.99)	0.95 (0.90, 1.00)	0.98 (0.91, 1.06)	0.95 (0.83, 1.09)	0.80 (0.67, 0.97)	0.99 (0.93, 1.06)
**Current smoking**	2.01 (1.92, 2.11)	2.06 (1.81, 2.36)	1.56 (1.20, 2.02)	2.12 (2.01, 2.23)	2.16 (1.94, 2.39)	1.57 (1.38, 1.78)	1.32 (0.85, 2.05)	1.46 (1.18, 1.81)	2.06 (1.67, 2.56)
**Diabetes**	1.80 (1.71, 1.91)	1.66 (1.46, 1.90)	2.50 (1.74, 3.60)	1.78 (1.67, 1.90)	1.57 (1.32, 1.87)	1.85 (1.56, 2.20)	3.02 (0.34, 26.96)	1.76 (1.27, 2.43)	1.91 (1.25, 2.90)

Hazard ratios with 95% confidence intervals are presented. Individuals with cardiovascular disease at baseline were excluded. Age was used as the time scale. All five risk factors considered were included in the models together with use of antihypertensive medications. A one-year landmark analysis was performed. Confidence interval widths have not been adjusted for multiple comparisons and should not be used in place of hypothesis testing. HDL cholesterol denotes high-density lipoprotein cholesterol. To convert the values for non-HDL cholesterol from milligrams per deciliter (mg/dL) to millimoles per liter (mmol/L), multiply by 0.02586.

Table S14. Hazard ratios and subdistribution hazard ratios for five risk factors and cardiovascular disease by sex.

	Geographic region					
	Global		North America		Western Europe	
	Hazard ratio (95% CI)	Subdistribution hazard ratio (95% CI)	Hazard ratio (95% CI)	Subdistribution hazard ratio (95% CI)	Hazard ratio (95% CI)	Subdistribution hazard ratio (95% CI)
**Women**						
**N**	292090		26348		151248	
**N events**	21327		4536		14089	
**Body-mass index per 5kg/m²**	1.07 (1.04, 1.09)	1.06 (1.04, 1.08)	1.07 (1.03, 1.11)	1.05 (1.00, 1.09)	1.08 (1.05, 1.11)	1.07 (1.05, 1.10)
**Systolic blood pressure per 20mmHg**	1.28 (1.25, 1.32)	1.32 (1.28, 1.36)	1.47 (1.29, 1.67)	1.46 (1.28, 1.65)	1.26 (1.22, 1.30)	1.30 (1.26, 1.35)
**Non-HDL cholesterol per 38.67mg/dL**	1.15 (1.12, 1.18)	1.18 (1.15, 1.21)	1.20 (1.13, 1.28)	1.21 (1.14, 1.29)	1.14 (1.11, 1.17)	1.17 (1.14, 1.21)
**Current smoking**	1.99 (1.85, 2.13)	1.72 (1.56, 1.90)	1.94 (1.57, 2.41)	1.62 (1.23, 2.13)	2.01 (1.89, 2.13)	1.75 (1.58, 1.95)
**Diabetes**	2.15 (1.98, 2.33)	2.08 (1.89, 2.28)	2.05 (1.73, 2.42)	1.92 (1.57, 2.34)	2.28 (2.02, 2.57)	2.16 (1.91, 2.45)
**Men**						
**N**	252041		20627		140572	
**N events**	28070		5031		19589	
**Body-mass index per 5kg/m²**	1.07 (1.05, 1.10)	1.06 (1.03, 1.09)	1.09 (1.04, 1.16)	1.04 (0.94, 1.16)	1.08 (1.05, 1.12)	1.07 (1.04, 1.10)
**Systolic blood pressure per 20mmHg**	1.27 (1.24, 1.30)	1.29 (1.26, 1.32)	1.28 (1.21, 1.35)	1.28 (1.20, 1.36)	1.27 (1.24, 1.30)	1.30 (1.28, 1.33)
**Non-HDL cholesterol per 38.67mg/dL**	1.23 (1.20, 1.26)	1.23 (1.18, 1.27)	1.27 (1.15, 1.41)	1.30 (1.18, 1.45)	1.23 (1.20, 1.27)	1.24 (1.21, 1.28)
**Current smoking**	1.68 (1.61, 1.75)	1.40 (1.30, 1.50)	1.65 (1.38, 1.98)	1.31 (0.97, 1.78)	1.72 (1.65, 1.79)	1.44 (1.35, 1.53)
**Diabetes**	1.92 (1.80, 2.05)	1.72 (1.42, 2.07)	2.01 (1.73, 2.33)	1.92 (1.50, 2.47)	2.00 (1.84, 2.19)	1.87 (1.70, 2.05)

Hazard ratios from Cox models for cardiovascular disease and subdistribution hazard ratios from Fine and Gray models for cardiovascular disease with death from non-cardiovascular causes as competing event are presented with 95% confidence intervals. A subset of the available cohort studies was used, since in some cases it was not possible to estimate the models. Individuals with cardiovascular disease at baseline were excluded. The same individuals were used when computing Cox and Fine and Gray models. Age was used as the time scale. All five risk factors considered were included in the models together with use of antihypertensive medications. A one-year landmark analysis was performed. Confidence interval widths have not been adjusted for multiple comparisons and should not be used in place of hypothesis testing. CI denotes confidence interval. HDL cholesterol denotes high-density lipoprotein cholesterol. To convert the values for non-HDL cholesterol from milligrams per deciliter (mg/dL) to millimoles per liter (mmol/L), multiply by 0.02586.

Table S15a. Risk factor hazard ratios for cardiovascular disease using two-years landmark.

	Geographic regions							
	Global	North America	Latin America	Western Europe	Eastern Europe and Russia	North Africa and Middle East	Asia	Australia
**Women**								
**N**	554565	26023	5050	414145	8117	82944	12178	6108
**No. of events**	24113	4346	42	17301	861	861	269	433
**Body-mass index per 5kg/m²**	1.07 (1.05, 1.09)	1.07 (1.03, 1.11)	1.33 (1.00, 1.77)	1.08 (1.06, 1.11)	1.11 (1.01, 1.22)	0.98 (0.89, 1.09)	1.35 (0.79, 2.29)	0.95 (0.84, 1.08)
**Systolic blood pressure per 20mmHg**	1.27 (1.24, 1.31)	1.44 (1.29, 1.61)	1.33 (0.96, 1.83)	1.25 (1.21, 1.29)	1.20 (1.04, 1.38)	1.25 (1.15, 1.35)	1.47 (1.00, 2.17)	1.17 (0.99, 1.39)
**Non-HDL cholesterol per 38.67 mg/dL**	1.15 (1.13, 1.18)	1.19 (1.11, 1.27)	1.10 (0.80, 1.50)	1.14 (1.11, 1.17)	1.05 (0.96, 1.15)	1.17 (1.03, 1.34)	0.82 (0.54, 1.22)	1.38 (0.89, 2.16)
**Current smoking**	2.01 (1.87, 2.15)	1.96 (1.58, 2.44)	2.53 (1.21, 5.31)	2.07 (1.94, 2.21)	2.86 (1.82, 4.49)	1.72 (1.24, 2.39)	2.52 (0.47, 13.50)	1.43 (1.04, 1.97)
**Diabetes**	2.14 (1.97, 2.32)	2.10 (1.74, 2.53)	3.17 (0.59, 16.89)	2.20 (1.94, 2.50)	2.67 (1.78, 3.99)	2.29 (1.98, 2.64)	1.23 (0.64, 2.37)	2.37 (1.61, 3.49)
**Men**								
**N**	457620	20210	3796	346933	8052	66190	7895	4544
**No. of events**	34001	4767	56	26096	1042	1316	283	441
**Body-mass index per 5kg/m²**	1.08 (1.05, 1.11)	1.10 (1.04, 1.17)	1.14 (0.79, 1.64)	1.09 (1.05, 1.12)	1.13 (0.99, 1.29)	0.96 (0.86, 1.07)	0.84 (0.65, 1.09)	1.14 (0.98, 1.32)
**Systolic blood pressure per 20mmHg**	1.27 (1.25, 1.29)	1.28 (1.21, 1.35)	1.39 (0.87, 2.24)	1.27 (1.24, 1.30)	1.16 (1.05, 1.27)	1.34 (1.24, 1.45)	1.40 (1.19, 1.64)	1.13 (1.02, 1.26)
**Non-HDL cholesterol per 38.67 mg/dL**	1.23 (1.21, 1.26)	1.26 (1.14, 1.40)	1.21 (0.90, 1.63)	1.24 (1.21, 1.27)	1.13 (1.04, 1.24)	1.29 (1.19, 1.39)	1.19 (1.05, 1.35)	1.15 (1.04, 1.28)
**Current smoking**	1.68 (1.61, 1.75)	1.65 (1.36, 2.01)	1.19 (0.14, 10.25)	1.70 (1.64, 1.77)	1.75 (1.49, 2.06)	1.67 (1.48, 1.89)	1.65 (1.12, 2.43)	1.49 (1.16, 1.91)
**Diabetes**	1.94 (1.83, 2.07)	2.11 (1.77, 2.51)	3.34 (1.05, 10.60)	2.01 (1.86, 2.18)	1.20 (0.78, 1.85)	1.77 (1.52, 2.07)	1.89 (1.26, 2.84)	1.38 (0.92, 2.06)

Hazard ratios with 95% confidence intervals are presented. Individuals with cardiovascular disease at baseline were excluded. Age was used as the time scale. All five risk factors considered were included in the models together with use of antihypertensive medications. A two-year landmark analysis was performed. Confidence interval widths have not been adjusted for multiple comparisons and should not be used in place of hypothesis testing. HDL cholesterol denotes high-density lipoprotein cholesterol. To convert the values for non-HDL cholesterol from milligrams per deciliter (mg/dL) to millimoles per liter (mmol/L), multiply by 0.02586.

Table S15b. Risk factor hazard ratios for all-cause mortality using two-years landmark.

	Geographic regions								
	**Global**	**North America**	**Latin America**	**Western Europe**	**Eastern Europe and Russia**	**North Africa and Middle East**	**Sub-Saharan Africa**	**Asia**	**Australia**
**Women**									
**N**	621952	34085	12385	441662	17151	88555	6070	12213	9831
**No. of events**	49372	8428	785	34173	2643	1315	366	583	1079
**Body-mass index per 5kg/m²**	1.04 (1.02, 1.07)	1.10 (1.05, 1.15)	1.00 (0.92, 1.09)	1.03 (1.00, 1.07)	1.09 (1.02, 1.17)	0.92 (0.81, 1.04)	0.92 (0.72, 1.19)	0.87 (0.69, 1.08)	1.02 (0.88, 1.18)
**Systolic blood pressure per 20mmHg**	1.12 (1.10, 1.15)	1.19 (1.13, 1.25)	1.12 (0.98, 1.27)	1.11 (1.09, 1.14)	1.07 (0.99, 1.16)	1.19 (1.09, 1.31)	1.30 (1.11, 1.53)	1.10 (0.96, 1.25)	1.09 (0.94, 1.26)
**Non-HDL cholesterol per 38.67 mg/dL**	1.00 (0.99, 1.02)	1.00 (0.95, 1.06)	0.90 (0.80, 1.02)	1.01 (1.00, 1.03)	0.97 (0.89, 1.06)	1.03 (0.96, 1.11)	1.01 (0.87, 1.16)	0.82 (0.71, 0.95)	0.98 (0.83, 1.15)
**Current smoking**	1.64 (1.08, 2.50)	2.22 (1.86, 2.65)	0.10 (0.00, 12.83)	2.14 (2.03, 2.26)	2.39 (1.81, 3.16)	1.89 (1.28, 2.77)	1.19 (0.66, 2.15)	2.89 (0.94, 8.90)	2.28 (1.80, 2.90)
**Diabetes**	1.88 (1.77, 2.00)	1.94 (1.58, 2.38)	2.23 (1.64, 3.04)	1.94 (1.80, 2.09)	2.08 (1.63, 2.67)	1.75 (1.44, 2.12)	0.77 (0.22, 2.68)	1.55 (1.02, 2.35)	1.69 (1.06, 2.68)
**Men**									
**N**	526555	26030	10196	369813	15821	80251	3310	13164	7970
**No. of events**	61182	7819	820	39234	3581	7620	373	596	1139
**Body-mass index per 5kg/m²**	1.00 (0.98, 1.03)	1.06 (1.03, 1.10)	0.84 (0.73, 0.97)	1.04 (1.01, 1.07)	1.04 (0.94, 1.14)	0.92 (0.85, 0.99)	0.67 (0.50, 0.88)	0.62 (0.48, 0.80)	1.05 (0.96, 1.14)
**Systolic blood pressure per 20mmHg**	1.19 (1.16, 1.21)	1.22 (1.14, 1.32)	1.18 (0.99, 1.41)	1.16 (1.13, 1.19)	1.25 (1.17, 1.33)	1.20 (1.13, 1.28)	1.27 (1.11, 1.46)	1.14 (1.02, 1.28)	1.14 (0.98, 1.34)
**Non-HDL cholesterol per 38.67 mg/dL**	0.97 (0.95, 0.98)	0.93 (0.86, 1.00)	0.87 (0.77, 0.98)	0.98 (0.96, 1.00)	0.95 (0.90, 0.99)	0.99 (0.91, 1.06)	0.95 (0.83, 1.09)	0.85 (0.75, 0.97)	0.97 (0.91, 1.05)
**Current smoking**	2.01 (1.92, 2.11)	2.09 (1.82, 2.39)	1.46 (1.06, 2.02)	2.10 (2.00, 2.21)	2.14 (1.92, 2.39)	1.58 (1.42, 1.77)	1.35 (0.83, 2.20)	1.49 (1.17, 1.90)	2.02 (1.55, 2.64)
**Diabetes**	1.81 (1.71, 1.92)	1.69 (1.46, 1.96)	2.43 (1.66, 3.56)	1.79 (1.68, 1.91)	1.58 (1.34, 1.85)	1.91 (1.63, 2.25)	1.75 (0.43, 7.16)	1.80 (1.11, 2.92)	1.89 (1.15, 3.11)

Hazard ratios with 95% confidence intervals are presented. Individuals with cardiovascular disease at baseline were excluded. Age was used as the time scale. All five risk factors considered were included in the models together with use of antihypertensive medications. A two-year landmark analysis was performed. Confidence interval widths have not been adjusted for multiple comparisons and should not be used in place of hypothesis testing. HDL cholesterol denotes high-density lipoprotein cholesterol. To convert the values for non-HDL cholesterol from milligrams per deciliter (mg/dL) to millimoles per liter (mmol/L), multiply by 0.02586.

Table S16a. Risk factor hazard ratios for cardiovascular disease using only studies with examination year greater or equal than 2000.

	Geographic regions						
	**Global**	**North America**	**Latin America**	**Western Europe**	**Eastern Europe and Russia**	**North Africa and Middle East**	**Asia**
**Women**							
**N**	415128	9062	5167	306090	1974	80853	11982
**No. of events**	7166	393	59	5617	525	534	38
**Body-mass index per 5kg/m²**	1.04 (1.00, 1.09)	1.05 (0.93, 1.18)	1.26 (0.97, 1.63)	1.05 (1.00, 1.10)	1.00 (0.72, 1.37)	0.85 (0.63, 1.15)	1.36 (0.81, 2.29)
**Systolic blood pressure per 20mmHg**	1.25 (1.15, 1.37)	1.70 (1.30, 2.22)	1.31 (0.98, 1.75)	1.15 (1.06, 1.24)	1.19 (0.86, 1.65)	1.30 (1.16, 1.45)	1.86 (1.24, 2.78)
**Non-HDL cholesterol per 38.67 mg/dL**	1.11 (1.08, 1.14)	1.14 (1.01, 1.30)	1.13 (0.86, 1.48)	1.10 (1.07, 1.14)	1.16 (0.69, 1.93)	1.06 (0.90, 1.26)	0.84 (0.51, 1.37)
**Current smoking**	2.22 (1.96, 2.51)	2.20 (1.63, 2.99)	1.97 (1.02, 3.79)	2.03 (1.69, 2.43)	4.45 (0.69, 28.48)	2.10 (1.18, 3.71)	5.35 (1.83, 15.65)
**Diabetes**	1.95 (1.72, 2.21)	1.89 (1.33, 2.69)	2.69 (0.76, 9.57)	1.84 (1.53, 2.22)	1.11 (0.20, 6.25)	2.28 (1.86, 2.80)	1.14 (0.28, 4.56)
**Men**							
**N**	330061	7311	3899	244538	1864	64487	7962
**No. of events**	12765	613	74	10639	498	877	64
**Body-mass index per 5kg/m²**	1.05 (1.01, 1.10)	1.05 (0.90, 1.23)	1.21 (0.82, 1.80)	1.13 (1.09, 1.18)	0.90 (0.69, 1.16)	0.86 (0.69, 1.07)	0.69 (0.43, 1.11)
**Systolic blood pressure per 20mmHg**	1.26 (1.20, 1.33)	1.41 (1.28, 1.56)	1.39 (0.76, 2.56)	1.22 (1.15, 1.28)	1.03 (0.84, 1.26)	1.25 (1.03, 1.52)	1.56 (1.16, 2.10)
**Non-HDL cholesterol per 38.67 mg/dL**	1.19 (1.13, 1.25)	1.24 (1.03, 1.49)	1.24 (0.92, 1.66)	1.18 (1.12, 1.26)	0.88 (0.64, 1.20)	1.28 (1.13, 1.46)	1.26 (0.93, 1.72)
**Current smoking**	1.87 (1.76, 1.98)	1.89 (1.40, 2.54)	0.93 (0.10, 8.75)	1.92 (1.72, 2.15)	1.23 (0.38, 4.00)	1.64 (1.41, 1.91)	1.94 (1.12, 3.36)
**Diabetes**	1.82 (1.62, 2.04)	1.89 (1.24, 2.89)	2.90 (0.72, 11.68)	1.74 (1.45, 2.09)	1.08 (0.38, 3.09)	2.06 (1.65, 2.57)	2.09 (1.11, 3.93)

Hazard ratios with 95% confidence intervals are presented. Individuals with cardiovascular disease at baseline were excluded. Age was used as the time scale. All five risk factors considered were included in the models together with use of antihypertensive medications. A one-year landmark analysis was performed. Confidence interval widths have not been adjusted for multiple comparisons and should not be used in place of hypothesis testing. HDL cholesterol denotes high-density lipoprotein cholesterol. To convert the values for non-HDL cholesterol from milligrams per deciliter (mg/dL) to millimoles per liter (mmol/L), multiply by 0.02586.

Table S16b. Risk factor hazard ratios for all-cause mortality using only studies with examination year greater or equal than 2000.

	Geographic regions							
	Global	North America	Latin America	Western Europe	Eastern Europe and Russia	North Africa and Middle East	Sub-Saharan Africa	Asia
**Women**								
**N**	461303	16851	13164	308074	11254	86359	6328	19273
**No. of events**	13477	963	873	7891	1983	1233	400	134
**Body-mass index per 5kg/m²**	1.00 (0.96, 1.04)	1.11 (1.03, 1.21)	0.98 (0.89, 1.06)	1.01 (0.96, 1.06)	1.02 (0.93, 1.12)	0.90 (0.78, 1.03)	0.93 (0.76, 1.14)	0.66 (0.41, 1.08)
**Systolic blood pressure per 20mmHg**	1.09 (1.05, 1.14)	1.21 (1.06, 1.37)	1.06 (0.93, 1.21)	1.04 (1.00, 1.09)	1.05 (0.97, 1.13)	1.19 (1.08, 1.31)	1.28 (1.11, 1.48)	1.12 (0.78, 1.61)
**Non-HDL cholesterol per 38.67 mg/dL**	0.98 (0.95, 1.01)	0.90 (0.82, 1.00)	0.80 (0.63, 1.02)	1.00 (0.96, 1.05)	1.01 (0.93, 1.10)	1.03 (0.93, 1.13)	1.01 (0.87, 1.16)	0.87 (0.65, 1.17)
**Current smoking**	2.07 (1.82, 2.36)	2.73 (2.04, 3.64)	1.43 (1.03, 1.98)	2.22 (1.90, 2.59)	2.60 (2.27, 2.98)	2.18 (1.44, 3.28)	1.21 (0.66, 2.22)	1.93 (0.28, 13.23)
**Diabetes**	1.73 (1.62, 1.85)	1.63 (1.21, 2.20)	2.27 (1.82, 2.85)	1.69 (1.54, 1.84)	1.77 (1.36, 2.31)	1.61 (1.20, 2.14)	0.86 (0.32, 2.35)	1.16 (0.49, 2.72)
**Men**								
**N**	367782	12711	10670	247502	9115	69406	3521	14857
**No. of events**	17152	1036	904	10308	2402	1873	422	207
**Body-mass index per 5kg/m²**	0.97 (0.91, 1.03)	1.03 (0.94, 1.12)	0.86 (0.75, 1.00)	1.07 (1.01, 1.14)	1.01 (0.88, 1.16)	0.89 (0.79, 0.99)	0.64 (0.44, 0.92)	0.93 (0.46, 1.88)
**Systolic blood pressure per 20mmHg**	1.11 (1.08, 1.15)	1.19 (1.03, 1.37)	1.09 (0.94, 1.27)	1.06 (1.02, 1.11)	1.13 (1.05, 1.21)	1.15 (1.06, 1.24)	1.25 (1.06, 1.49)	1.13 (0.95, 1.35)
**Non-HDL cholesterol per 38.67 mg/dL**	0.93 (0.90, 0.95)	0.85 (0.74, 0.97)	0.84 (0.73, 0.97)	0.95 (0.92, 0.97)	0.99 (0.93, 1.06)	0.94 (0.87, 1.00)	0.95 (0.83, 1.09)	0.68 (0.51, 0.90)
**Current smoking**	1.97 (1.75, 2.20)	2.23 (1.81, 2.74)	1.49 (1.09, 2.04)	2.46 (2.25, 2.70)	2.42 (2.03, 2.89)	1.15 (0.85, 1.55)	1.32 (0.85, 2.05)	1.47 (0.92, 2.34)
**Diabetes**	1.66 (1.55, 1.77)	1.50 (1.12, 2.01)	2.18 (1.54, 3.09)	1.61 (1.48, 1.74)	1.47 (1.30, 1.67)	1.76 (1.49, 2.08)	3.02 (0.34, 26.96)	1.66 (0.76, 3.63)

Hazard ratios with 95% confidence intervals are presented. Individuals with cardiovascular disease at baseline were excluded. Age was used as the time scale. All five risk factors considered were included in the models together with use of antihypertensive medications. A one-year landmark analysis was performed. Confidence interval widths have not been adjusted for multiple comparisons and should not be used in place of hypothesis testing. HDL cholesterol denotes high-density lipoprotein cholesterol. To convert the values for non-HDL cholesterol from milligrams per deciliter (mg/dL) to millimoles per liter (mmol/L), multiply by 0.02586.

Table S17a. Risk factor hazard ratios for cardiovascular disease using only individuals with information on lipid-lowering medications use.

	Geographic regions							
	Global	North America	Latin America	Western Europe	Eastern Europe and Russia	North Africa and Middle East	Asia	Australia
**Women**								
**N**	483099	23565	5167	343796	5511	86929	11982	6149
**No. of events**	15842	4443	59	9199	621	1020	38	462
**Body-mass index per 5kg/m²**	1.05 (1.03, 1.08)	1.05 (1.01, 1.10)	1.29 (0.98, 1.69)	1.07 (1.04, 1.10)	1.03 (0.80, 1.33)	0.93 (0.78, 1.12)	1.39 (0.82, 2.33)	0.96 (0.84, 1.10)
**Systolic blood pressure per 20mmHg**	1.27 (1.23, 1.31)	1.40 (1.25, 1.55)	1.37 (0.99, 1.91)	1.23 (1.18, 1.28)	1.25 (0.95, 1.64)	1.28 (1.18, 1.38)	1.84 (1.23, 2.76)	1.19 (1.04, 1.36)
**Non-HDL cholesterol per 38.67 mg/dL**	1.15 (1.12, 1.18)	1.17 (1.10, 1.25)	1.15 (0.88, 1.50)	1.12 (1.09, 1.15)	1.10 (0.92, 1.33)	1.12 (0.96, 1.32)	0.82 (0.49, 1.35)	1.40 (0.90, 2.17)
**Current smoking**	1.97 (1.80, 2.16)	1.90 (1.51, 2.39)	1.67 (0.74, 3.73)	2.04 (1.87, 2.24)	4.27 (1.48, 12.28)	1.89 (1.30, 2.76)	5.13 (1.75, 15.07)	1.42 (1.05, 1.92)
**Diabetes**	1.98 (1.82, 2.16)	2.06 (1.67, 2.54)	2.55 (0.73, 8.92)	1.97 (1.75, 2.21)	1.89 (0.89, 3.99)	2.28 (1.97, 2.63)	1.19 (0.30, 4.76)	2.18 (1.56, 3.04)
**Men**								
**N**	404095	18301	3899	294764	5343	69217	7962	4609
**No. of events**	26117	4891	74	18346	680	1566	64	496
**Body-mass index per 5kg/m²**	1.07 (1.04, 1.10)	1.09 (1.02, 1.16)	1.22 (0.83, 1.78)	1.09 (1.05, 1.12)	1.16 (0.91, 1.49)	0.96 (0.88, 1.05)	0.70 (0.43, 1.13)	1.07 (0.89, 1.29)
**Systolic blood pressure per 20mmHg**	1.27 (1.24, 1.30)	1.28 (1.21, 1.36)	1.39 (0.77, 2.53)	1.27 (1.24, 1.31)	1.16 (1.00, 1.36)	1.32 (1.24, 1.41)	1.55 (1.15, 2.09)	1.17 (1.01, 1.34)
**Non-HDL cholesterol per 38.67 mg/dL**	1.21 (1.18, 1.24)	1.23 (1.14, 1.32)	1.23 (0.92, 1.65)	1.21 (1.17, 1.25)	1.04 (0.91, 1.18)	1.28 (1.18, 1.38)	1.26 (0.93, 1.72)	1.21 (1.02, 1.43)
**Current smoking**	1.70 (1.61, 1.80)	1.61 (1.33, 1.95)	0.94 (0.10, 8.86)	1.75 (1.65, 1.86)	1.82 (1.18, 2.80)	1.67 (1.50, 1.87)	1.92 (1.11, 3.34)	1.41 (1.11, 1.81)
**Diabetes**	1.82 (1.71, 1.93)	1.92 (1.68, 2.21)	2.86 (0.68, 12.03)	1.83 (1.68, 2.00)	1.96 (1.17, 3.26)	1.82 (1.56, 2.12)	2.16 (1.15, 4.08)	1.53 (1.01, 2.31)

Hazard ratios with 95% confidence intervals are presented. Individuals with cardiovascular disease at baseline were excluded. Age was used as the time scale. All five risk factors considered were included in the models together with use of antihypertensive and lipid-lowering medications. A one-year landmark analysis was performed. Confidence interval widths have not been adjusted for multiple comparisons and should not be used in place of hypothesis testing. HDL cholesterol denotes high-density lipoprotein cholesterol. To convert the values for non-HDL cholesterol from milligrams per deciliter (mg/dL) to millimoles per liter (mmol/L), multiply by 0.02586.

Table S17b. Risk factor hazard ratios for all-cause mortality using only individuals with information on lipid-lowering medications use.

	Geographic regions								
	Global	North America	Latin America	Western Europe	Eastern Europe and Russia	North Africa and Middle East	Sub-Saharan Africa	Asia	Australia
**Women**									
**N**	545720	31487	9981	365246	15408	92438	3194	19273	8693
**No. of events**	32931	8406	515	18972	2194	1529	151	134	1030
**Body-mass index per 5kg/m²**	1.03 (1.00, 1.06)	1.09 (1.04, 1.15)	0.96 (0.86, 1.07)	1.03 (0.99, 1.07)	1.10 (0.99, 1.21)	0.92 (0.82, 1.03)	0.86 (0.68, 1.08)	0.67 (0.41, 1.11)	1.02 (0.85, 1.23)
**Systolic blood pressure per 20mmHg**	1.11 (1.08, 1.14)	1.19 (1.13, 1.25)	1.06 (0.93, 1.22)	1.10 (1.06, 1.13)	1.08 (0.98, 1.18)	1.18 (1.10, 1.26)	1.37 (1.18, 1.59)	1.11 (0.77, 1.62)	1.07 (1.00, 1.13)
**Non-HDL cholesterol per 38.67 mg/dL**	0.99 (0.98, 1.01)	0.98 (0.93, 1.03)	0.85 (0.73, 0.99)	1.00 (0.98, 1.02)	1.00 (0.92, 1.08)	1.02 (0.95, 1.09)	1.07 (0.94, 1.21)	0.86 (0.63, 1.17)	0.94 (0.86, 1.03)
**Current smoking**	2.14 (1.98, 2.31)	2.28 (1.88, 2.77)	1.28 (0.83, 2.00)	2.22 (2.06, 2.40)	2.54 (1.72, 3.74)	2.00 (1.42, 2.81)	0.92 (0.34, 2.50)	1.89 (0.29, 12.52)	2.31 (1.76, 3.03)
**Diabetes**	1.83 (1.73, 1.93)	1.79 (1.52, 2.11)	2.48 (1.96, 3.12)	1.82 (1.69, 1.97)	1.91 (1.45, 2.51)	1.79 (1.48, 2.17)	1.25 (0.31, 5.07)	1.21 (0.50, 2.89)	1.74 (1.25, 2.42)
**Men**									
**N**	450957	23912	7337	309198	13261	74147	1303	14857	6942
**No. of events**	40969	7729	515	26202	2836	2302	132	207	1046
**Body-mass index per 5kg/m²**	1.00 (0.97, 1.03)	1.06 (1.02, 1.10)	0.86 (0.72, 1.02)	1.02 (0.98, 1.07)	1.06 (0.95, 1.18)	0.89 (0.82, 0.98)	0.54 (0.38, 0.77)	0.93 (0.47, 1.85)	1.07 (0.96, 1.20)
**Systolic blood pressure per 20mmHg**	1.15 (1.12, 1.18)	1.20 (1.12, 1.29)	1.18 (0.97, 1.43)	1.13 (1.10, 1.16)	1.17 (1.09, 1.25)	1.15 (1.08, 1.23)	1.35 (1.15, 1.59)	1.13 (0.94, 1.35)	1.09 (0.93, 1.29)
**Non-HDL cholesterol per 38.67 mg/dL**	0.96 (0.94, 0.97)	0.92 (0.85, 0.99)	0.92 (0.79, 1.08)	0.96 (0.94, 0.98)	0.97 (0.91, 1.02)	0.95 (0.89, 1.01)	0.99 (0.84, 1.17)	0.67 (0.50, 0.90)	0.96 (0.85, 1.08)
**Current smoking**	2.06 (1.95, 2.18)	2.05 (1.77, 2.37)	1.70 (1.27, 2.29)	2.16 (2.03, 2.30)	2.28 (2.01, 2.58)	1.37 (1.01, 1.85)	1.07 (0.44, 2.63)	1.46 (0.90, 2.37)	1.96 (1.34, 2.86)
**Diabetes**	1.80 (1.68, 1.92)	1.63 (1.44, 1.85)	2.69 (1.58, 4.58)	1.73 (1.64, 1.83)	1.62 (1.40, 1.88)	1.87 (1.50, 2.34)	9.67 (2.53, 36.86)	1.60 (0.63, 4.07)	1.65 (1.08, 2.51)

Hazard ratios with 95% confidence intervals are presented. Individuals with cardiovascular disease at baseline were excluded. Age was used as the time scale. All five risk factors considered were included in the models together with use of antihypertensive and lipid-lowering medications. A one-year landmark analysis was performed. Confidence interval widths have not been adjusted for multiple comparisons and should not be used in place of hypothesis testing. HDL cholesterol denotes high-density lipoprotein cholesterol. To convert the values for non-HDL cholesterol from milligrams per deciliter (mg/dL) to millimoles per liter (mmol/L), multiply by 0.02586.

Table S18. Risk factor hazard ratios for cardiovascular disease. Comparison with a secondary cardiovascular disease end point.

	Geographic regions							
	**Global**	**North America**	**Latin America**	**Western Europe**	**Eastern Europe and Russia**	**North Africa and Middle East**	**Asia**	**Australia**
**CVD** defined as first fatal or non-fatal myocardial infarction, unstable angina, coronary revascularization, stroke, and cardiovascular or unclassifiable death								
**Women**								
**N**	489092	13998	3961	379928	8234	76496	1507	4968
**No. of events**	18405	2638	48	13978	926	491	252	72
**Body-mass index per 5kg/m²**	1.07 (1.06, 1.09)	1.09 (1.01, 1.18)	1.21 (0.93, 1.56)	1.08 (1.06, 1.11)	1.03 (0.89, 1.19)	0.97 (0.87, 1.07)	1.08 (0.88, 1.32)	1.03 (0.80, 1.31)
**Systolic blood pressure per 20mmHg**	1.27 (1.24, 1.31)	1.40 (1.20, 1.64)	1.24 (0.91, 1.69)	1.27 (1.24, 1.31)	1.26 (1.07, 1.49)	1.29 (1.15, 1.45)	1.21 (1.07, 1.37)	1.13 (0.85, 1.50)
**Non-HDL cholesterol per 38.67 mg/dL**	1.14 (1.12, 1.17)	1.18 (1.10, 1.28)	1.08 (0.81, 1.44)	1.15 (1.12, 1.17)	1.06 (0.96, 1.17)	1.13 (1.03, 1.24)	0.97 (0.85, 1.10)	1.68 (1.33, 2.12)
**Current smoking**	2.07 (1.90, 2.26)	1.62 (1.34, 1.96)	2.01 (1.01, 3.99)	2.20 (2.02, 2.39)	2.93 (1.66, 5.18)	2.63 (1.65, 4.17)	1.14 (0.69, 1.89)	1.42 (0.66, 3.06)
**Diabetes**	2.22 (2.01, 2.45)	1.88 (1.42, 2.47)	1.60 (0.81, 3.14)	2.38 (2.10, 2.70)	2.72 (1.84, 4.01)	2.21 (1.82, 2.68)	1.58 (1.11, 2.24)	2.61 (1.22, 5.61)
**Men**								
**N**	411839	10900	2767	324180	8450	60684	1088	3770
**No. of events**	28789	2612	59	23735	1159	806	253	165
**Body-mass index per 5kg/m²**	1.07 (1.04, 1.11)	1.11 (1.02, 1.21)	1.06 (0.78, 1.42)	1.08 (1.04, 1.11)	1.15 (1.00, 1.32)	0.94 (0.86, 1.03)	0.90 (0.71, 1.13)	1.02 (0.83, 1.26)
**Systolic blood pressure per 20mmHg**	1.27 (1.24, 1.30)	1.26 (1.18, 1.34)	1.06 (0.79, 1.43)	1.28 (1.24, 1.31)	1.20 (1.09, 1.31)	1.35 (1.23, 1.49)	1.37 (1.21, 1.56)	1.25 (1.02, 1.53)
**Non-HDL cholesterol per 38.67 mg/dL**	1.25 (1.22, 1.27)	1.26 (1.16, 1.36)	1.13 (0.87, 1.47)	1.26 (1.24, 1.29)	1.12 (1.02, 1.23)	1.22 (1.13, 1.32)	1.21 (1.07, 1.38)	1.26 (1.08, 1.48)
**Current smoking**	1.69 (1.62, 1.77)	1.56 (1.23, 1.99)	2.54 (1.45, 4.44)	1.73 (1.66, 1.80)	1.78 (1.52, 2.08)	1.68 (1.45, 1.95)	1.36 (1.05, 1.76)	1.40 (0.90, 2.17)
**Diabetes**	2.04 (1.90, 2.18)	1.98 (1.59, 2.47)	1.56 (0.79, 3.09)	2.16 (1.98, 2.35)	1.44 (0.84, 2.46)	1.92 (1.63, 2.26)	1.54 (1.13, 2.09)	2.03 (1.24, 3.35)

Table S18 [continued].								
	**Geographic regions**							
	**Global**	**North America**	**Latin America**	**Western Europe**	**Eastern Europe and Russia**	**North Africa and Middle East**	**Asia**	**Australia**
**CVD** defined as only myocardial infarction, coronary death and stroke								
**Women**								
**N**	489092	13998	3961	379928	8234	76496	1507	4968
**No. of events**	15293	1784	22	11974	903	328	241	41
**Body-mass index per 5kg/m²**	1.06 (1.03, 1.08)	1.04 (0.94, 1.14)	1.27 (0.85, 1.90)	1.08 (1.06, 1.10)	1.09 (0.98, 1.21)	0.85 (0.75, 0.97)	1.06 (0.86, 1.30)	1.08 (0.78, 1.50)
**Systolic blood pressure per 20mmHg**	1.31 (1.27, 1.36)	1.51 (1.23, 1.85)	1.31 (0.82, 2.09)	1.30 (1.26, 1.34)	1.24 (1.05, 1.47)	1.43 (1.23, 1.65)	1.26 (1.11, 1.43)	1.50 (1.03, 2.18)
**Non-HDL cholesterol per 38.67 mg/dL**	1.13 (1.11, 1.16)	1.18 (1.09, 1.28)	1.22 (0.79, 1.87)	1.13 (1.11, 1.15)	1.07 (0.96, 1.21)	1.09 (0.98, 1.21)	0.93 (0.82, 1.06)	1.80 (1.32, 2.47)
**Current smoking**	2.19 (1.99, 2.42)	1.74 (1.42, 2.14)	2.43 (0.83, 7.11)	2.31 (2.08, 2.57)	2.48 (1.61, 3.82)	2.57 (1.46, 4.50)	1.11 (0.66, 1.87)	2.21 (0.86, 5.67)
**Diabetes**	2.38 (2.14, 2.65)	2.09 (1.35, 3.24)	1.95 (0.71, 5.34)	2.45 (2.16, 2.77)	2.95 (1.93, 4.53)	2.57 (2.02, 3.27)	1.61 (1.13, 2.30)	2.77 (1.03, 7.44)
**Men**								
**N**	411839	10900	2767	324180	8450	60684	1088	3770
**No. of events**	22921	1649	29	19183	1102	635	234	89
**Body-mass index per 5kg/m²**	1.08 (1.04, 1.12)	1.07 (0.99, 1.16)	1.32 (0.88, 1.97)	1.09 (1.05, 1.13)	1.20 (1.03, 1.40)	0.93 (0.84, 1.03)	0.82 (0.64, 1.04)	1.18 (0.89, 1.57)
**Systolic blood pressure per 20mmHg**	1.30 (1.26, 1.33)	1.35 (1.21, 1.51)	1.12 (0.73, 1.70)	1.29 (1.26, 1.34)	1.17 (1.08, 1.28)	1.43 (1.28, 1.59)	1.40 (1.22, 1.59)	1.27 (0.95, 1.68)
**Non-HDL cholesterol per 38.67 mg/dL**	1.23 (1.20, 1.25)	1.25 (1.15, 1.37)	1.26 (0.86, 1.85)	1.23 (1.20, 1.25)	1.11 (1.01, 1.22)	1.26 (1.16, 1.37)	1.21 (1.06, 1.38)	1.29 (1.04, 1.60)
**Current smoking**	1.79 (1.70, 1.89)	1.73 (1.31, 2.28)	3.99 (1.75, 9.07)	1.81 (1.71, 1.93)	1.69 (1.44, 1.98)	1.89 (1.60, 2.23)	1.43 (1.10, 1.87)	1.59 (0.88, 2.87)
**Diabetes**	2.08 (1.93, 2.25)	2.13 (1.58, 2.88)	1.02 (0.33, 3.13)	2.20 (2.00, 2.41)	1.60 (0.88, 2.90)	1.99 (1.66, 2.38)	1.48 (1.07, 2.05)	1.56 (0.75, 3.24)

Hazard ratios with 95% confidence intervals are presented. Individuals with cardiovascular disease at baseline were excluded. Age was used as the time scale. All five risk factors considered were included in the models together with use of antihypertensive medication. A one-year landmark analysis was performed. Confidence interval widths have not been adjusted for multiple comparisons and should not be used in place of hypothesis testing. HDL cholesterol denotes high-density lipoprotein cholesterol. To convert the values for non-HDL cholesterol from milligrams per deciliter (mg/dL) to millimoles per liter (mmol/L), multiply by 0.02586.

Table S19a. Unadjusted risk factor hazard ratios for cardiovascular disease.

	Geographic regions							
	Global	North America	Latin America	Western Europe	Eastern Europe and Russia	North Africa and Middle East	Asia	Australia
**Women**								
**N**	564698	26348	5167	417682	8934	86929	13489	6149
**N events**	25714	4536	59	18393	954	1020	290	462
**Body-mass index per 5kg/m²**	1.19 (1.17, 1.22)	1.24 (1.16, 1.33)	1.32 (1.06, 1.65)	1.19 (1.16, 1.23)	1.18 (1.08, 1.28)	1.08 (0.92, 1.27)	1.31 (0.99, 1.74)	1.11 (1.00, 1.23)
**Systolic blood pressure per 20mmHg**	1.31 (1.26, 1.36)	1.61 (1.36, 1.91)	1.36 (1.03, 1.79)	1.28 (1.23, 1.32)	1.22 (1.07, 1.38)	1.32 (1.23, 1.42)	1.46 (0.98, 2.17)	1.25 (1.15, 1.36)
**Non-HDL cholesterol per 38.67mg/dL**	1.19 (1.15, 1.23)	1.28 (1.16, 1.41)	1.14 (0.89, 1.47)	1.18 (1.14, 1.22)	1.09 (1.01, 1.18)	1.16 (0.96, 1.40)	1.02 (0.91, 1.16)	1.38 (0.97, 1.97)
**Current smoking**	1.88 (1.75, 2.02)	1.78 (1.42, 2.23)	1.69 (0.89, 3.20)	1.92 (1.78, 2.07)	2.91 (1.73, 4.90)	1.68 (1.23, 2.30)	2.10 (0.53, 8.30)	1.43 (1.07, 1.92)
**Diabetes**	2.56 (2.32, 2.82)	2.93 (2.13, 4.02)	3.02 (0.95, 9.63)	2.51 (2.22, 2.85)	2.80 (2.01, 3.90)	2.54 (2.23, 2.90)	1.80 (1.30, 2.50)	2.50 (1.84, 3.38)
**Men**								
**N**	467951	20627	3899	351580	8969	69217	9050	4609
**N events**	37012	5031	74	28351	1177	1566	317	496
**Body-mass index per 5kg/m²**	1.21 (1.18, 1.24)	1.22 (1.14, 1.31)	1.14 (0.84, 1.54)	1.22 (1.18, 1.26)	1.17 (1.04, 1.33)	1.12 (0.98, 1.27)	1.01 (0.83, 1.23)	1.24 (1.09, 1.41)
**Systolic blood pressure per 20mmHg**	1.29 (1.27, 1.32)	1.34 (1.27, 1.42)	1.33 (0.82, 2.16)	1.30 (1.26, 1.33)	1.19 (1.08, 1.31)	1.32 (1.24, 1.40)	1.41 (1.23, 1.62)	1.18 (1.04, 1.35)
**Non-HDL cholesterol per 38.67mg/dL**	1.25 (1.22, 1.28)	1.27 (1.12, 1.44)	1.20 (0.95, 1.51)	1.26 (1.22, 1.30)	1.15 (1.08, 1.22)	1.31 (1.19, 1.43)	1.19 (1.06, 1.34)	1.19 (1.09, 1.29)
**Current smoking**	1.60 (1.53, 1.67)	1.55 (1.29, 1.86)	1.28 (0.17, 9.41)	1.64 (1.56, 1.72)	1.52 (1.32, 1.76)	1.48 (1.33, 1.65)	1.41 (1.04, 1.92)	1.28 (1.01, 1.62)
**Diabetes**	2.07 (1.94, 2.21)	2.43 (1.99, 2.96)	2.48 (0.81, 7.60)	2.07 (1.90, 2.25)	1.55 (0.96, 2.49)	1.98 (1.76, 2.22)	1.85 (1.41, 2.42)	1.73 (1.25, 2.41)

Hazard ratios with 95% confidence intervals are presented. Individuals with cardiovascular disease at baseline were excluded. Age was used as the time scale. The models included a single risk factor each time. A one-year landmark analysis was performed. Confidence interval widths have not been adjusted for multiple comparisons and should not be used in place of hypothesis testing. HDL cholesterol denotes high-density lipoprotein cholesterol. To convert the values for non-HDL cholesterol from milligrams per deciliter (mg/dL) to millimoles per liter (mmol/L), multiply by 0.02586.

Table S19b. Unadjusted risk factor hazard ratios for all-cause mortality.

	Geographic regions								
	Global	North America	Latin America	Western Europe	Eastern Europe and Russia	North Africa and Middle East	Sub-Saharan Africa	Asia	Australia
**Women**									
**N**	640188	34270	14283	443860	18346	92438	6328	20788	9875
**N events**	51601	8579	1020	35393	2868	1529	400	689	1123
**Body-mass index per 5kg/m²**	1.07 (1.04, 1.10)	1.16 (1.09, 1.23)	1.02 (0.94, 1.10)	1.06 (1.03, 1.09)	1.07 (0.96, 1.20)	0.99 (0.89, 1.10)	0.95 (0.83, 1.10)	0.80 (0.60, 1.06)	1.06 (0.95, 1.18)
**Systolic blood pressure per 20mmHg**	1.12 (1.10, 1.15)	1.23 (1.14, 1.33)	1.07 (0.97, 1.19)	1.11 (1.08, 1.14)	1.10 (1.00, 1.22)	1.17 (1.10, 1.25)	1.28 (1.12, 1.46)	1.06 (0.94, 1.19)	1.08 (1.01, 1.15)
**Non-HDL cholesterol per 38.67mg/dL**	1.02 (1.00, 1.04)	1.03 (0.96, 1.12)	0.90 (0.82, 0.98)	1.03 (1.01, 1.06)	1.01 (0.94, 1.08)	1.02 (0.97, 1.08)	1.02 (0.90, 1.15)	0.88 (0.81, 0.95)	1.00 (0.88, 1.13)
**Current smoking**	2.00 (1.89, 2.12)	2.07 (1.75, 2.44)	1.18 (0.80, 1.73)	2.05 (1.93, 2.17)	2.25 (1.69, 2.99)	1.90 (1.38, 2.61)	1.39 (0.90, 2.17)	1.99 (0.79, 5.02)	2.17 (1.79, 2.63)
**Diabetes**	2.02 (1.88, 2.17)	2.35 (1.75, 3.16)	2.19 (1.80, 2.65)	2.03 (1.85, 2.22)	2.19 (1.67, 2.86)	1.85 (1.53, 2.24)	0.82 (0.38, 1.78)	1.28 (0.96, 1.69)	1.78 (1.33, 2.39)
**Men**									
**N**	537048	26238	11452	372338	16345	83147	3521	15954	8053
**N events**	64345	7991	1036	41111	3895	7936	422	732	1222
**Body-mass index per 5kg/m²**	1.01 (0.98, 1.04)	1.07 (1.02, 1.13)	0.87 (0.79, 0.97)	1.04 (1.00, 1.08)	0.99 (0.90, 1.09)	1.00 (0.91, 1.10)	0.67 (0.55, 0.81)	0.70 (0.61, 0.80)	1.02 (0.93, 1.11)
**Systolic blood pressure per 20mmHg**	1.16 (1.13, 1.19)	1.22 (1.14, 1.31)	1.18 (1.01, 1.37)	1.14 (1.11, 1.17)	1.22 (1.17, 1.28)	1.15 (1.06, 1.25)	1.21 (1.01, 1.45)	1.14 (1.04, 1.25)	1.09 (0.97, 1.23)
**Non-HDL cholesterol per 38.67mg/dL**	0.97 (0.95, 0.99)	0.92 (0.84, 1.01)	0.86 (0.77, 0.96)	0.98 (0.96, 1.01)	0.96 (0.91, 1.01)	1.00 (0.91, 1.09)	0.92 (0.80, 1.05)	0.77 (0.63, 0.95)	1.00 (0.95, 1.06)
**Current smoking**	1.94 (1.85, 2.03)	2.01 (1.75, 2.32)	1.42 (1.10, 1.84)	2.03 (1.92, 2.14)	2.08 (1.85, 2.35)	1.42 (1.28, 1.56)	1.56 (1.24, 1.96)	1.62 (1.37, 1.92)	1.98 (1.55, 2.53)
**Diabetes**	1.85 (1.74, 1.96)	1.84 (1.55, 2.20)	2.39 (1.67, 3.40)	1.84 (1.73, 1.97)	1.51 (1.28, 1.76)	1.91 (1.67, 2.19)	2.14 (0.37, 12.47)	1.63 (1.07, 2.47)	1.88 (1.27, 2.79)

Hazard ratios with 95% confidence intervals are presented. Individuals with cardiovascular disease at baseline were excluded. Age was used as the time scale. The models included a single risk factor each time. A one-year landmark analysis was performed. Confidence interval widths have not been adjusted for multiple comparisons and should not be used in place of hypothesis testing. HDL cholesterol denotes high-density lipoprotein cholesterol. To convert the values for non-HDL cholesterol from milligrams per deciliter (mg/dL) to millimoles per liter (mmol/L), multiply by 0.02586.

Table S20a. Population-attributable fractions for 10-year cardiovascular disease by risk factor categories, geographic region and sex.

	Global		North America		Latin America		Western Europe		Eastern Europe and Russia		North Africa and Middle East		Asia		Australia	
	Women	Men	Women	Men	Women	Men	Women	Men	Women	Men	Women	Men	Women	Men	Women	Men
**Body-mass index — kg/m²**	7.6 (5.1, 10.1)	7.6 (5.6, 9.7)	8.4 (5.7, 11.2)	9.8 (7.3, 12.3)	7.1 (4.6, 9.6)	7.2 (5.3, 9.2)	7.5 (4.9, 10.1)	8.5 (6.3, 10.7)	9.3 (6.3, 12.2)	8.0 (5.9, 10.1)	9.0 (6.3, 11.8)	7.6 (5.5, 9.6)	5.8 (3.6, 8.0)	3.9 (1.0, 6.9)	6.1 (3.8, 8.4)	7.3 (5.4, 9.2)
**< 20**	0.6 (0.2, 1.0)	0.4 (-0.3, 1.2)	0.3 (0.1, 0.5)	0.1 (-0.1, 0.2)	0.3 (0.0, 0.6)	0.0 (-0.3, 0.4)	0.3 (0.1, 0.4)	0.1 (0.0, 0.2)	0.3 (0.1, 0.4)	0.2 (-0.1, 0.4)	0.7 (0.3, 1.2)	0.7 (-0.3, 1.7)	2.8 (1.2, 4.4)	2.0 (-0.9, 4.8)	0.3 (0.1, 0.6)	0.0 (-0.1, 0.2)
**25 - < 30**	1.4 (0.2, 2.6)	2.9 (2.0, 3.8)	1.4 (0.2, 2.5)	3.0 (2.1, 3.9)	1.6 (0.3, 2.9)	3.3 (2.3, 4.3)	1.6 (0.2, 2.9)	3.8 (2.6, 4.9)	1.4 (0.2, 2.6)	3.3 (2.2, 4.3)	1.2 (0.2, 2.3)	2.6 (1.8, 3.4)	1.2 (0.2, 2.3)	1.4 (0.9, 1.8)	1.7 (0.3, 3.2)	3.9 (2.7, 5.0)
**≥ 30**	5.6 (4.0, 7.2)	4.3 (3.1, 5.5)	6.8 (4.8, 8.7)	6.8 (4.9, 8.7)	5.3 (3.7, 6.8)	3.9 (2.8, 5.0)	5.7 (4.0, 7.3)	4.6 (3.3, 5.9)	7.6 (5.5, 9.8)	4.5 (3.2, 5.8)	7.1 (5.1, 9.0)	4.2 (3.0, 5.5)	1.8 (1.3, 2.4)	0.6 (0.4, 0.8)	4.0 (2.8, 5.2)	3.4 (2.5, 4.4)
**Systolic blood pressure — mmHg**	29.3 (25.4, 33.2)	21.6 (18.7, 24.5)	28.8 (25.0, 32.7)	19.3 (16.5, 22.2)	29.7 (25.8, 33.7)	23.4 (20.4, 26.4)	25.3 (21.6, 29.1)	20.6 (17.6, 23.7)	37.0 (32.9, 41.1)	28.1 (24.9, 31.3)	33.9 (29.9, 38.0)	26.1 (22.9, 29.2)	28.0 (24.2, 31.8)	19.1 (16.5, 21.7)	33.2 (29.2, 37.1)	27.0 (23.9, 30.2)
**120 - < 140**	9.2 (7.0, 11.4)	7.7 (6.0, 9.3)	9.4 (7.1, 11.6)	7.9 (6.2, 9.6)	9.2 (7.0, 11.3)	7.6 (6.0, 9.3)	10.5 (8.0, 13.0)	9.1 (7.2, 11.1)	7.7 (5.9, 9.5)	7.3 (5.8, 8.9)	8.4 (6.4, 10.4)	7.7 (6.1, 9.4)	8.8 (6.7, 10.9)	6.9 (5.4, 8.4)	8.4 (6.4, 10.4)	6.4 (5.1, 7.8)
**140 - < 160**	10.0 (8.9, 11.1)	8.1 (7.0, 9.1)	10.4 (9.2, 11.6)	8.0 (6.9, 9.1)	9.3 (8.3, 10.3)	8.2 (7.1, 9.2)	10.7 (9.6, 11.9)	8.8 (7.7, 9.9)	11.3 (10.1, 12.4)	9.8 (8.6, 10.9)	11.6 (10.4, 12.8)	8.7 (7.6, 9.8)	8.6 (7.5, 9.6)	6.1 (5.3, 7.0)	11.3 (10.1, 12.5)	11.2 (9.8, 12.5)
**≥ 160**	10.2 (9.1, 11.2)	5.8 (5.3, 6.4)	9.1 (8.1, 10.1)	3.4 (3.0, 3.8)	11.3 (10.1, 12.5)	7.6 (6.9, 8.3)	4.1 (3.7, 4.5)	2.8 (2.5, 3.0)	18.0 (16.2, 19.7)	11.0 (10.0, 12.0)	13.9 (12.5, 15.4)	9.6 (8.7, 10.5)	10.6 (9.4, 11.8)	6.0 (5.4, 6.6)	13.5 (12.2, 14.8)	9.4 (8.6, 10.3)
**Non-HDL cholesterol — mg/dL**	15.4 (10.9, 19.8)	16.6 (12.6, 20.6)	13.5 (9.3, 17.6)	15.1 (11.3, 18.9)	15.7 (11.2, 20.3)	17.3 (13.2, 21.5)	19.1 (14.2, 24.0)	22.5 (18.0, 27.0)	15.5 (11.0, 20.0)	14.8 (11.0, 18.5)	15.6 (11.2, 19.9)	16.1 (12.4, 19.7)	15.4 (10.7, 20.0)	14.2 (10.3, 18.2)	21.7 (16.6, 26.9)	26.1 (21.5, 30.7)
**116.01 – < 154.68**	3.7 (1.7, 5.6)	3.6 (1.7, 5.4)	3.8 (1.8, 5.8)	3.5 (1.6, 5.3)	3.8 (1.9, 5.7)	4.0 (2.0, 6.1)	3.2 (1.5, 4.8)	3.0 (1.4, 4.6)	3.6 (1.6, 5.6)	3.3 (1.4, 5.1)	3.3 (1.5, 5.1)	2.8 (1.3, 4.4)	3.8 (1.7, 5.9)	3.9 (1.8, 6.1)	2.6 (1.3, 4.0)	2.6 (1.3, 3.8)
**154.68 – < 193.35**	5.7 (3.9, 7.5)	7.1 (5.5, 8.6)	4.7 (3.2, 6.2)	6.4 (5.0, 7.9)	6.4 (4.5, 8.4)	7.8 (6.1, 9.5)	6.8 (4.8, 8.9)	8.9 (7.1, 10.8)	5.5 (3.8, 7.3)	5.9 (4.6, 7.3)	4.6 (3.1, 6.1)	5.5 (4.3, 6.8)	5.7 (3.8, 7.5)	6.4 (4.9, 7.8)	7.4 (5.2, 9.6)	9.7 (7.8, 11.7)
**≥ 193.35**	6.0 (4.8, 7.2)	6.0 (5.1, 6.9)	5.0 (4.0, 6.0)	5.2 (4.4, 6.0)	5.5 (4.4, 6.6)	5.5 (4.7, 6.3)	9.1 (7.4, 10.7)	10.5 (9.1, 12.0)	6.4 (5.1, 7.6)	5.6 (4.7, 6.4)	7.7 (6.1, 9.2)	7.7 (6.5, 8.9)	5.9 (4.7, 7.0)	3.9 (3.3, 4.6)	11.7 (9.6, 13.8)	13.8 (12.0, 15.6)
**Current smoking**	6.7 (5.8, 7.6)	10.7 (9.6, 11.7)	7.0 (6.0, 8.0)	7.9 (7.1, 8.7)	6.6 (5.7, 7.5)	9.6 (8.6, 10.5)	8.7 (7.5, 10.0)	8.6 (7.7, 9.4)	3.0 (2.6, 3.5)	16.3 (14.9, 17.8)	4.8 (4.2, 5.5)	13.3 (12.1, 14.6)	5.4 (4.4, 6.4)	20.2 (18.4, 22.0)	5.7 (4.9, 6.5)	5.7 (5.1, 6.3)
**Diabetes**	15.2 (13.3, 17.1)	10.2 (9.2, 11.2)	12.8 (11.2, 14.5)	11.0 (10.0, 12.0)	16.1 (14.1, 18.1)	8.1 (7.3, 8.9)	11.5 (10.0, 13.0)	7.4 (6.7, 8.1)	15.7 (13.5, 17.9)	9.2 (7.8, 10.6)	25.4 (22.5, 28.4)	19.3 (17.0, 21.6)	24.8 (21.6, 28.0)	14.1 (11.7, 16.5)	6.6 (5.7, 7.5)	5.5 (5.0, 6.1)

Geographic regions: Global through Australia.

Table S20a [continued].

**Aggregate PAFs**	57.2 (52.4, 62.1)	52.6 (49.0, 56.1)	55.5 (50.7, 60.3)	50.3 (46.8, 53.8)	57.9 (53.1, 62.8)	51.9 (48.3, 55.6)	55.8 (50.7, 60.9)	53.2 (49.4, 57.1)	60.2 (55.6, 64.9)	57.6 (54.3, 61.0)	64.2 (59.8, 68.6)	60.5 (57.2, 63.9)	59.2 (54.5, 63.8)	55.6 (51.8, 59.4)	57.8 (52.8, 62.7)	56.5 (52.7, 60.3)

Population-attributable fractions (95% confidence intervals) are presented. Weibull models used during the computations included all risk factor simultaneously. Confidence interval widths have not been adjusted for multiple comparisons and should not be used in place of hypothesis testing. Non-HDL cholesterol denotes non-high-density lipoprotein cholesterol. To convert the values for non-HDL cholesterol from milligrams per deciliter (mg/dL) to millimoles per liter (mmol/L), multiply by 0.02586.

Table S20b. Population-attributable fractions for 10-year all-cause mortality by risk factor categories, geographic region and sex.

	**Geographic regions**																	
	**Global**		**North America**		**Latin America**		**Western Europe**		**Eastern Europe and Russia**		**North Africa and Middle East**		**Sub-Saharan Africa**		**Asia**		**Australia**	
	**Women**	**Men**	**Women**	**Men**	**Women**	**Men**	**Women**	**Men**	**Women**	**Men**	**Women**	**Men**	**Women**	**Men**	**Women**	**Men**	**Women**	**Men**
**Body-mass index — kg/m²**	3.4 (1.0, 5.8)	0.7 (-1.3, 2.6)	2.8 (0.0, 5.5)	-2.5 (-5.3, 0.2)	2.3 (0.0, 4.6)	-2.5 (-4.4, -0.5)	1.6 (-1.1, 4.3)	-5.4 (-8.1, -2.6)	2.8 (-0.4, 5.9)	-3.5 (-6.0, -1.1)	5.2 (2.5, 7.8)	2.2 (0.3, 4.1)	8.8 (6.8, 10.9)	11.9 (9.9, 14.0)	11.9 (10.0, 13.9)	13.9 (11.9, 16.0)	1.1 (-1.1, 3.2)	-5.2 (-7.4, -3.0)
**< 20**	3.0 (2.5, 3.5)	4.6 (3.9, 5.2)	1.6 (1.3, 1.9)	1.2 (1.0, 1.5)	2.4 (2.0, 2.8)	2.2 (1.9, 2.5)	1.4 (1.2, 1.7)	0.5 (0.4, 0.6)	1.3 (1.0, 1.5)	1.6 (1.3, 1.8)	3.6 (3.0, 4.2)	5.8 (5.0, 6.7)	7.4 (6.2, 8.6)	14.2 (12.3, 16.1)	13.0 (11.1, 14.9)	15.6 (13.6, 17.5)	2.1 (1.7, 2.4)	0.8 (0.7, 1.0)
**25 - < 30**	-2.5 (-3.5, -1.4)	-4.7 (-5.7, -3.7)	-2.4 (-3.4, -1.4)	-5.2 (-6.3, -4.0)	-2.7 (-3.8, -1.5)	-5.4 (-6.5, -4.2)	-2.8 (-4.1, -1.6)	-6.9 (-8.5, -5.4)	-2.6 (-3.7, -1.5)	-6.1 (-7.4, -4.8)	-2.2 (-3.1, -1.2)	-4.5 (-5.4, -3.5)	-1.5 (-2.1, -0.9)	-2.5 (-3.1, -2.0)	-1.8 (-2.5, -1.1)	-1.7 (-2.1, -1.4)	-3.0 (-4.3, -1.7)	-6.7 (-8.2, -5.2)
**≥ 30**	2.9 (1.3, 4.5)	0.8 (-0.2, 1.9)	3.5 (1.6, 5.5)	1.4 (-0.4, 3.1)	2.6 (1.1, 4.0)	0.7 (-0.2, 1.7)	3.0 (1.3, 4.8)	1.1 (-0.3, 2.4)	4.1 (1.8, 6.4)	1.0 (-0.3, 2.3)	3.7 (1.6, 5.8)	0.8 (-0.2, 1.9)	2.9 (1.3, 4.6)	0.3 (-0.1, 0.7)	0.8 (0.3, 1.2)	0.1 (0.0, 0.2)	2.0 (0.9, 3.1)	0.7 (-0.2, 1.6)
**Systolic blood pressure — mmHg**	11.4 (9.0, 13.8)	10.4 (7.8, 12.9)	11.3 (8.8, 13.7)	8.9 (6.5, 11.4)	11.7 (9.3, 14.1)	11.6 (9.0, 14.3)	8.8 (6.6, 11.0)	9.1 (6.4, 11.8)	16.0 (13.0, 19.0)	15.6 (12.4, 18.8)	13.9 (11.1, 16.7)	13.5 (10.6, 16.5)	14.6 (11.9, 17.3)	13.1 (10.3, 15.8)	10.1 (7.8, 12.3)	8.6 (6.4, 10.8)	14.0 (11.3, 16.7)	15.7 (12.7, 18.8)
**120 – < 140**	2.2 (1.1, 3.3)	1.4 (0.1, 2.6)	2.2 (1.1, 3.3)	1.4 (0.1, 2.7)	2.2 (1.1, 3.2)	1.4 (0.1, 2.6)	2.5 (1.3, 3.8)	1.7 (0.2, 3.3)	2.0 (1.0, 3.0)	1.4 (0.1, 2.7)	2.1 (1.1, 3.1)	1.4 (0.1, 2.7)	1.6 (0.8, 2.4)	1.1 (0.1, 2.1)	2.1 (1.1, 3.1)	1.2 (0.1, 2.3)	2.1 (1.1, 3.1)	1.1 (0.1, 2.1)
**140 – < 160**	3.9 (2.9, 4.8)	4.6 (3.7, 5.6)	4.1 (3.1, 5.1)	4.8 (3.8, 5.8)	3.6 (2.7, 4.5)	4.7 (3.7, 5.6)	4.1 (3.1, 5.1)	5.2 (4.1, 6.3)	4.6 (3.5, 5.7)	5.9 (4.7, 7.2)	4.7 (3.6, 5.8)	5.2 (4.1, 6.2)	4.3 (3.3, 5.4)	5.3 (4.2, 6.4)	3.0 (2.3, 3.8)	3.2 (2.5, 3.9)	4.5 (3.4, 5.6)	6.9 (5.5, 8.2)
**≥ 160**	5.3 (4.5, 6.1)	4.3 (3.8, 4.9)	5.0 (4.2, 5.7)	2.7 (2.3, 3.1)	5.9 (5.1, 6.8)	5.6 (4.8, 6.3)	2.2 (1.8, 2.5)	2.2 (1.9, 2.4)	9.3 (8.0, 10.7)	8.2 (7.2, 9.3)	7.2 (6.1, 8.2)	7.0 (6.0, 7.9)	8.7 (7.4, 9.9)	6.7 (5.7, 7.7)	5.0 (4.2, 5.8)	4.1 (3.6, 4.7)	7.5 (6.4, 8.5)	7.8 (6.8, 8.7)
**Non-HDL cholesterol — mg/dL**	-11.2 (-15.3, -7.1)	-17.3 (-20.3, -14.4)	-10.1 (-13.7, -6.5)	-15.7 (-18.4, -12.9)	-11.5 (-15.7, -7.4)	-18.3 (-21.5, -15.1)	-13.4 (-18.6, -8.2)	-23.6 (-27.9, -19.3)	-11.9 (-16.3, -7.6)	-17.1 (-19.9, -14.2)	-10.7 (-14.7, -6.7)	-15.6 (-18.2, -13.0)	-10.3 (-14.0, -6.6)	-10.9 (-12.5, -9.2)	-11.8 (-16.1, -7.6)	-16.7 (-19.5, -13.8)	-14.1 (-19.7, -8.5)	-24.5 (-29.0, -20.0)

Table S20b [continued].																		
**116.01 – < 154.68**	-5.2 (-7.0, -3.4)	-8.2 (-9.7, -6.7)	-5.2 (-7.1, -3.4)	-7.9 (-9.3, -6.4)	-5.2 (-7.0, -3.4)	-8.7 (-10.4, -7.1)	-4.8 (-6.5, -3.1)	-8.3 (-9.9, -6.7)	-5.5 (-7.4, -3.6)	-8.4 (-9.9, -6.8)	-4.9 (-6.5, -3.2)	-6.8 (-8.1, -5.6)	-5.0 (-6.7, -3.3)	-5.1 (-6.0, -4.2)	-5.7 (-7.7, -3.7)	-9.4 (-11.1, -7.7)	-4.1 (-5.6, -2.6)	-6.7 (-8.0, -5.4)
**154.68 – < 193.35**	-4.4 (-5.9, -2.9)	-6.8 (-7.9, -5.7)	-3.5 (-4.7, -2.3)	-5.9 (-6.8, -4.9)	-4.9 (-6.6, -3.2)	-7.4 (-8.6, -6.2)	-5.8 (-7.8, -3.8)	-10.3 (-12.0, -8.6)	-4.6 (-6.2, -3.0)	-6.2 (-7.2, -5.3)	-3.7 (-5.0, -2.4)	-5.5 (-6.4, -4.7)	-3.8 (-5.1, -2.5)	-4.3 (-5.0, -3.7)	-4.5 (-6.1, -3.0)	-5.8 (-6.8, -4.9)	-6.4 (-8.7, -4.1)	-11.2 (-13.1, -9.2)
**≥ 193.35**	-1.6 (-2.6, -0.7)	-2.3 (-2.8, -1.8)	-1.4 (-2.1, -0.6)	-1.9 (-2.3, -1.5)	-1.4 (-2.3, -0.6)	-2.2 (-2.6, -1.7)	-2.8 (-4.4, -1.2)	-5.0 (-6.2, -3.9)	-1.8 (-2.9, -0.8)	-2.5 (-3.0, -2.0)	-2.1 (-3.4, -0.9)	-3.2 (-3.9, -2.6)	-1.5 (-2.3, -0.6)	-1.4 (-1.7, -1.1)	-1.6 (-2.5, -0.7)	-1.5 (-1.8, -1.2)	-3.6 (-5.7, -1.5)	-6.6 (-8.1, -5.1)
**Current smoking**	6.8 (6.1, 7.6)	14.4 (13.3, 15.4)	7.0 (6.3, 7.8)	10.1 (9.3, 10.9)	6.5 (5.8, 7.2)	12.8 (11.9, 13.8)	8.4 (7.4, 9.4)	10.5 (9.6, 11.4)	3.4 (3.0, 3.8)	22.1 (20.6, 23.6)	5.1 (4.6, 5.6)	16.4 (15.2, 17.6)	6.4 (5.5, 7.2)	14.4 (13.2, 15.6)	7.2 (6.1, 8.3)	28.0 (26.2, 29.7)	5.8 (5.2, 6.4)	7.0 (6.4, 7.5)
**Diabetes**	12.2 (11.1, 13.3)	9.9 (8.9, 10.9)	10.7 (9.7, 11.7)	11.0 (10.0, 12.0)	12.9 (11.7, 14.1)	7.9 (7.1, 8.7)	9.6 (8.7, 10.6)	8.4 (7.6, 9.2)	12.4 (10.9, 13.9)	8.5 (7.2, 9.8)	20.2 (18.2, 22.2)	18.2 (16.0, 20.4)	13.2 (11.3, 15.2)	8.0 (6.8, 9.1)	19.7 (17.1, 22.3)	12.2 (9.7, 14.8)	5.5 (5.0, 6.1)	6.2 (5.6, 6.8)
**Aggregate PAFs**	22.2 (16.8, 27.5)	19.1 (14.6, 23.6)	21.2 (15.9, 26.4)	13.7 (8.7, 18.8)	21.7 (16.3, 27.1)	13.9 (9.0, 18.8)	15.7 (9.3, 22.0)	2.1 (-4.3, 8.6)	22.4 (16.2, 28.5)	27.7 (22.9, 32.6)	31.8 (26.4, 37.1)	34.1 (29.9, 38.3)	30.8 (26.0, 35.5)	33.5 (30.2, 36.7)	34.3 (29.7, 38.9)	43.2 (39.8, 46.6)	13.7 (7.1, 20.3)	2.9 (-3.7, 9.5)

Population-attributable fractions (95% confidence intervals) are presented. Weibull models used during the computations included all risk factor simultaneously. Confidence interval widths have not been adjusted for multiple comparisons and should not be used in place of hypothesis testing. Non-HDL cholesterol denotes non-high-density lipoprotein cholesterol. To convert the values for non-HDL cholesterol from milligrams per deciliter (mg/dL) to millimoles per liter (mmol/L), multiply by 0.02586

Table S21a. Unadjusted population-attributable fractions for 10-year cardiovascular disease by risk factor categories, geographic region and sex.

	Geographic Regions															
	Global		North America		Latin America		Western Europe		Eastern Europe and Russia		North Africa and Middle East		Asia		Australia	
	Women	Men	Women	Men	Women	Men	Women	Men	Women	Men	Women	Men	Women	Men	Women	Men
**Body-mass index — kg/m²**	18.4 (16.0, 20.8)	14.6 (12.6, 16.6)	20.4 (17.8, 23.0)	19.5 (17.2, 21.8)	17.9 (15.5, 20.4)	14.1 (12.2, 16.0)	18.4 (16.0, 20.8)	16.7 (14.7, 18.8)	21.8 (19.1, 24.4)	15.3 (13.3, 17.3)	21.2 (18.6, 23.8)	13.5 (11.4, 15.6)	10.5 (8.4, 12.5)	3.7 (0.7, 6.7)	15.4 (13.3, 17.6)	14.5 (12.7, 16.4)
**< 20**	0.4 (0.0, 0.8)	-0.1 (-0.8, 0.6)	0.2 (0.0, 0.5)	-0.1 (-0.2, 0.1)	0.2 (-0.1, 0.5)	-0.2 (-0.6, 0.1)	0.2 (0.0, 0.4)	0.0 (-0.1, 0.1)	0.2 (0.0, 0.4)	0.0 (-0.2, 0.2)	0.6 (0.1, 1.1)	-0.1 (-1.1, 1.0)	2.2 (0.4, 4.1)	-0.1 (-3.1, 2.9)	0.2 (-0.1, 0.5)	-0.1 (-0.2, 0.0)
**25 - < 30**	5.0 (3.9, 6.1)	5.5 (4.6, 6.4)	4.7 (3.7, 5.7)	5.4 (4.5, 6.2)	5.5 (4.3, 6.7)	6.4 (5.4, 7.5)	5.5 (4.3, 6.7)	7.4 (6.2, 8.6)	5.1 (4.0, 6.2)	6.4 (5.3, 7.5)	4.1 (3.2, 5.0)	4.8 (4.0, 5.6)	4.1 (3.2, 5.1)	2.5 (2.1, 3.0)	5.9 (4.6, 7.3)	7.6 (6.4, 8.8)
**≥ 30**	13.1 (11.3, 14.9)	9.2 (7.9, 10.5)	15.5 (13.4, 17.6)	14.2 (12.3, 16.0)	12.3 (10.6, 14.0)	7.9 (6.8, 9.0)	12.8 (11.1, 14.5)	9.4 (8.1, 10.7)	16.5 (14.4, 18.6)	8.9 (7.6, 10.1)	16.5 (14.4, 18.7)	8.8 (7.5, 10.0)	4.1 (3.5, 4.7)	1.3 (1.1, 1.5)	9.3 (7.9, 10.6)	7.0 (6.0, 8.0)
**Systolic blood pressure — mmHg**	35.5 (31.6, 39.5)	25.1 (22.5, 27.7)	35.4 (31.5, 39.4)	22.7 (20.2, 25.2)	35.9 (31.9, 39.9)	27.0 (24.4, 29.7)	31.3 (27.4, 35.2)	24.0 (21.2, 26.7)	42.4 (38.3, 46.5)	31.5 (28.7, 34.4)	39.7 (35.7, 43.8)	30.0 (27.2, 32.8)	32.9 (29.0, 36.7)	22.1 (19.7, 24.5)	39.8 (35.7, 43.9)	31.2 (28.4, 34.0)
**120 - < 140**	10.9 (9.0, 12.9)	8.6 (7.1, 10.2)	10.9 (8.9, 12.9)	8.8 (7.2, 10.4)	11.0 (9.1, 13.0)	8.6 (7.1, 10.1)	12.9 (10.5, 15.2)	10.4 (8.6, 12.3)	10.1 (8.3, 11.8)	8.7 (7.2, 10.2)	10.5 (8.6, 12.3)	8.8 (7.2, 10.3)	10.4 (8.5, 12.2)	7.8 (6.4, 9.3)	10.1 (8.3, 11.9)	7.3 (6.0, 8.6)
**140 - < 160**	12.5 (11.2, 13.8)	9.4 (8.6, 10.2)	13.3 (11.8, 14.7)	9.6 (8.7, 10.4)	11.5 (10.3, 12.7)	9.4 (8.6, 10.2)	13.2 (11.8, 14.6)	10.3 (9.4, 11.2)	13.6 (12.3, 14.9)	11.0 (10.2, 11.9)	14.0 (12.6, 15.3)	10.3 (9.5, 11.1)	10.4 (9.3, 11.5)	6.8 (6.2, 7.5)	14.0 (12.7, 15.4)	12.9 (11.9, 13.9)
**≥ 160**	12.1 (11.1, 13.1)	7.1 (6.5, 7.7)	11.2 (10.2, 12.2)	4.3 (3.8, 4.9)	13.3 (12.2, 14.4)	9.1 (8.3, 9.8)	5.2 (4.8, 5.7)	3.3 (3.0, 3.5)	18.7 (17.4, 20.1)	11.8 (10.9, 12.7)	15.3 (14.2, 16.4)	10.9 (10.1, 11.8)	12.1 (11.0, 13.2)	7.4 (6.8, 8.0)	15.6 (14.5, 16.8)	11.0 (10.2, 11.9)
**Non-HDL cholesterol — mg/dL**	17.0 (11.3, 22.6)	16.8 (12.0, 21.6)	15.3 (9.9, 20.6)	15.4 (10.8, 19.9)	17.1 (11.3, 22.9)	17.4 (12.5, 22.4)	21.4 (15.1, 27.7)	23.4 (18.0, 28.8)	16.1 (10.6, 21.6)	13.7 (9.3, 18.0)	16.9 (11.5, 22.3)	15.5 (11.2, 19.8)	16.0 (10.4, 21.6)	13.3 (8.7, 17.9)	24.4 (17.9, 31.0)	27.4 (21.9, 32.9)
**116.01 – < 154.68**	3.4 (1.2, 5.5)	3.0 (1.0, 5.0)	3.6 (1.3, 6.0)	3.1 (1.1, 5.1)	3.5 (1.3, 5.6)	3.4 (1.3, 5.5)	2.8 (1.0, 4.6)	2.6 (0.9, 4.3)	3.2 (1.1, 5.4)	2.6 (0.6, 4.6)	2.9 (1.0, 4.9)	2.3 (0.6, 4.0)	3.4 (1.1, 5.7)	3.0 (0.7, 5.3)	2.3 (0.9, 3.8)	2.2 (0.9, 3.5)
**154.68 – < 193.35**	6.2 (4.2, 8.3)	7.3 (5.6, 9.0)	5.2 (3.4, 7.0)	6.8 (5.2, 8.5)	7.0 (4.7, 9.3)	8.0 (6.2, 9.9)	7.3 (5.0, 9.7)	9.1 (7.1, 11.1)	5.7 (3.8, 7.7)	5.6 (4.2, 7.0)	5.1 (3.4, 6.8)	5.3 (4.0, 6.7)	5.9 (3.9, 7.9)	6.2 (4.6, 7.7)	7.9 (5.4, 10.4)	9.9 (7.8, 12.0)
**≥ 193.35**	7.4 (5.8, 8.9)	6.5 (5.4, 7.6)	6.4 (5.0, 7.8)	5.5 (4.5, 6.4)	6.6 (5.2, 8.1)	6.0 (5.0, 7.0)	11.3 (9.1, 13.5)	11.8 (10.0, 13.5)	7.2 (5.7, 8.6)	5.5 (4.5, 6.4)	8.9 (7.0, 10.8)	7.9 (6.6, 9.2)	6.6 (5.2, 8.0)	4.2 (3.5, 4.9)	14.2 (11.5, 16.9)	15.2 (13.1, 17.4)
**Current smoking**	6.0 (5.1, 7.0)	10.2 (9.1, 11.4)	6.2 (5.2, 7.1)	7.5 (6.6, 8.4)	5.9 (4.9, 6.8)	9.0 (7.9, 10.0)	7.7 (6.5, 9.0)	7.8 (6.9, 8.7)	3.0 (2.5, 3.4)	15.7 (14.1, 17.3)	4.4 (3.7, 5.1)	12.2 (10.9, 13.5)	5.4 (4.6, 6.2)	19.9 (17.9, 21.8)	5.2 (4.4, 6.0)	5.3 (4.6, 5.9)

Table S21a [continued].																
**Diabetes**	18.4 (16.0, 20.7)	11.6 (10.3, 13.0)	15.6 (13.6, 17.7)	12.2 (10.9, 13.6)	19.1 (16.7, 21.5)	8.9 (7.9, 10.0)	13.4 (11.7, 15.2)	8.5 (7.5, 9.4)	18.2 (15.6, 20.7)	10.0 (8.4, 11.7)	30.0 (26.6, 33.4)	21.7 (19.1, 24.3)	30.3 (26.7, 33.8)	16.8 (14.1, 19.6)	8.1 (6.9, 9.2)	6.4 (5.6, 7.1)

Population-attrbutable fractions (95% confidence intervals) are presented. Weibull models used during the computations included a single risk factor each time. Confidence interval widths have not been adjusted for multiple comparisons and should not be used in place of hypothesis testing. Non-HDL cholesterol, non-high-density lipoprotein cholesterol. To convert the values for non-HDL cholesterol from milligrams per deciliter (mg/dL) to millimoles per liter (mmol/L), multiply by 0.02586.

Table S21b. Unadjusted population-attributable fractions for 10-year all-cause mortality by risk factor categories, geographic region and sex.

	Geographic regions																	
	Global		North America		Latin America		Western Europe		Eastern Europe and Russia		North Africa and Middle East		Sub-Saharan Africa		Asia		Australia	
	Women	Men	Women	Men	Women	Men	Women	Men	Women	Men	Women	Men	Women	Men	Women	Men	Women	Men
**Body-mass index — kg/m²**	7.1 (4.5, 9.7)	0.9 (-1.4, 3.2)	7.0 (4.1, 9.9)	-1.8 (-4.8, 1.3)	5.8 (3.3, 8.4)	-2.5 (-4.8, -0.3)	5.4 (2.6, 8.2)	-5.8 (-8.8, -2.8)	7.3 (4.0, 10.6)	-3.4 (-6.2, -0.7)	10.0 (7.2, 12.8)	3.9 (1.8, 6.1)	13.0 (10.8, 15.1)	13.3 (10.7, 15.9)	14.2 (12.3, 16.0)	16.5 (13.9, 19.2)	4.2 (1.9, 6.6)	-5.5 (-8.0, -3.0)
**< 20**	3.2 (2.7, 3.6)	4.8 (4.0, 5.6)	1.8 (1.5, 2.1)	1.2 (1.0, 1.5)	2.5 (2.1, 2.9)	2.4 (2.0, 2.8)	1.5 (1.2, 1.7)	0.5 (0.4, 0.5)	1.4 (1.2, 1.7)	1.9 (1.5, 2.2)	4.2 (3.5, 4.9)	7.2 (6.0, 8.4)	8.2 (7.0, 9.4)	15.8 (13.4, 18.2)	13.8 (11.9, 15.7)	18.3 (15.7, 20.9)	2.2 (1.9, 2.6)	1.0 (0.8, 1.2)
**25 - < 30**	-1.5 (-2.8, -0.3)	-5.7 (-6.9, -4.5)	-1.5 (-2.7, -0.3)	-6.0 (-7.3, -4.6)	-1.6 (-3.0, -0.3)	-6.4 (-7.9, -5.0)	-1.7 (-3.2, -0.3)	-8.3 (-10.1, -6.4)	-1.6 (-3.0, -0.3)	-7.3 (-8.9, -5.7)	-1.3 (-2.3, -0.2)	-5.0 (-6.0, -3.9)	-0.9 (-1.6, -0.2)	-3.1 (-3.8, -2.3)	-1.1 (-1.9, -0.2)	-2.0 (-2.4, -1.6)	-1.8 (-3.3, -0.3)	-7.9 (-9.7, -6.1)
**≥ 30**	5.5 (3.8, 7.2)	1.8 (0.5, 3.1)	6.6 (4.6, 8.7)	2.9 (0.8, 5.1)	5.0 (3.4, 6.5)	1.5 (0.4, 2.6)	5.7 (3.9, 7.4)	2.0 (0.5, 3.5)	7.5 (5.2, 9.8)	2.0 (0.5, 3.5)	7.0 (4.9, 9.2)	1.7 (0.4, 3.0)	5.7 (3.9, 7.4)	0.6 (0.1, 1.0)	1.4 (1.0, 1.9)	0.2 (0.0, 0.3)	3.8 (2.6, 5.0)	1.4 (0.4, 2.4)
**Systolic blood pressure — mmHg**	12.1 (9.1, 15.2)	8.9 (6.2, 11.6)	12.1 (9.1, 15.2)	7.2 (4.6, 9.8)	12.3 (9.3, 15.3)	10.1 (7.2, 12.9)	9.3 (6.4, 12.1)	6.9 (4.1, 9.7)	16.3 (12.7, 19.9)	13.6 (10.2, 17.0)	14.6 (11.2, 18.0)	12.4 (9.2, 15.7)	16.0 (12.6, 19.4)	12.5 (9.5, 15.5)	10.7 (7.9, 13.5)	7.9 (5.4, 10.4)	14.8 (11.5, 18.2)	13.5 (10.3, 16.7)
**120 - < 140**	1.9 (0.6, 3.2)	0.2 (-1.2, 1.6)	1.9 (0.6, 3.2)	0.2 (-1.2, 1.6)	1.9 (0.6, 3.2)	0.2 (-1.1, 1.6)	2.2 (0.7, 3.7)	0.3 (-1.4, 1.9)	1.8 (0.6, 3.0)	0.2 (-1.2, 1.7)	1.8 (0.6, 3.1)	0.2 (-1.2, 1.6)	1.4 (0.5, 2.4)	0.2 (-0.9, 1.2)	1.8 (0.6, 3.0)	0.2 (-1.1, 1.5)	1.8 (0.6, 3.0)	0.2 (-1.0, 1.3)
**140 - < 160**	4.5 (3.3, 5.7)	4.2 (3.1, 5.2)	4.8 (3.5, 6.1)	4.2 (3.1, 5.2)	4.1 (3.0, 5.2)	4.1 (3.1, 5.1)	4.6 (3.4, 5.9)	4.6 (3.4, 5.7)	5.2 (3.8, 6.6)	5.2 (3.9, 6.4)	5.2 (3.8, 6.6)	4.8 (3.6, 6.0)	5.2 (3.8, 6.5)	4.5 (3.4, 5.7)	3.6 (2.6, 4.6)	3.1 (2.3, 3.9)	5.2 (3.8, 6.6)	5.9 (4.4, 7.3)
**≥ 160**	5.7 (4.9, 6.6)	4.5 (3.9, 5.1)	5.4 (4.6, 6.2)	2.8 (2.3, 3.2)	6.3 (5.4, 7.2)	5.8 (5.0, 6.5)	2.4 (2.0, 2.8)	2.0 (1.8, 2.3)	9.2 (7.9, 10.6)	8.2 (7.1, 9.3)	7.5 (6.4, 8.6)	7.4 (6.4, 8.4)	9.4 (8.0, 10.8)	7.8 (6.6, 9.0)	5.4 (4.5, 6.2)	4.6 (3.9, 5.3)	7.8 (6.7, 8.9)	7.5 (6.5, 8.5)
**Non-HDL cholesterol — mg/dL**	-10.7 (-15.8, -5.7)	-21.1 (-24.9, -17.2)	-10.0 (-14.6, -5.5)	-19.0 (-22.5, -15.6)	-11.0 (-16.1, -6.0)	-21.9 (-25.9, -17.9)	-12.1 (-18.4, -5.9)	-27.7 (-33.2, -22.3)	-10.8 (-15.8, -5.8)	-19.5 (-22.9, -16.1)	-9.9 (-14.7, -5.1)	-18.4 (-21.7, -15.1)	-9.7 (-14.0, -5.3)	-13.5 (-15.7, -11.3)	-11.2 (-16.3, -6.1)	-20.8 (-24.5, -17.2)	-12.4 (-19.3, -5.5)	-28.5 (-34.4, -22.7)
**116.01 – < 154.68**	-5.5 (-7.5, -3.5)	-10.0 (-11.6, -8.3)	-5.8 (-7.9, -3.7)	-9.7 (-11.2, -8.1)	-5.4 (-7.4, -3.5)	-10.5 (-12.2, -8.8)	-4.9 (-6.7, -3.1)	-9.9 (-11.6, -8.2)	-5.6 (-7.6, -3.6)	-10.0 (-11.6, -8.4)	-5.0 (-6.8, -3.2)	-8.5 (-9.8, -7.1)	-5.3 (-7.1, -3.4)	-6.3 (-7.2, -5.3)	-5.9 (-8.1, -3.8)	-11.6 (-13.5, -9.7)	-4.1 (-5.7, -2.6)	-8.0 (-9.4, -6.6)
**154.68 – < 193.35**	-4.1 (-5.8, -2.4)	-8.4 (-9.8, -7.1)	-3.3 (-4.7, -2.0)	-7.3 (-8.5, -6.2)	-4.6 (-6.5, -2.7)	-9.0 (-10.5, -7.6)	-5.3 (-7.6, -3.1)	-12.2 (-14.2, -10.2)	-4.1 (-5.7, -2.4)	-7.0 (-8.1, -6.0)	-3.5 (-4.9, -2.1)	-6.5 (-7.4, -5.5)	-3.5 (-4.9, -2.1)	-5.6 (-6.4, -4.8)	-4.2 (-5.9, -2.5)	-7.4 (-8.6, -6.3)	-5.9 (-8.3, -3.4)	-13.2 (-15.4, -11.0)
**≥ 193.35**	-1.1 (-2.2, 0.0)	-2.6 (-3.3, -2.0)	-0.9 (-1.9, 0.0)	-2.0 (-2.5, -1.5)	-1.0 (-1.9, 0.0)	-2.4 (-3.0, -1.8)	-1.9 (-3.8, 0.0)	-5.7 (-7.1, -4.3)	-1.1 (-2.3, 0.0)	-2.5 (-3.0, -1.9)	-1.4 (-2.8, 0.0)	-3.5 (-4.3, -2.7)	-0.9 (-1.8, 0.0)	-1.6 (-2.0, -1.3)	-1.0 (-2.1, 0.0)	-1.8 (-2.2, -1.4)	-2.4 (-4.9, 0.0)	-7.4 (-9.2, -5.5)

Table S21b [continued].

**Current smoking**	6.8 (6.1, 7.5)	14.1 (12.9, 15.2)	7.0 (6.3, 7.7)	10.5 (9.7, 11.4)	6.5 (5.8, 7.2)	12.9 (11.8, 13.9)	8.3 (7.3, 9.3)	10.2 (9.3, 11.2)	3.4 (3.1, 3.8)	22.5 (20.9, 24.2)	5.1 (4.6, 5.7)	17.0 (15.6, 18.3)	6.3 (5.6, 7.0)	14.0 (12.8, 15.2)	6.9 (6.2, 7.6)	28.3 (26.4, 30.2)	5.8 (5.2, 6.5)	6.9 (6.3, 7.6)
**Diabetes**	13.5 (12.1, 15.0)	10.8 (9.7, 11.9)	11.7 (10.5, 13.0)	11.8 (10.7, 12.9)	14.4 (12.9, 15.9)	8.5 (7.6, 9.3)	10.3 (9.1, 11.4)	8.5 (7.6, 9.3)	13.6 (11.8, 15.3)	9.6 (8.2, 11.0)	22.5 (20.0, 24.9)	19.8 (17.4, 22.1)	15.0 (12.7, 17.4)	9.6 (8.2, 10.9)	22.8 (20.1, 25.5)	15.4 (12.8, 18.0)	6.1 (5.4, 6.8)	6.2 (5.6, 6.8)

Population-attrbutable fractions (95% confidence intervals) are presented. Weibull models used during the computations included a single risk factor each time. Confidence interval widths have not been adjusted for multiple comparisons and should not be used in place of hypothesis testing. Non-HDL cholesterol denotes non-high-density lipoprotein cholesterol. To convert the values for non-HDL cholesterol from milligrams per deciliter (mg/dL) to millimoles per liter (mmol/L), multiply by 0.02586.

References

1. World Health Organization (WHO): Regional offices [webpage]. Accessed July 4, 2023. https://www.who.int/about/who-we-are/regional-offices
2. United Nations, Department of Economic and Social Affairs Population Division. Definitions of Regions. Accessed July 4, 2023. https://population.un.org/wpp/DefinitionOfRegions/
3. Evans A, Salomaa V, Kulathinal S, et al. MORGAM (an international pooling of cardiovascular cohorts). International journal of epidemiology. 2005. 34(1):21-7. DOI: 10.1093/ije/dyh327.
4 van Buuren S, Groothuis-Oudshoorn K. mice: Multivariate Imputation by Chained Equations in R. Journal of Statistical Software 2011. 45:1 - 67.
5. White IR, Royston P. Imputing missing covariate values for the Cox model. Statistics in medicine. 2009. 28:1982 - 98.
6. Schemper M, Smith TL. A note on quantifying follow-up in studies of failure time. Control Clin Trials. 1996. 17:343-6.
7. Aalen O. An Empirical Transition Matrix for Non-Homogeneous Markov Chains Based on Censored Observations. Scandinavian Journal of Statistics. 1978. 141–50.
8. Riley RD, Lesley A Stewart, and Jayne F Tierney. Individual Participant Data Meta-Analysis for Healthcare Research. Wiley Online Library. 2021. DOI:10.1002/9781119333784
9. Korn EL, Graubard BI, Midthune D. Time-to-event analysis of longitudinal follow-up of a survey: choice of the time-scale. American journal of epidemiology. 1997. 145:72 - 80.
10. Hjort NL. On Inference in Parametric Survival Data Models. International Statistical Review / Revue Internationale de Statistique. 1992. 60:355 - 387. DOI: 10.2307/140368
11. Geskus RB. Cause-specific cumulative incidence estimation and the Fine and Gray model under both left truncation and right censoring. Biometrics. 2011. 67:39 - 49. DOI: 10.1111/j.1541-0420.2010.01420.x
12. Laaksonen MA, Virtala E, Knekt P, Oja H, Härkänen T. SAS macros for calculation of population-attrbutable fraction in a cohort study design. J Stat Softw. 2011. 43:1 - 25.
13. Leong DP, Rangarajan S, Rosengren A, et al. Medications for blood pressure, blood glucose, lipids, and anti-thrombotic medications: relationship with cardiovascular disease and death in adults from 21 high-, middle-, and low-income countries with an elevated body-mass index. Eur J Prev Cardiol. 2022. 29(14):1817 - 1826. DOI: 10.1093/eurjpc/zwac069.
14. Harrell, Frank E., Regression modeling strategies: with applications to linear models, logistic regression, and survival analysis. Springer. 2015. 2:445
15. Lim SS, Vos T, Flaxman AD, et al. A comparative risk assessment of burden of disease and injury attributable to 67 risk factors and risk factor clusters in 21 regions, 1990-2010: a systematic analysis for the Global Burden of Disease Study 2010. Lancet. 2012. 380(9859):2224 - 60. DOI: 10.1016/s0140-6736(12)61766-8.
16. Cheng S, Claggett B, Correia AW, et al. Temporal trends in the population-attributable risk for cardiovascular disease: the Atherosclerosis Risk in Communities Study. Circulation. 2014. 130(10):820 - 8. DOI: 10.1161/CIRCULATIONAHA.113.008506.
17. Team, R Core R. A Language and Environment for Statistical Computing. R Foundation for Statistical Computing, Vienna, Austria (2022). Accessed May 30, 2023. https://www.R-project.org/
18. Tsao CW, Aday AW, Almarzooq ZI, et al. American Heart Association Council on Epidemiology and Prevention Statistics Committee and Stroke Statistics Subcommittee: Heart Disease and Stroke Statistics-2023 Update: A Report From the American Heart Association. Circulation. 2023. 147(8):e93 - e621. DOI: 10.1161/CIR.0000000000001123.
